# Two-sided effects of the organic phosphorus phytate on a globally important marine coccolithophorid phytoplankton

**DOI:** 10.1128/spectrum.01255-23

**Published:** 2023-09-13

**Authors:** Jiashun Li, Kaidian Zhang, Ling Li, Yujie Wang, Cong Wang, Senjie Lin

**Affiliations:** 1 Xiamen Key Laboratory of Urban Sea Ecological Conservation and Restoration, State Key Laboratory of Marine Environmental Science, and College of Ocean and Earth Sciences, Xiamen University, Xiamen, Fujian, China; 2 State Key Laboratory of Marine Resource Utilization in South China Sea, Hainan University, Haikou, Hainan, China; 3 Laboratory of Marine Biology and Biotechnology, Qingdao National Laboratory of Marine Science and Technology, Qingdao, Shandong, China; 4 Department of Marine Sciences, University of Connecticut, Groton, Connecticut, USA; Istituto Italiano di Tecnologia, Torino, Piemonte, Italy

**Keywords:** phosphorus nutrient, dissolved organic phosphorus, phytic acid, phytoplankton, coccolithophores, *Emiliania huxleyi*

## Abstract

Dissolved organic phosphorus (DOP) is a potential source of aquatic eutrophication and pollution because it can potentially stimulate growth in some species and inhibit growth in other species of algae, the foundation of the marine ecosystem. Inositol hexaphosphate (also named phytic acid or PA), an abundant organophosphate, is presumably ubiquitous in the marine environment, but how it affects marine primary producers is poorly understood. Here, we investigated the bioavailability of this DOP to the cosmopolitan coccolithophore *Emiliania huxleyi*. Our results showed that *E*. *huxleyi* cells can take up PA and dissolved inorganic phosphorus (DIP) simultaneously. Absorbed PA can efficiently support algal growth, producing cell yield between DIP and phosphorus (P)-depleted conditions. Accordingly, PA supply as the sole P source highly influences cellular metabolism and nutrient stoichiometry. Particularly, PA-grown cultures exhibited enhanced carbon fixation, increased lipid content, activated energy metabolism, and induced nitrogen assimilation. However, our data suggest that PA may also exert some levels of toxic effects on *E. huxleyi*. This study provides novel insights into the variable effects of a DOP on marine phytoplankton, which will inform new inquiries about how the complex DOP constituencies in the ocean will shape phytoplankton community structure and function.

**IMPORTANCE:**

The dissolved organic phosphorus (DOP) utilization in phytoplankton plays vital roles in cellular P homeostasis, P-nutrient niche, and the dynamics of community structure in marine ecosystems, but its mechanisms, potentially varying with species, are far from clear. In this study, we investigated the utilization of a widespread DOP species, which is commonly produced by plants (land plants and marine macrophytes) and released into coastal areas, in a globally distributed bloom-forming coccolithophore species in various phosphorus environments. Using a combination of physiological and transcriptomic measurements and analyses, our experimental results revealed the complex mechanism and two-sided effects of DOP (major algal growth-supporting and minor toxic effects) in this species, providing a novel perspective on phytoplankton nutrient regulation.

## INTRODUCTION

Phosphorus (P) is a vital macronutrient present in a variety of organic biomolecules controlling the growth of phytoplankton and other organisms directly or indirectly. In the marine ecosystem, P has two major reservoirs: dissolved inorganic phosphorus (DIP) and dissolved organic phosphorus (DOP) (1). Loading both forms of P from cultural and industrial sources can cause eutrophication in the coastal marine environment (2). Some DOP are toxic to photosynthetic organisms (e.g., glyphosate) and animals (e.g., insecticides) (3 – 5). In the ocean, the concentrations of DOP may exceed that of DIP by orders of magnitude (1, 6, 7). The oceanic DOP pool contains two major forms, phosphoester and phosphonate, accounting for about 80% and 20%, respectively (8). Phytoplankton and microbes are the major players in the biogeochemical cycle of these DOPs (6, 9). However, the bioavailability and utilization mechanism of different DOPs in phytoplankton are still limited.

Of the wide spectrum of phosphoesters, phytic acid (inositol hexaphosphate, also known as phytate in salt form; hereafter PA) is a major organic P form in land plants and marine macrophytes (10 – 13). However, PA cannot be assimilated by nonruminants due to the lack of phytase, the enzyme that hydrolyzes PA (14, 15). As a result, the large amounts of unutilized PA from animal feeds enter receiving waters as animal wastes and residues, becoming a considerable P input in the coastal ecosystem and a potential source of eutrophication (12, 16). Hence, the differential bioavailability of PA to different species of phytoplankton can potentially cause differential growth between species and, hence, algal community structure shifts.

Coccolithophores are a biogeochemically crucial group of marine phytoplankton. They fix CO_2_ through both photosynthesis and form calcium carbonate plates (coccoliths), thereby enhancing organic carbon sinking and carbon sequestration in the ocean (17, 18). *Emiliania huxleyi* is the most widely distributed and abundant coccolithophore species, and as such it is a remarkable model for studying algal ecophysiology and carbon biogeochemistry (19 – 21). Relying on the considerable metabolic plasticity in the face of environmental changes, *E. huxleyi* is capable of forming blooms (21 – 23). It has been documented that *E. huxleyi* grows well on phosphoesters such as glycerophosphate, AMP, and ATP (24, 25). However, the bioavailability of the broadly existing phosphoester PA to *E. huxleyi* as well as many other phytoplankton species remains to be examined.

Here, we investigated the physiological and transcriptomic responses of *E. huxleyi* to PA as P supply and explored the utilization mechanism of PA. Our results reveal that *E. huxleyi* can efficiently utilize PA to relieve cellular P-starvation and grow. *E. huxleyi* can simultaneously take up DIP and PA under the P-replete condition while triggering a series of cellular metabolic responses. Our results demonstrate the metabolic plasticity of *E. huxleyi* to maintain population growth and cellular nutrient homeostasis under fluctuating P nutrient environments, potentially contributing to the wide distribution of species and propensity to form blooms. Our findings provide important ecological implications in P-nutrient niche differentiation and the impacts of different DOPs on phytoplankton community composition and succession leading to algal blooms.

## RESULTS

### Algal growth and extracellular and intracellular P concentration

**Fig 1:**
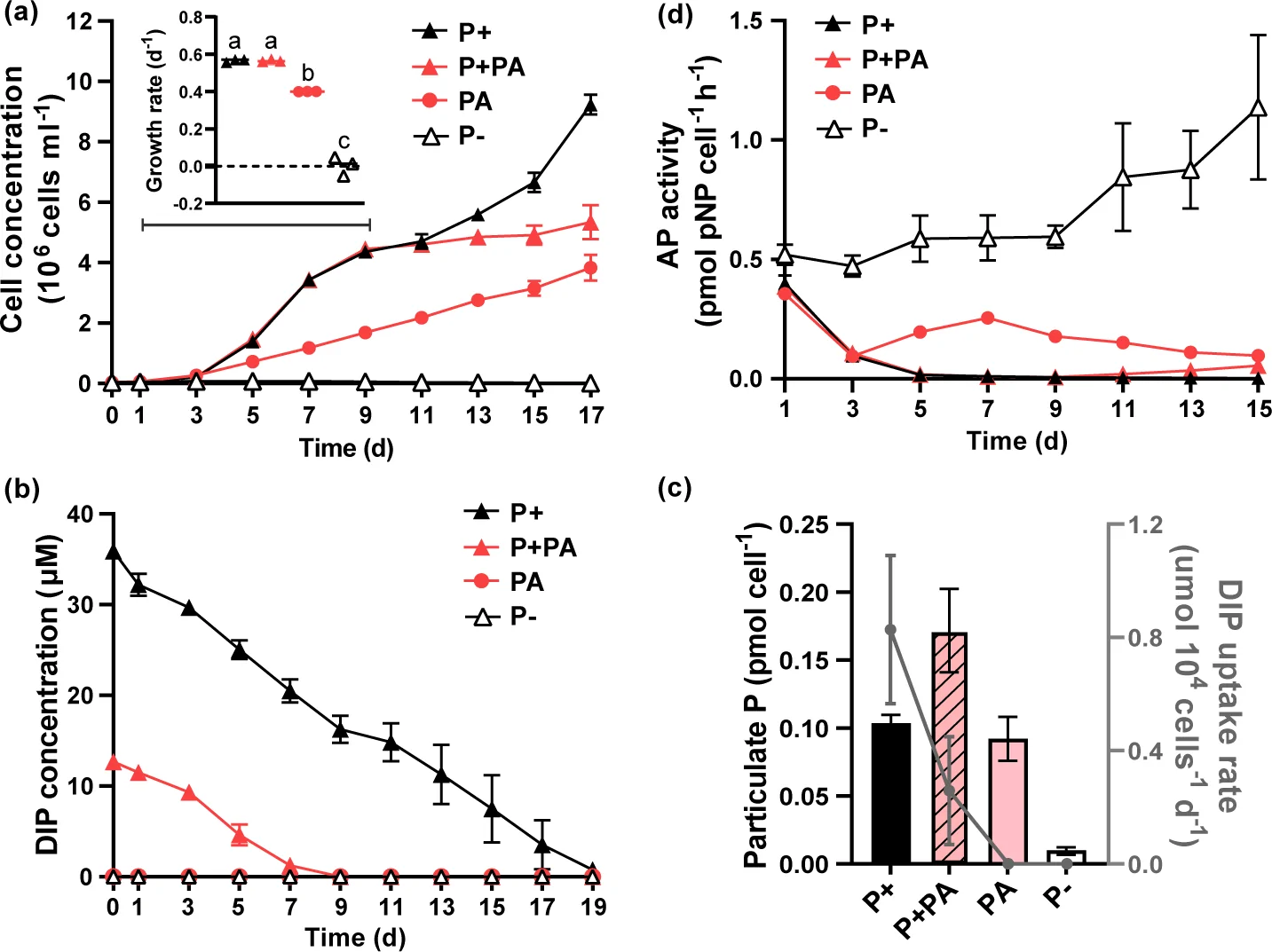
Algal growth and P nutrient of *E. huxleyi* under different P treatments. (a) Growth curves during the experiment and average growth rate (inset) during the exponential growth phase (from days 1 to day 9). (b) DIP concentration in the medium under different P conditions. (c) Cellular particulate P (PP, bar chart) and DIP uptake rate (line chart) on day 1. (d) AP activity. P−, P-depleted; PA, phytate; P+, DIP replete; P+PA, DIP and PA replete. Each data point is the mean of triplicate cultures with the error bar indicating standard deviation (Mean ± SD). Samples for transcriptomic analysis were collected on the 5^th^ day.

Starting from the cell density of 4.23 × 10^4^ cells mL^−1^, *E. huxleyi* under the four P conditions (P+, P-replete; P+PA, DIP and PA supplied together; PA, PA supplied as a sole P source; and P−, P-depleted) examined exhibited significantly different growth trajectories [repeated measures analysis of variance (ANOVA), *P* < 0.01] (Fig. 1a). *E. huxleyi* cells under the PA condition showed linear growth throughout the experimental period, with a significantly and markedly higher growth rate than P-depleted cultures (repeated measures ANOVA, *P* < 0.01), but a lower growth rate than that under the P+ condition (repeated measures ANOVA, *P* < 0.01) (Fig. 1a). Compared to growth under P+, growth under PA only reduced by 29% during the period in which the P+ cultures were in exponential growth phase (from day 1 to day 9) (Fig. 1a), indicating the efficient utilization of PA as a sole P source. Cells in the P+PA cultures showed a similar growth rate (0.57 ± 0.01 d^−1^) with the P+ group (0.57 ± 0.01 d^−1^) during the exponential growth phase, but growth of the P+PA group slowed from the 9^th^ day (when the extracellular DIP was depleted, see below) (Fig. 1a). At the end of the experiment (the 17^th^ day), cell concentration in the P+PA group reached 534.33 ± 56.42 × 10^4^ cells mL^−1^, 42% lower than that under the P+ condition (923.03 ± 32.54 × 10^4^ cells mL^−1^) (Fig. 1a; ANOVA, *P* < 0.01), nearly in the middle between P*+* and P− groups.

As the algae grew, DIP concentration in the medium decreased rapidly in both P+ and P+PA groups and depleted on day 9 in the P+PA group (Fig. 1b). DIP was below detection limit in the PA group throughout the experiment (Fig. 1b). On day 1, DIP uptake rate in the P+PA group was significantly lower than in the P+ group (ANOVA, *P* < 0.05), while particulate P (PP) in the P+PA group was 0.17 ± 0.03 pmol cell^−1^, close to the sum of that in PA (0.09 ± 0.02 pmol cell^−1^) and P+ groups (0.10 ± 0.01 pmol cell^−1^) (Fig. 1c). This indicates that cells in the P+PA cultures could absorb DIP and PA in the medium simultaneously. The cultures provided P nutrients, including the PA, P+, and P+PA groups, exhibited decreasing alkaline phosphatase (AP) activities over time, in contrast to the increasing AP activity in the P-depleted group (Fig. 1d). However, cells in the PA group showed significantly higher AP activity than that in the P+ group after day 5 (repeated measures ANOVA, *P* < 0.01) (Fig. 1d). In addition, AP activity in the P+PA group also began to show a significant increase compared with the P+ group on day 11 (ANOVA, *P* < 0.01) (Fig. 1d).

### Photosynthesis efficiency, pigment content, lipid content, and cell size

**Fig 2:**
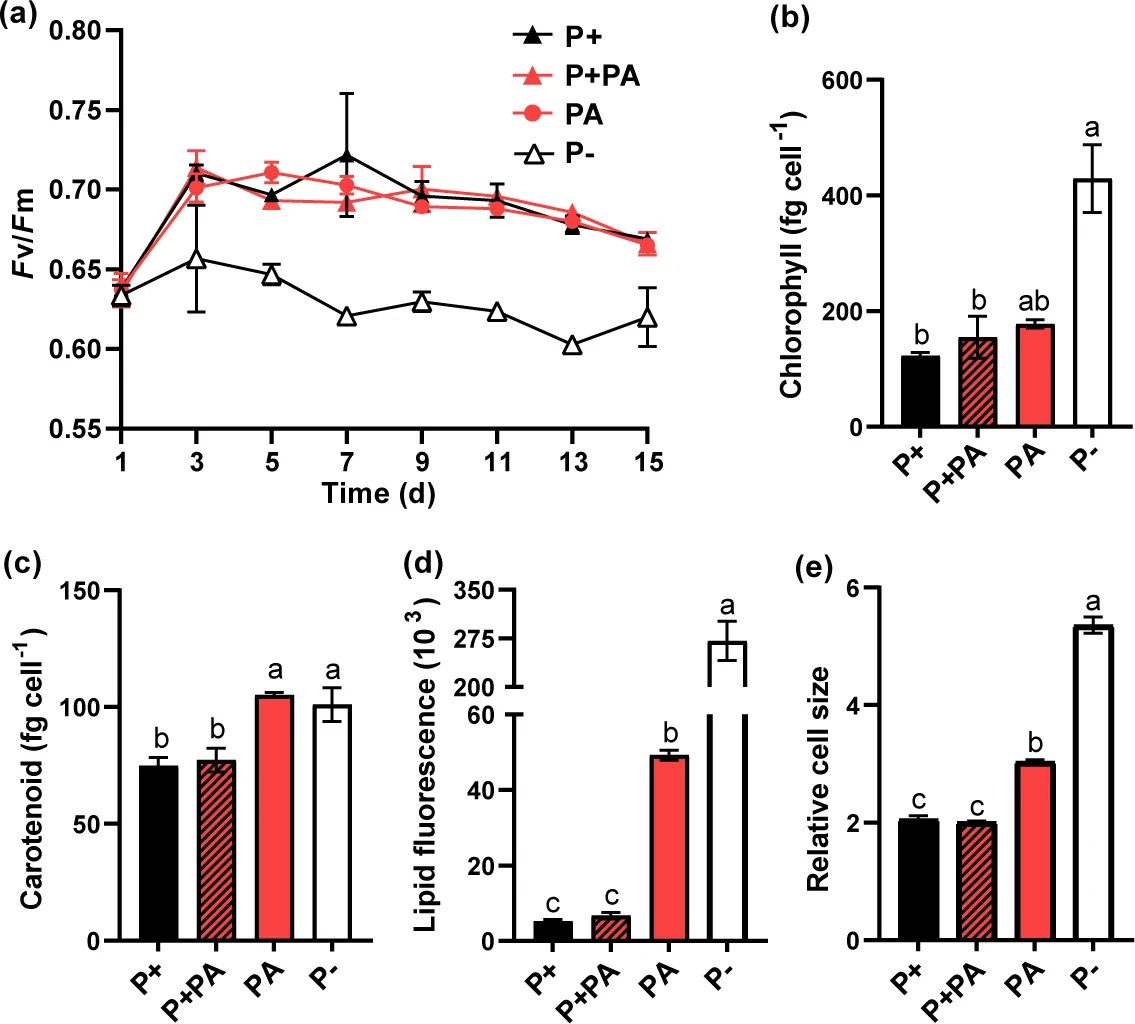
Physiological responses of *E. huxleyi* under different P treatments. (a) Photosynthetic efficiency (*F*v/*F*m); (b) Chlorophyll content; (c) Carotenoid content; (d) Lipid content measured as ﬂuorescence intensity of BODIPY 505/515 stain. (e) Relative cell size of *E. huxleyi* estimated by normalizing to forward scatter (FSC) of 2 µm standard beads. Each data point is the mean of triplicate cultures with the error bar indicating standard deviation (Mean ± SD). Different letters above the columns indicate signiﬁcant differences among groups (ANOVA, *P* < 0.05). Pigments, lipid contents, and relative cell size were measured on the 5^th^ day.

Immediately after P nutrient was added (day 0), the photosynthetic efficiency (*F*v/*F*m) in PA, P+, and P+PA groups recovered rapidly compared with P− group and remained at a similar level from day 3 (Fig. 2a). Chlorophyll contents in PA, P+, and P+PA groups showed similar levels and were more than 59% lower than that in the P− group (Fig. 2b). Meanwhile, the chlorophyll contents in PA and P+PA groups were higher than that in the P+ group (albeit not significant) (Fig. 2b). The carotenoid content in the PA group was 41% and 36% higher than that in P+ and P+PA groups, respectively (ANOVA, *P* < 0.01 in both comparisons) (Fig. 2c).

The cellular lipid contents (determined by the lipid fluorescence) in the P− group were dramatically higher than that under other three P-supplied conditions (Fig. 2d). Among the P-supplied cultures, the lipid content under the PA condition were 8.4-fold higher (ANOVA, *P* < 0.01) than that under the P+ condition, and the lipid content of the P+PA cultures was 28% higher (albeit not significant) than that under the P+ condition (Fig. 2d). Cell size in the P− group was significantly higher than that in PA, P+, and P+PA groups (ANOVA, *P* < 0.01 in all comparisons) (Fig. 2e; Fig. S1). The PA group showed significantly larger cell size than P+ and P+PA groups (ANOVA, *P* < 0.01 in both comparisons) (Fig. 2e).

### Particulate carbon, nitrogen content, and cellular stoichiometry

**Fig 3:**
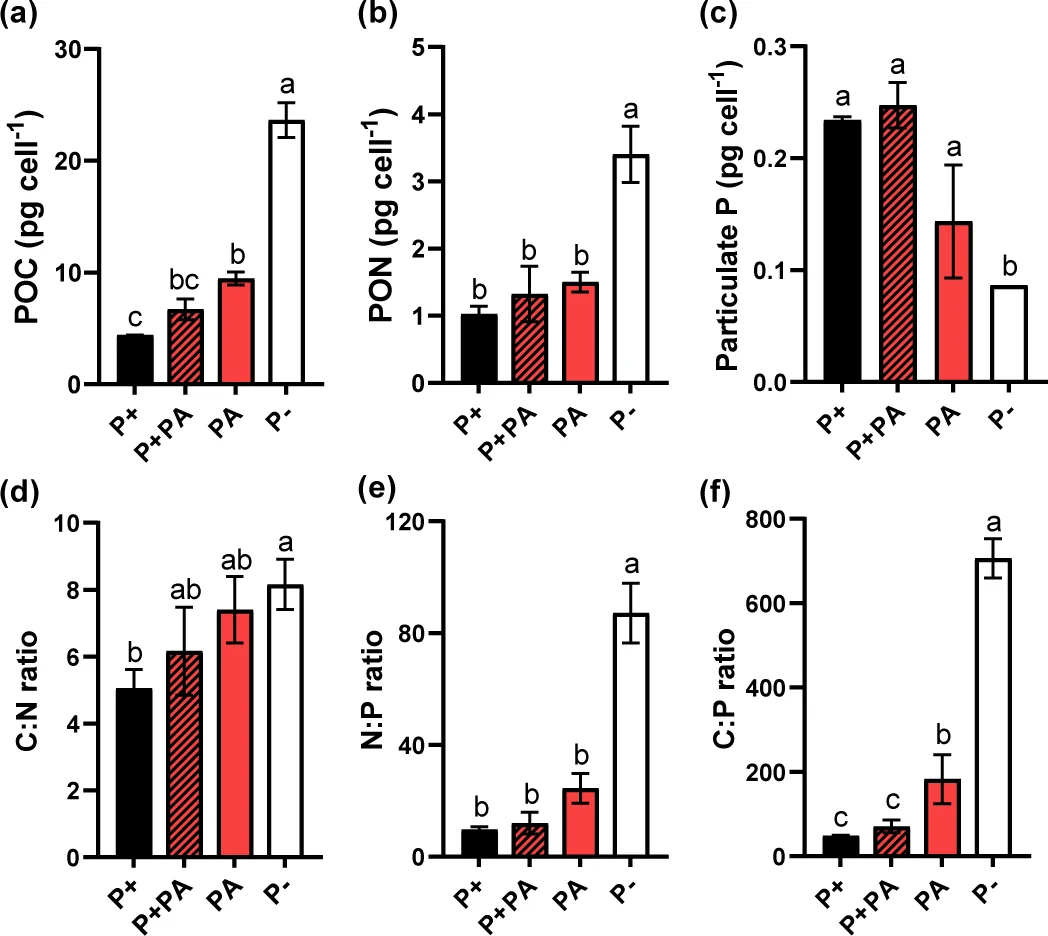
Cellular stoichiometry of *E. huxleyi* under different P conditions measured on the 5^th^ day. (a) POC content; (b) Particulate organic nitrogen (PON) content; (c) Particulate P; (d) C:N ratio; (e) N:P ratio; (f) C:P ratio. Each data point is the mean of triplicate cultures with the error bar indicating standard deviation (Mean ± SD). Different letters above the columns indicate signiﬁcant differences among groups (ANOVA, *P* < 0.05).

The particulate organic carbon (POC) and inorganic carbon (PIC) contents under PA, P+, and P+PA conditions were significantly lower than that under the P− condition (ANOVA, *P* < 0.05) (Fig. 3; Fig. S2). Meanwhile, POC contents in PA and P+PA groups were 1.2-fold and 53% higher than that under the P+ condition, respectively (ANOVA, *P* < 0.05 in both comparisons) (Fig. 3a). Similarly, the PIC contents in PA and P+PA groups were higher than that under the P+ condition, albeit not significantly (Fig. S2).

Similar to cellular carbon contents, the particulate organic nitrogen (PON) contents under PA, P+, and P+PA conditions were significantly lower than that under the P− condition (ANOVA, *P* < 0.05) (Fig. 3b). Furthermore, PON contents under PA and P+PA conditions were 47% and 29% higher than that under the P+ condition, respectively (ANOVA, *P* < 0.05 in the comparison between PA and P+ groups) (Fig. 3b). Meanwhile, PP under PA, P+, and P+PA conditions on the 5^th^ day was significantly higher than that under the P− condition (ANOVA, *P* < 0.05) (Fig. 3c). Cells grown on the PA cultures showed significantly lower PP than cells grown on the P+ cultures (ANOVA, *P* < 0.05) (Fig. 3c).

Correspondingly, the cellular C:N ratio was highest in the P− group, while PA and P+PA groups showed 47% and 22% higher C:N ratios than the P+ group, respectively (ANOVA, *P* < 0.05 in the comparison between PA and P+ groups) (Fig. 3d). The N:P and C:P ratios under the P− condition were significantly higher than that under PA, P+, and P+PA conditions (ANOVA, *P* < 0.05 in both comparisons) (Fig. 3e and 3f). Meanwhile, PA and P+PA groups showed 1.5-fold and 24% higher N:P ratio than the P+ group, respectively (ANOVA, *P* < 0.01 in the comparison between PA and P+ groups) (Fig. 3e). The C:P ratio in PA and P+PA cultures was 2.8-fold and 46% higher than that in the P+ cultures, respectively (ANOVA, *P* < 0.05 in the comparison between PA and P+ groups) (Fig. 3f).

### Transcriptomic responses in *E. huxleyi* after PA addition

Transcriptome sequencing was conducted for samples collected on the 5^th^ day, when the cultures were in the exponential growth phase. Each sample was sequenced to yield 42.5 M clean data on average (Table S1). When all sequenced transcriptomes were pooled and clustered to remove redundancy, 36,293 expressed unique genes were obtained in total. The transcriptomes of PA-containing groups (PA and P+PA groups) were compared with the P+ group to determine the utilization and metabolic mechanism of PA in *E. huxleyi* under different P conditions (Table S2). In the PA/P+ comparison, 4,153 differentially expressed genes (DEGs) were detected (Fig. S3), accounting for 12% of detected expressed genes, and enriched to three KEGG metabolic pathways, which focused on carbon fixation and amino acid metabolism (Table S3). In contrast, 6,412 DEGs were detected in the (P+PA)/P+ comparison (Fig. S3), accounting for 18% of detected expressed genes, and enriched to 19 KEGG metabolic pathways, which were mainly involved in photosynthesis, carbon metabolism, amino acid metabolism, nitrogen metabolism, oxidative phosphorylation, and fatty acid metabolism (Table S4). Overall, carbon fixation and amino acid metabolism were highly induced in two PA-containing groups.

**Fig 4:**
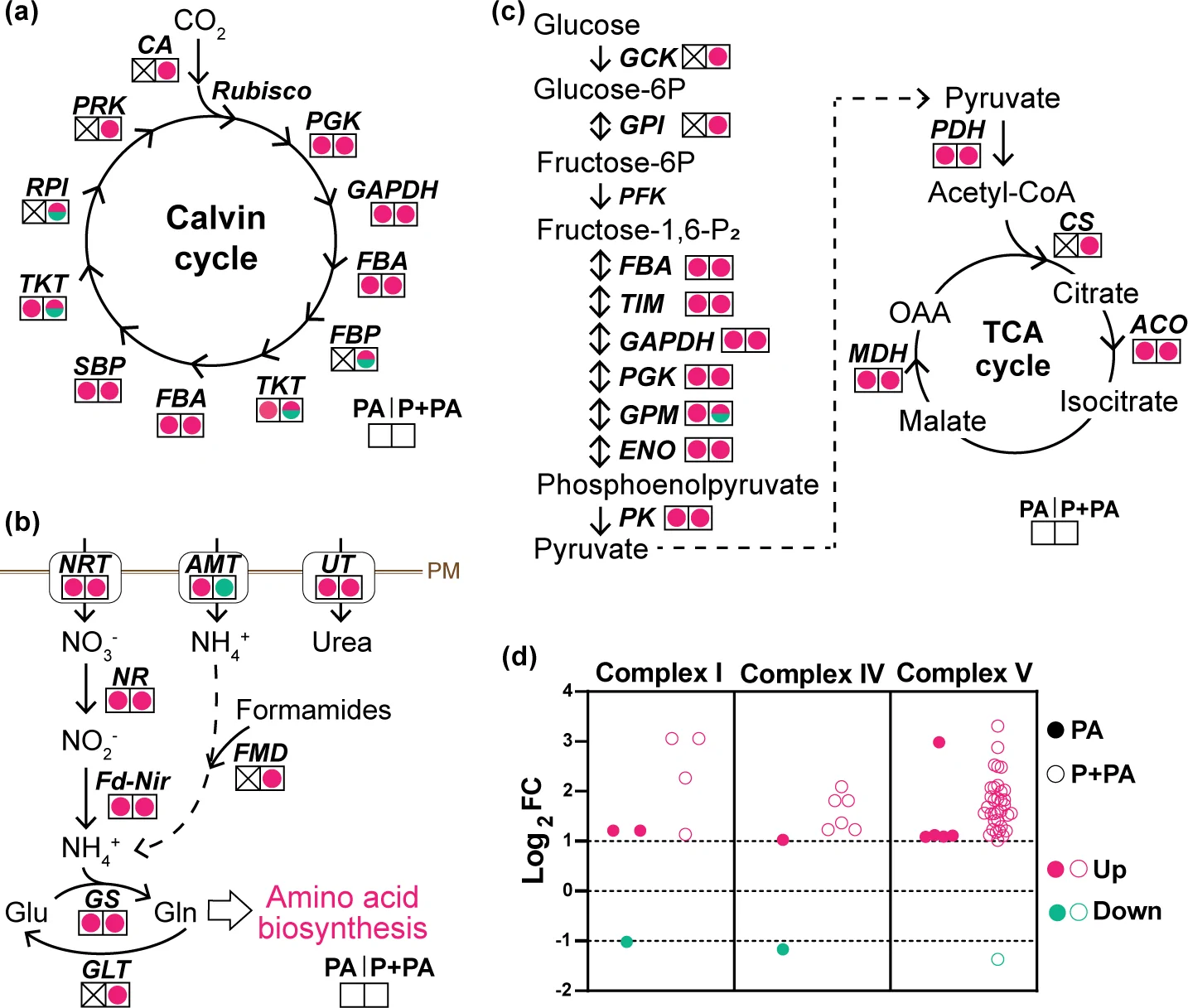
Transcriptomic responses of *E. huxleyi* to PA utilization. (a) DEGs related to Calvin cycle in PA/P+ and (P+PA)/P+ comparisons. CA, carbonic anhydrase; Rubisco, rubisco ribulose-1,5-bisphosphate carboxylase; PGK, phosphoglycerate kinase; GAPDH, glyceraldehyde 3-phosphate dehydrogenase; FBA, fructose-bisphosphate aldolase; FBP, fructose-1,6-bisphosphatase; TKT, transketolase; SBP, sedoheptulose-bisphosphatase; RPI, ribose 5-phosphate isomerase; PRK, phosphoribulokinase. (b) DEGs related to nitrogen uptake and assimilation in PA-grown *E. huxleyi* cells relative to cells in the P+ group. PM, plasma membrane; NRT, nitrate transporter; AMT, ammonium transporter; UT, urea transporter; NR, nitrate reductase; Fd-Nir, ferredoxin-nitrite reductase; FMD, formamidase; GS, glutamine synthetase; GLT, glutamate synthase. (c) DEGs related to EMP-tricarboxylic acid cycle (TCA) pathway in PA-containing groups compared to the P+ group. GCK, glucokinase; GPI, glucose-6-phosphate isomerase; PFK, phosphofructokinase; FBA, fructose-bisphosphate aldolase; TIM, triosephosphate isomerase; GAPDH, glyceraldehyde 3-phosphate dehydrogenase; PGK, phosphoglycerate kinase; GPM, phosphoglycerate mutase; ENO, enolase; PK, pyruvate kinase; PDH, pyruvate dehydrogenase; CS, citrate synthase; ACO, aconitate hydratase; MDH, malate dehydrogenase; OAA, oxaloacetate. (d) Regulation of oxidative phosphorylation in PA and P*+*PA groups relative to the P+ group. Complex I includes NADH dehydrogenase; Complex IV includes cytochrome *c* oxidase; and Complex V includes F-type ATPase, V-type ATPase, H+-transporting ATPase, and inorganic pyrophosphatase. The left and right squares indicate expression regulation in PA/P+ and (P+PA)/P+ comparisons, respectively. Berry and lawngreen cycles, respectively, indicate significant upregulated and downregulated in PA/P+ and (P+PA)/P+ comparisons. Crossed squares indicate no significant differential expression in PA/P+ or (P+PA)/P+ comparisons.

For carbon fixation, photosynthesis-related genes were upregulated in the (P+PA)/P+ comparison, including 88% of DEGs encoding light-harvesting complex and all 16 DEGs were genes involved in PSII, PSI, cytochrome *b_6_*/*f* complex, photosynthetic electron transport, and F-type ATPase (Table S5). In addition, the carbonic anhydrase, the core of the carbon concentration mechanism (CCM), also showed upregulation in both PA/P+ (albeit not significant) and (P+PA)/P+ comparisons (Fig. 4a). Meanwhile, 100% and 83% of DEGs genes involved in Calvin cycle, including phosphoglycerate kinase, glyceraldehyde 3-phosphate dehydrogenase, fructose-bisphosphate aldolase, and sedoheptulose-bisphosphatase, were being induced in PA-containing groups (Fig. 4a; Table S6).

For the amino acid metabolism, more than 87% of DEGs involved in the biosynthesis of amino acids were upregulated in PA-containing groups (Table S7). In the nitrogen assimilation, the nitrate transporter, nitrate reductase, ferredoxin-nitrite reductase, and glutamine synthetase, which collaborated with nitrogen uptake and assimilation, were all significantly upregulated in PA-containing groups (Fig. 4b; Table S8). Besides, we also observed significantly upregulated ribosome and ribosome biogenesis-related genes in PA-containing groups (Table S9).

Meanwhile, carbohydrate catabolism was also promoted in PA-containing groups (Fig. 4c). For the glycolysis, two genes encoding pyruvate kinase, the key enzyme contributing to the pyruvate production, were significantly upregulated in PA-containing groups (Fig. 4c; Table S10). In the citrate cycle [tricarboxylic acid cycle (TCA)], two key enzymes, pyruvate dehydrogenase complex and malate dehydrogenase, were both significantly upregulated in PA-containing groups (Fig. 4c; Table S11). Another key enzyme responsible for citrate production, citrate synthase, was significantly upregulated in the P+PA group (Fig. 4c). Meanwhile, over 80% of DEGs belonging to complex I to complex V in oxidative phosphorylation were upregulated in PA-containing groups (Fig. 4d; Table S12).

## DISCUSSION

Phytic acid is believed to be a widely occurring DOP in the coastal ocean, but its ecological effects are still poorly understood. The bioavailability of PA to phytoplankton has only been studied and verified in the diatom *Phaeodactylum tricornutum* (26). In this diatom, PA could support growth as the sole source of P nutrient, but in the meantime showed signals of negative effects on the availability of iron due to its binding capacity to metal ions (26). *E. huxleyi* is a widely distributed coccolithophore that can form blooms in the global ocean where nutrient supplies vary remarkably (19, 21), suggesting its superior ability to adapt to the fluctuant nutrient environment and potentially excellent DOP utilization capability (27, 28). The bioavailability of PA to cosmopolitan phytoplankton can play vital roles in the marine nutrient cycle. In the present study, we conducted a comprehensive investigation of the effects of PA and revealed the efficient PA utilization and synchronous absorption of PA and DIP in *E. huxleyi* cells, providing evidence of this species’ versatility to variable DIP and DOP environments. Our data also indicated some toxic effects of PA on this species. The opposing effects are ecologically and biogeochemically significant and deserve some in-depth discussions.

### PA could be utilized efficiently by *E. huxleyi* to support growth

As with the case of the diatom *P. tricornutum* (26, 29), no DIP was detected in the medium of the PA group of *E. huxleyi* cultures (Fig. 1b). This suggests that *E. huxleyi* could absorb PA into cells without (or very limited) extracellular hydrolysis. Since we observed dramatically raised PP in the PA group as soon as on day 1 (Fig. 1c), rapid PA uptake of *E. huxleyi* is evident. Meanwhile, the P+PA group showed lower DIP uptake but higher PP than that in the P+ group on day 1 (Fig. 1c), indicating that *E. huxleyi* could absorb PA and DIP simultaneously and the PA uptake is not affected by the presence of DIP. This is striking because in the conventional view, DIP is preferentially absorbed by phytoplankton (30). After the absorption, PA can efficiently support *E. huxleyi* growth. The growth rate of the PA cultures during the exponential phase was significantly higher than the P-depleted cultures, indicating that PA could support *E. huxleyi* growth as a sole P source.

**Fig 5:**
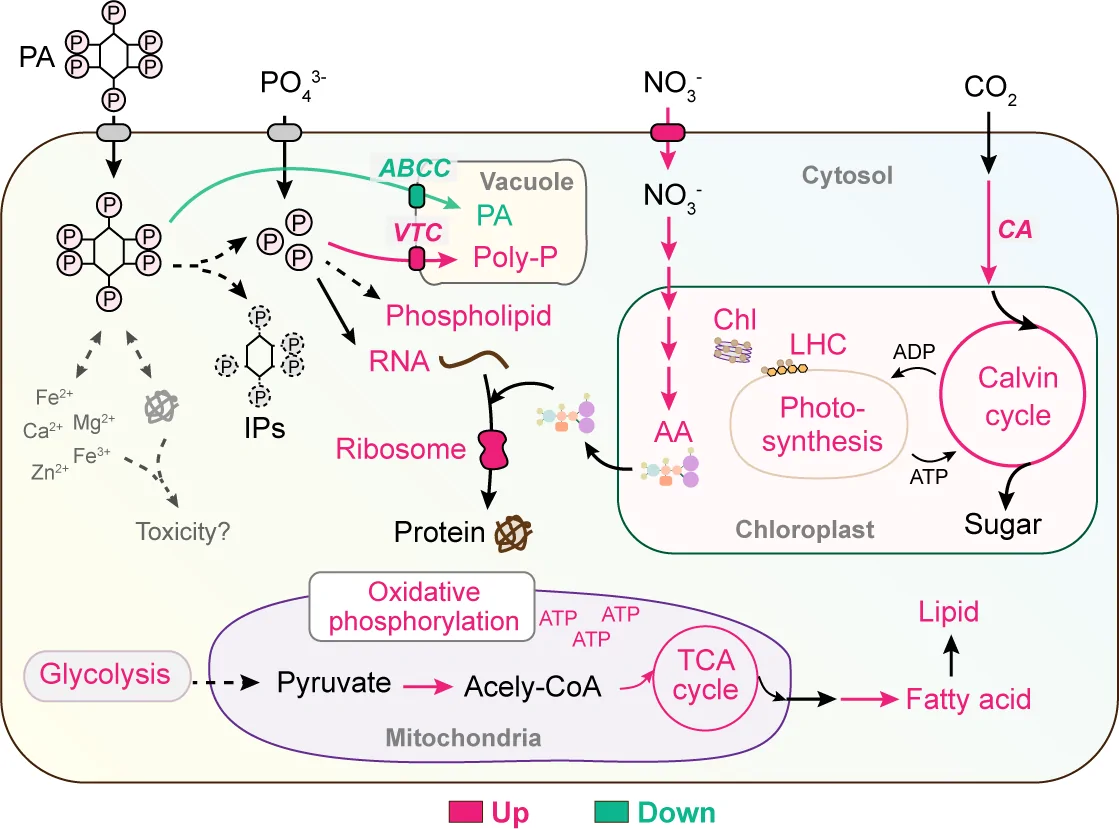
Proposed PA utilization mechanism in *E. huxleyi*. Berry and lawngreen words (and arrows) indicate upregulated and downregulated processes, respectively, in PA-grown *E. huxleyi* cells. PA, phytate; IPs, inositol phosphates; Poly-P, polyphosphate; LHC, light-harvesting complex; AA, amino acid; VTC, vacuolar transport chaperone; ABCC, ATP-binding cassette subfamily C; CA, carbonic anhydrase. The encircled P represents phosphate.

Meanwhile, the growth rate of PA-grown *E. huxleyi* cells was only 29% lower than the P-replete cultures (Fig. 1a). As a comparison, PA-grown *P. tricornutum* showed a 52% lower growth rate than DIP-grown cultures (26). The difference indicates a higher PA utilization efficiency in *E. huxleyi* than in *P. tricornutum*. This also raises the question of whether *E. huxleyi* cells could store PA. In plants, cells can synthesize PA (inositol hexaphosphate, IP_6_) from lower inositol phosphates (IPs) and transport PA into the vacuole by ATP-binding cassette subfamily C (ABCC) (31 – 33). We identified two ABCC genes from the genome of *E. huxleyi*. However, these two ABCC genes were significantly downregulated in PA-grown *E. huxleyi* cells (Fig. S4), indicating that the absorbed PA is probably hydrolyzed intracellularly for utilization instead of transported into the vacuole via ABCC (Fig. 5). In contrast, one ABCC (Phatr3_J9180) was significantly upregulated in PA-grown *P. tricornutum* cells (26), suggesting the potential vacuole PA storage in *P. tricornutum* when PA is supplied as a P source. This further demonstrates that after PA addition as a P source, intracellular hydrolysis and utilization are the dominant sinks of PA in *E. huxleyi*, while in *P. tricornutum* cells, PA goes to both hydrolysis and vacuolar storage. This explains why a higher growth rate occurs in PA-grown *E. huxleyi* than in PA-grown *P. tricornutum*. The contrast between the two algal species has important ecological implications in differential niches of DOP utilization between phytoplankton species and phytoplankton community succession and bloom formation in response to changes in the P-nutrient condition.

### PA utilization in *E. huxleyi* alters cellular metabolism and nutrient stoichiometry

The PA utilization in *E. huxleyi* dramatically influences nutrient metabolism (Fig. 5). First, we observed elevated RNA content in PA-containing cultures (Fig. 5; Fig. S5). RNA is a dominant component of cellular P quota and could reflect P nutrient status in phytoplankton (34, 35). In addition, the vacuolar transport chaperone, which is responsible for the poly-phosphate (Poly-P) synthesis (36), was also upregulated in the PA group (Fig. 5; Fig. S4), indicating the enhanced poly-P synthesis in PA-grown *E. huxleyi* cells. Poly-P has been widely considered to be actively involved in the regulation of intracellular P homeostasis (9, 37). Therefore, this further confirms that after uptake by cells, PA is hydrolyzed to generate Pi and subsequently promote the synthesis and vacuole storage of poly-P, rather than the direct ABCC-mediated vacuole PA storage (Fig. 5). Furthermore, more than 64% of DEGs involved in glycerophospholipid metabolism were upregulated in PA-containing groups (Table S13). Phospholipid accounts for a substantial fraction of cellular P, and the proportion of phospholipids in total polar lipids was shown to increase after replete P nutrient was supplied in *E. huxleyi* (23, 38). Our results suggest the promotion of phospholipid metabolism in response to PA utilization in *E. huxleyi* cells (Fig. 5). It is worth noting that the oxidative phosphorylation was induced in PA-grown *E. huxleyi* cells, resulting in enhanced ATP production (Fig. 4d and 5). This finding suggests that the Pi supplement by intracellular PA hydrolysis contributes to ATP synthesis in cells (Fig. 5). Previously, researchers have found that cellular P quota in phytoplankton is mainly distributed into RNA-P, lipid-P, poly-P, and surplus P (35, 38). In the present study, despite the observed increases in RNA, poly-P storage, phospholipid metabolism, and ATP production in the PA group compared with the P+ group, the cellular P content in the PA cultures was lower than that in the P-replete cultures (Fig. 1c and 5). These results suggest that PA utilization induces the redistribution of cellular P, investing more toward RNA, poly-P, and phospholipids (Fig. 5).

Cellular stoichiometry also appears to be affected by PA utilization in *E. huxleyi*. Since we observed upregulated Calvin cycle gene expression in PA-grown *E. huxleyi* cells, photosynthesis C fixation likely increased, explaining the elevated POC content and C:P ratio (Fig. 3 to 5). Similarly, enhanced expression of N uptake and assimilation genes is consistent with the increasing PON content and N:P ratio in PA-grown *E. huxleyi* cells (Fig. 3 to 5). Furthermore, the C:N ratio in PA-grown *E. huxleyi* cells also increased, which together with the rises of POC and PON further indicates a highly elevated cellular C metabolism (Fig. 3 and 5). The consequent changes in the cellular stoichiometry underscore the strong effects of P nutrition in cellular stoichiometric regulation in phytoplankton.

### PA is potentially toxic to *E. huxleyi*

In our previous study, we noticed several cellular stress symptoms in PA-grown *P. tricornutum* cells, including decreased growth rate, increased AP activity, decreased photosynthesis efficiency, signs of P-limitation, and upregulated iron-starvation-induced genes as a sign of iron limitation (26). To explore PA’s potential toxic effects in *E. huxleyi*, we compared the PA-only (PA group) and PA-with-DIP (P+PA group) cultures (collectively called PA-containing groups) in this study. To our surprise, the P+PA group showed various similar metabolic stress symptoms as the PA group (Fig. 4 and 5). First, cells grown on both PA-containing groups exhibited elevated lipid content (Fig. 2d and 5). Correspondingly, the acetyl-CoA carboxylase, which catalyzes fatty acid synthesis from acetyl-CoA, was upregulated in PA-containing groups (Fig. S4). In addition, the induced glycolysis and TCA cycle in PA-containing groups indicate accelerated energy production (Fig. 5), which is generally required to cope with various environmental stress (39 – 41). Hence, cells grown on PA-with-DIP cultures showed similar stress-induced responses to cells grown on PA-only cultures, even though there was an abundant supply of DIP, suggesting the toxic effects of PA.

It has been widely reported that PA has a high affinity with proteins and metal cations (such as calcium, zinc, iron, and magnesium), and restrains dietary minerals and protein bioavailability (42 – 44). For that reason, PA is considered an antinutrient for nonruminant animals, causing reduced nutrient digestibility and increased maintenance protein and energy costs (15, 45). Therefore, the multiple stress responses of *E. huxleyi* during PA utilization, including the reduced growth rate under the PA relative to the DIP conditions, are probably attributable to the cellular toxicity of PA (Fig. 5). The potential double-sided roles of PA warrant further investigation into the interaction between PA and metal ions or proteins in phytoplankton.

### Conclusions

In this study, we investigated the physiological and metabolic responses of *E. huxleyi* to PA supply as the sole source of P nutrient. Our results showed that *E. huxleyi* cells could absorb PA directly without extracellular hydrolysis and utilize PA to support algal growth. Furthermore, to our surprise, *E. huxleyi* cells could take up PA and DIP simultaneously with equal efficiency, challenging the canon that DIP is always the preferred P form for phytoplankton to absorb. PA utilization in *E. huxleyi* influences cellular metabolism and nutrient stoichiometry. PA-grown *E. huxleyi* cells exhibit consistent metabolic responses including enhanced carbon fixation, activated energy metabolism, increased lipid accumulation, and induced nitrogen assimilation. In addition, our data also suggest that PA may also exert some levels of toxic effects on *E. huxleyi*. This study sheds light on the highly complex strategies and mechanisms by which phytoplankton utilize DOPs and provides a new perspective to understand P-nutrient niche differentiation among species that has implications for community succession in phytoplankton.

## MATERIALS AND METHODS

### Algal culture and basic setup

The *E. huxleyi* strain PML B92/11 was obtained from the Collection Center of Marine Algae (Xiamen University). Algal cultures were incubated in the L1 medium prepared with 0.22 µM filtered autoclaved seawater with a light intensity of 110 µE m^−2^ S^−1^ under a L:D cycle of 14:10 h at 20°C (46). Before the experiment, *E. huxleyi* cells in the exponential phase were transferred into an L1 medium without the P nutrient for one week to exhaust intracellular P storage. Then, the pre-P-deprived cells were cultured in the modified L1 media to provide four different P conditions: 36 µM DIP supplied (P+ group), 12 µM PA supplied (PA group), 12 µM PA plus 12 µM DIP supplied (P+PA group), and neither DIP nor DOP added (P− group). The batch cultures were set up in triplicate. These P-nutrient regimens were based on our preliminary experiments showing that the algal growth was saturated at 12 µM DIP combined with 12 µM PA (Fig. S6). Besides, an antibiotic cocktail (containing 100 µg mL^−1^ ampicillin, 50 µg mL^−1^ kanamycin, and 50 µg mL^−1^ streptomycin) was added to remove the effects of bacteria.

### Algal growth and cell size measurement

Cell concentration was counted daily using a ﬂow cytometer (CytoFLEX, Beckman Coulter, USA). Algal growth rate (*μ*) was calculated according to *μ* = ln(*N*_1_/*N*_0_)/(*t*_1_ − *t*_0_), where *N*_1_ and *N*_0_ represent the cell concentration at time 1 (*t*_1_) and time 0 (*t*_0_), respectively. The forward scatter of cells in each culture was determined using a flow cytometer and normalized to standard fluorescent beads (2 μm) to estimate the relative cell size. Meanwhile, microscopy observation of *E. huxleyi* was carried out using Zeiss microscopy Axio Imager A2 (Carl Zeiss, Germany) on the 5^th^ day after incubation.

### Photosynthetic efficiency and pigment content measurement

Every two days, two milliliters of culture were placed in darkness for 20 min, then the photosynthetic efficiency quantified as the maximum PSII quantum yield (*F*v/*F*m) was measured using a Fluorescence Induction and Relaxation Fluorometer System (Satlantic, Halifax, Canada) (47). Pigment contents were measured on the 5^th^ day. About 10^7^ cells were collected from each culture through filtration onto a 25-mm GF/F membrane. The membranes were immersed into five milliliters pure methanol and kept in darkness overnight at 4°C to extract chlorophyll and carotenoid. After centrifugation at 5,000 × *g* for 10 min, the supernatants were separated for the absorption spectra scan in a spectrophotometer (Agilent Technologies, USA). Then, the cellular contents of chlorophyll a, chlorophyll c, and carotenoid were calculated as previously reported (48, 49).

### DIP concentration in the medium and cellular particulate P

DIP concentration in the medium was measured periodically throughout the experiment. Twenty milliliters of each culture was filtered through a 0.22-µM mixed-cellulose-ester membrane, then the filtrate was used to determine the DIP concentration by a phosphorus molybdenum blue spectrophotometry (50, 51). About 10^6^ cells from each culture were filtered onto a precombusted (combusted in a Muffle Furnace at 450°C over 5 h) 25-mm GF/F filter for PP measurement. Then, each membrane was autoclaved at 121°C for 30 min with 5% acid potassium persulfate (K_2_S_2_O_8_). The DIP concentration in the solution was determined (50, 51) and then converted to per-cell PP content.

### Alkaline phosphatase activity quantification

Bulk AP activity was measured throughout the experiment. One milliliter of culture with 20 mM 4-nitrophenyl phosphate (dissolved in 1 M Tris pH 8.5 buffer) was incubated in darkness for 2 h at 20°C (52). Then samples were centrifuged at 10,000 × *g* for 2 min and the absorbance of supernatants at 405 nm was measured on a SpectraMax Paradigm plate reader and normalized to AP activity per cell (Molecular Devices, USA).

### Cellular carbon and nitrogen measurement

About 10^7^ cells from each culture were filtered onto a precombusted 25-mm GF/F filter. Then, these cell-containing ﬁlters were dried at 55°C in a clean oven. Two sets of samples were prepared, one for total particulate carbon (TPC) and the other for POC and PON. The set for POC and PON was fumed in HCl (1%) to remove inorganic carbon, whereas the sample for TPC bypassed this step (53). Next, all samples and blank filters were encapsulated in tin foil sheets and completed elemental analysis using a Vario EL cube elemental analyzer (Elementar, Germany). After that, PIC was derived as the difference between TPC and POC.

### Lipid content measurement

The lipid contents were measured on the 5^th^ day. One milliliter of culture (containing about 10^6^ cells) was stained by BODIPY 505/515 (100 µg mL^−1^; Cayman Chemical, USA) DMSO solution and kept in darkness for 20 min at 25°C. Then per-cell fluorescence intensity was measured with 488 nm excitation and 510 nm emission on a flow cytometer (54).

### Gene expression analysis

About 5 × 10^7^ cells from each culture were centrifugated (5,000 rpm, 4°C, 10 min) and immediately suspended in the TRIzol Reagent (Invitrogen, Carlsbad, CA, USA). RNA extraction was carried out with the Direct-zol RNA MiniPrep Kit (Zymo Research, USA) following the manufacturer’s protocol (55). The concentration and quality of RNA samples were determined by a NanoDrop ND-2000 Spectrophotometer (Thermo Scientific, Wilmington, USA). Since we observed a divergent growth curve of four groups on the 5^th^ day, the RNA samples of the 5^th^ day were selected for subsequent RNA-seq sequencing under the DNBSEQ MGI2000 platform (BGI Genomics Co., China). The RNA extracts were subjected to mRNA enrichment, mRNA fragment and reverse transcription, end repair, A-tail addition and adaptor ligation, PCR amplification, denaturation and cyclization, and sequencing, following the stand Illumina RNA-seq protocol.

Raw sequencing data were filtered using SOAPnuke (version 1.4.0; parameter -l 15 -q 0.2 n 0.1) (56) by removing adaptor-polluted, low-quality, and ambiguous base (N)-rich (>5%) reads. The resultant high-quality clean reads were mapped to the *E. huxleyi* CCMP1516 genome (20) downloaded from Ensembl Genomes (https://protists.ensembl.org/Emiliania_huxleyi/Info/Index) using HISAT2 [(version v2.1.0; parameter --dta --phred64 unstranded --new-summary -x index −1 read_r1 −2 read_r2 (PE)] (57). Then we aligned the clean reads to the reference genome sequence using Bowtie2 (version 2.2.5; parameter -q --phred64 --sensitive --dpad 0 --gbar 99999999 --mp 1,1np 1 --score-min L,0,–0.1 -p 16k 200) and calculated gene expression level using RSEM (version 1.2.8 with default parameter) (58, 59). The differential expressed gene detection in PA/P+ and (P+PA)/P+ comparisons was analyzed using the DESeq2 (60). False discovery rate (FDR) was used for the correction of the *P*-value to *q*-value (61, 62). The identification of DEGs was according to the following criteria: log_2_ fold change (FC) > 1 or < −1, and *q*-value <0.05 (63). GO and KEGG enrichment analysis of DEGs in PA/P+ and (P+PA)/P+ comparisons (*q*-value <0.05) were carried out using Phyper in R software.

### Statistical analysis

Experiments in this study were all performed in three biological replicates (*n* = 3) from which means and standard deviations were calculated. A one-way ANOVA was carried out using SPSS 16.0 (IBM, US) to assess the statistically significant differences in physiological responses of *E. huxleyi* between different groups at the level of *P* < 0.05. Post hoc tests were applied using Tukey’s honestly significant difference tests (equal variances) or Dunnett’s T3 (heterogeneous variances).

## Data Availability

The raw sequencing reads are available at NCBI Sequence Read Archive (SRA) database under the BioProject PRJNA904918.

## SUPPLEMENTAL MATERIAL

Supplemental figures and tables spectrum.01255-23-s0001.pdf Fig. S1 to S6, Tables S1 to S13.

ASM does not own the copyrights to Supplemental Material that may be linked to, or accessed through, an article. The authors have granted ASM a non-exclusive, world-wide license to publish the Supplemental Material files. Please contact the corresponding author directly for reuse.
