# Supplementary material for: Two-sided effects of the organic phosphorus phytate on a globally important marine coccolithophorid phytoplankton
Source: Microbiol Spectr. 2023 Sep 13;11(5):e01255-23. doi: 10.1128/spectrum.01255-23 (PMC10655706; doi:10.1128/spectrum.01255-23)
Supplement: Supplemental figures and tables — Fig. S1 to S6, Tables S1 to S13. [file spectrum.01255-23-s0001.pdf]

Two-sided effects of the ubiquitous organic phosphorus phytate on a globally important marine coccolithophorid phytoplankton

Jiashun Li, Kaidian Zhang, Ling Li, Yujie Wang, Senjie Lin*

The following supporting information is available for this article:

Fig. S1. Microscopic observations of *E. huxleyi* cells grown under different P conditions on the fifth day.

Fig. S2. Particulate inorganic carbon (PIC) content measured on the fifth day.

Fig. S3. Transcriptomic responses of *E. huxleyi* to PA addition.

Fig. S4. Expression of genes related to vacuole PA storage, poly-P synthesis, and fatty acid synthesis in PA/P+ and (P+PA)/P+ comparisons.

Fig. S5. RNA content of *E. huxleyi* under different conditions measured on the fifth day.

Fig. S6. Response of *E. huxleyi* to different DIP concentrations with phytate addition.

Table S1. Transcriptomic mapping statistics in *E. huxleyi*.

Table S2. Significantly differentially expressed genes (DEGs) in PA/P+ and (P+PA)/P+ comparisons.

Table S3. The significantly enriched KEGG pathways (q-value<0.05) of DEGs in the PA/P+ comparison.

Table S4. The KEGG pathways (q-value<0.05) significantly enriched by DEGs in the (P+PA)/P+ comparison.

Table S5. The information of DEGs involved in photosynthesis in PA/P+ and (P+PA)/P+ comparisons.

Table S6. The information of DEGs related to carbon fixation in Calvin cycle in PA/P+ and (P+PA)/P+ comparisons.

Table S7. DEGs involved in the biosynthesis of amino acid in PA/P+ and (P+PA)/P+ comparisons.

Table S8. DEGs involved in nitrogen metabolism in PA/P+ and (P+PA)/P+ comparisons.

Table S9. DEGs involved in ribosome biogenesis and ribosome in PA/P+ and (P+PA)/P+ comparisons.

Table S10. DEGs involved in glycolysis in PA/P+ and (P+PA)/P+ comparisons.

Table S11. DEGs involved in TCA cycle in PA/P+ and (P+PA)/P+ comparisons.

Table S12. DEGs involved in oxidative phosphorylation in PA/P+ and (P+PA)/P+ comparisons.

Table S13. DEGs involved in glycerophospholipid in PA/P+ and (P+PA)/P+ comparisons.

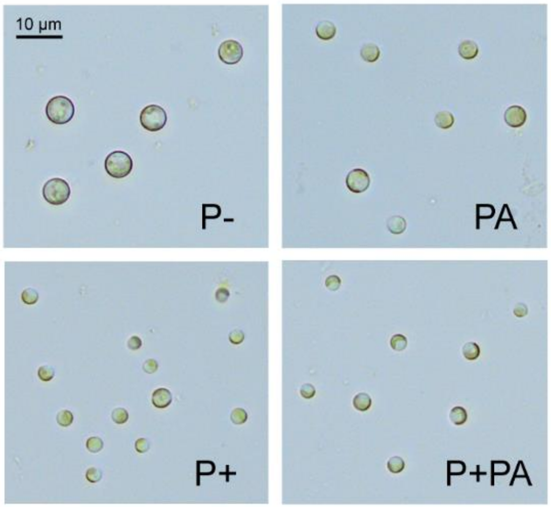

Fig. S1. Microscopic observations of *E. huxleyi* cells grown under different P conditions on the fifth day. Scale bar applies to all four images.

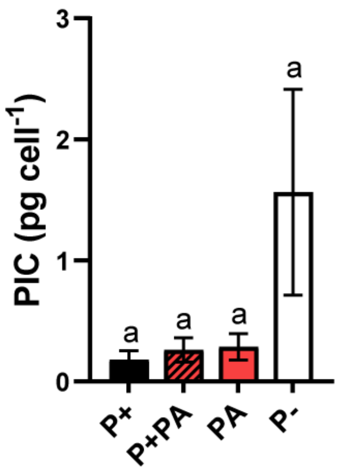

Fig. S2. Particulate inorganic carbon (PIC) content measured on the fifth day. Each data point is the mean of triplicate cultures with the error bar indicating standard deviation (Mean ± SD). Different letters above the columns indicate significant differences (ANOVA, $p < 0.05$).

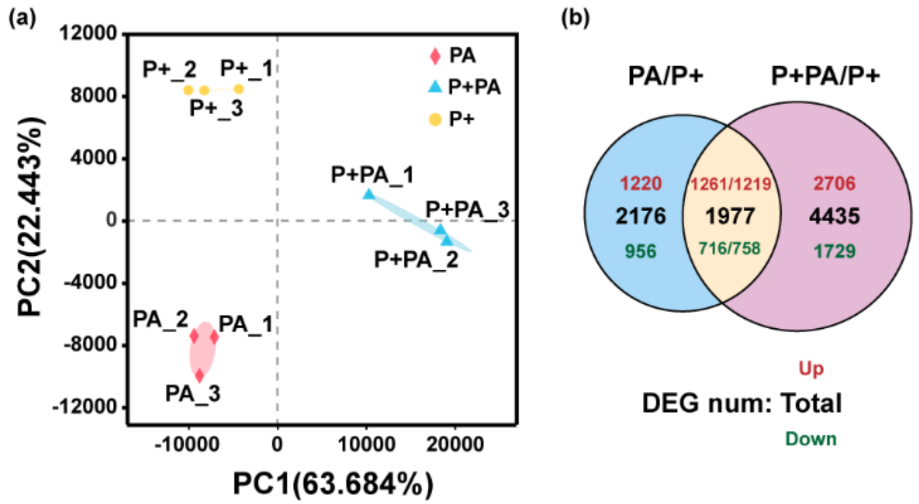

Fig. S3. Transcriptomic responses of *E. huxleyi* to PA addition. (a) Principal component analysis (PCA) of the RNA-Seq data of PA, P+, and P+PA groups. (b) Venn pie chart of DEGs in PA/P+ and (P+PA)/P+ comparisons. Numbers represent the number of DEGs.

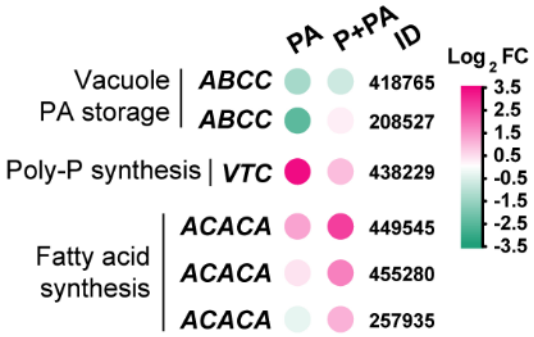

Fig. S4. Expression of genes related to vacuole PA storage, poly-P synthesis, and fatty acid synthesis in PA/P+ and (P+PA)/P+ comparisons. ABCC, ATP-binding cassette subfamily C; VTC, vacuolar transport chaperone; ACACA, acetyl-CoA carboxylase.

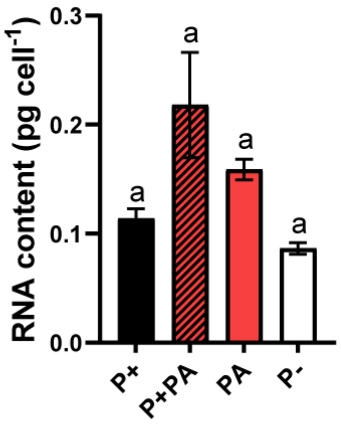

Fig. S5. RNA content of *E. huxleyi* under different P conditions measured on the fifth day. Each data point is the mean of triplicate cultures with the error bar indicating standard deviation (Mean ± SD). Different letters above the columns indicate significant differences (ANOVA, $p < 0.05$).

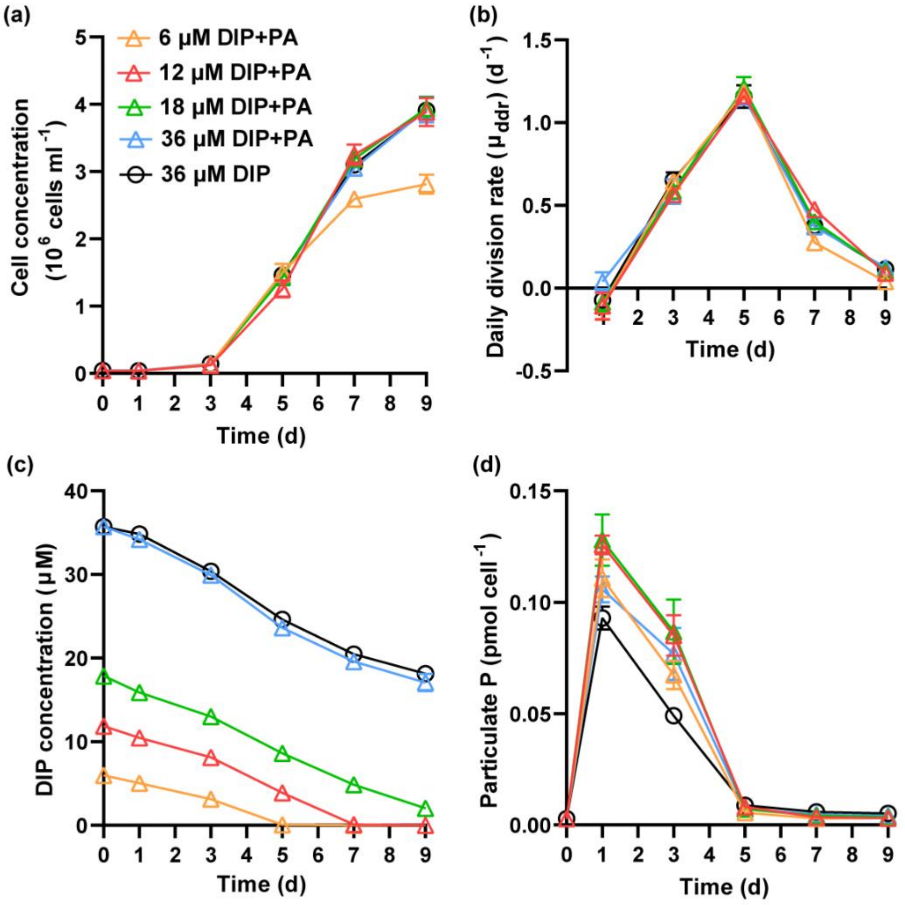

Fig. S6. Response of *E. huxleyi* to different DIP concentrations with phytate addition. (a) Cell concentration; (b) Daily growth rate; (c) DIP concentration in the medium; (d) Particulate P.

Table S1. Transcriptomic mapping statistics in *E. huxleyi*.

Group		Library	Total raw reads[a] (Million)	Total clean reads[b] (Million)	Clean reads ratio (%)	Total mapping (%)	Uniquely mapping ratio[c] (%)
Control group	P+	P+_1	43.82	42.63	97.28	73.37	36.04
		P+_2	43.82	42.29	96.50	72.85	35.66
		P+_3	43.82	42.45	96.86	73.30	34.89
PA-containing groups	PA	PA_1	43.82	42.65	97.34	72.94	35.50
		PA_2	43.82	42.53	97.06	72.81	35.35
		PA_3	43.82	42.28	96.48	72.80	34.63
	P+PA	P+PA_1	43.82	42.6	97.20	73.52	34.77
		P+PA_2	43.82	42.68	97.39	73.69	34.16
		P+PA_3	43.82	42.72	97.48	73.67	34.41

[a]The number of reads before quality filtering.

[b]The number of reads after quality filtering.

[c]The percentage of reads that were mapped to only one reference sequence.

Table S2. Significantly differentially expressed genes (DEGs) in PA/P+ and (P+PA)/P+ comparisons.

Comparison	Significantly differentially expressed genes (DEGs) (Log_2 FC[a] > 1, q-value[b] < 0.05)	Upregulated	Downregulated
PA/P+	4153	2481	1672
(P+PA)/P+	6412	3925	2487

[a]Log_2 fold change based on RNA-seq data.

[b]Equal to adjusted p-value, change is set at q-value < 0.05 in this study.

Table S3. The significantly enriched KEGG pathways (q-value<0.05) of DEGs in the PA/P+ comparison.

No.	Pathway ID	Pathway name	Term candidate gene number	Term gene number	Rich ratio	q-value[a]
1	ko03010	Ribosome	74	213	0.3474	4.01E-18
2	ko03008	Ribosome biogenesis in eukaryotes	47	186	0.2527	2.41E-06
3	ko00270	Cysteine and methionine metabolism	37	175	0.2114	3.22E-03
4	ko01230	Biosynthesis of amino acids	63	381	0.1654	2.29E-02
5	ko00710	Carbon fixation in photosynthetic organisms	21	95	0.2211	3.58E-02

[a]Equal to adjusted p-value, change is set at q-value < 0.05 in this study.

Table S4. The KEGG pathways (q-value<0.05) significantly enriched by DEGs in the (P+PA)/P+ comparison.

No.	Pathway ID	Pathway name	Term candidate gene number	Term gene number	Rich ratio	q-value[a]
1	ko03010	Ribosome	113	213	0.5305	1.65E-30
2	ko01230	Biosynthesis of amino acids	131	381	0.3438	3.04E-14
3	ko00710	Carbon fixation in photosynthetic organisms	44	95	0.4632	2.50E-09
4	ko03008	Ribosome biogenesis in eukaryotes	68	186	0.3656	8.40E-09
5	ko00196	Photosynthesis - antenna proteins	33	65	0.5077	1.88E-08
6	ko01200	Carbon metabolism	117	402	0.2910	6.34E-08
7	ko00010	Glycolysis / Gluconeogenesis	74	230	0.3217	5.15E-07
8	ko00620	Pyruvate metabolism	49	150	0.3267	5.80E-05
9	ko00190	Oxidative phosphorylation	45	137	0.3285	9.69E-05
10	ko00910	Nitrogen metabolism	24	56	0.4286	9.69E-05
11	ko00400	Phenylalanine, tyrosine and tryptophan biosynthesis	18	40	0.4500	5.25E-04
12	ko04145	Phagosome	33	100	0.3300	1.05E-03
13	ko00195	Photosynthesis	16	36	0.4444	1.30E-03
14	ko01212	Fatty acid metabolism	56	202	0.2772	1.33E-03
15	ko00061	Fatty acid biosynthesis	37	120	0.3083	1.65E-03
16	ko00250	Alanine, aspartate and glutamate metabolism	26	76	0.3421	2.14E-03
17	ko00270	Cysteine and methionine metabolism	49	175	0.2800	2.14E-03
18	ko00860	Porphyrin metabolism	30	95	0.3158	3.41E-03
19	ko00630	Glyoxylate and dicarboxylate metabolism	33	117	0.2821	1.45E-02
20	ko01240	Biosynthesis of cofactors	114	520	0.2192	2.26E-02
21	ko00230	Purine metabolism	51	209	0.2440	3.30E-02
22	ko00780	Biotin metabolism	22	75	0.2933	3.86E-02

[a]Equal to adjusted p-value, change is set at q-value < 0.05 in this study.

Table S5. The information of DEGs involved in photosynthesis in PA/P+ and (P+PA)/P+ comparisons.

No.	Gene ID	Gene name	KEGG orthology	PA/P+			(P+PA)/P+		
				Log_2 FC[a]	q-value[b]	Regulation	Log_2 FC	q-value	Regulation
1	EMIHUDRAFT_419362	LHC	K08907//light-harvesting complex I chlorophyll a/b binding protein 1+ko01100//Metabolic pathways+ko00196//Photosynthesis - antenna proteins;K08909//light-harvesting complex I chlorophyll a/b binding protein 3+ko01100//Metabolic pathways+ko00196//Photosynthesis - antenna proteins	1.3149	1.26E-02	Up	3.1244	1.30E-11	Up
2	EMIHUDRAFT_45605	LHC	K08907//light-harvesting complex I chlorophyll a/b binding protein 1+ko01100//Metabolic pathways+ko00196//Photosynthesis - antenna proteins	1.1673	1.59E-02	Up	3.1024	4.53E-12	Up
3	EMIHUDRAFT_45591	LHC	K08907//light-harvesting complex I chlorophyll a/b binding protein 1+ko01100//Metabolic pathways+ko00196//Photosynthesis - antenna proteins	0.9481	3.69E-01	-	3.0170	1.10E-08	Up
4	EMIHUDRAFT_312310	LHC	K08907//light-harvesting complex I chlorophyll a/b binding protein 1+ko01100//Metabolic pathways+ko00196//Photosynthesis - antenna proteins	0.9529	3.44E-02	-	2.9539	5.70E-13	Up
5	EMIHUDRAFT_417035	LHC	K08907//light-harvesting complex I chlorophyll a/b binding protein 1+ko01100//Metabolic pathways+ko00196//Photosynthesis - antenna proteins	0.9689	2.46E-02	-	2.9027	1.54E-12	Up
6	EMIHUDRAFT_444842	LHC	K08907//light-harvesting complex I chlorophyll a/b binding protein 1+ko01100//Metabolic pathways+ko00196//Photosynthesis - antenna proteins	1.7887	6.60E-06	Up	2.5909	2.93E-04	Up
7	EMIHUDRAFT_362550	LHC	K08907//light-harvesting complex I chlorophyll a/b binding protein 1+ko01100//Metabolic pathways+ko00196//Photosynthesis - antenna proteins	0.9121	2.53E-02	-	2.5882	4.53E-12	Up
8	BGI_novel_G001615	LHC	K08907//light-harvesting complex I chlorophyll a/b binding protein 1+ko01100//Metabolic pathways+ko00196//Photosynthesis - antenna proteins	1.4807	4.04E-04	Up	2.4938	6.99E-04	Up
9	EMIHUDRAFT_434108	LHC	K08907//light-harvesting complex I chlorophyll a/b binding protein 1+ko01100//Metabolic pathways+ko00196//Photosynthesis - antenna proteins	1.2841	1.11E-05	Up	2.4270	9.33E-15	Up
10	EMIHUDRAFT_70030	LHC	K08907//light-harvesting complex I chlorophyll a/b binding protein 1+ko01100//Metabolic pathways+ko00196//Photosynthesis - antenna proteins	0.7246	1.01E-01	-	2.4152	2.75E-09	Up
11	EMIHUDRAFT_419663	LHC	K08907//light-harvesting complex I chlorophyll a/b binding protein 1+ko01100//Metabolic pathways+ko00196//Photosynthesis - antenna proteins	0.7171	1.46E-01	-	2.3425	3.96E-08	Up

12	EMIHUDRAFT _413829	LHC	K08907//light-harvesting complex I chlorophyll a/b binding protein 1+ko01100//Metabolic pathways+ko00196//Photosynthesis - antenna proteins	0.3742	5.07E-01	-	2.3264	1.65E-09	Up
13	EMIHUDRAFT _211477	LHC	K08907//light-harvesting complex I chlorophyll a/b binding protein 1+ko01100//Metabolic pathways+ko00196//Photosynthesis - antenna proteins;K08910//light-harvesting complex I chlorophyll a/b binding protein 4+ko01100//Metabolic pathways+ko00196//Photosynthesis - antenna proteins	0.5365	6.12E-01	-	2.2896	1.09E-03	Up
14	EMIHUDRAFT _356951	LHC	K08911//light-harvesting complex I chlorophyll a/b binding protein 5+ko01100//Metabolic pathways+ko00196//Photosynthesis - antenna proteins;K08910//light-harvesting complex I chlorophyll a/b binding protein 4+ko01100//Metabolic pathways+ko00196//Photosynthesis - antenna proteins;K08915//light-harvesting complex II chlorophyll a/b binding protein 4+ko01100//Metabolic pathways+ko00196//Photosynthesis - antenna proteins;K08907//light-harvesting complex I chlorophyll a/b binding protein 1+ko01100//Metabolic pathways+ko00196//Photosynthesis - antenna proteins;K08908//light-harvesting complex I chlorophyll a/b binding protein 2+ko01100//Metabolic pathways+ko00196//Photosynthesis - antenna proteins	0.7514	1.89E-01	-	2.1943	3.93E-08	Up
15	EMIHUDRAFT _218313	LHC	K08907//light-harvesting complex I chlorophyll a/b binding protein 1+ko01100//Metabolic pathways+ko00196//Photosynthesis - antenna proteins;K08910//light-harvesting complex I chlorophyll a/b binding protein 4+ko01100//Metabolic pathways+ko00196//Photosynthesis - antenna proteins	0.2346	7.38E-01	-	2.0493	4.81E-08	Up
16	EMIHUDRAFT _439022	LHC	K08907//light-harvesting complex I chlorophyll a/b binding protein 1+ko01100//Metabolic pathways+ko00196//Photosynthesis - antenna proteins	0.6054	5.68E-02	-	1.9182	2.37E-11	Up
17	EMIHUDRAFT _461003	LHC	K08915//light-harvesting complex II chlorophyll a/b binding protein 4+ko01100//Metabolic pathways+ko00196//Photosynthesis - antenna proteins	0.2599	6.51E-01	-	1.8823	8.85E-08	Up
18	EMIHUDRAFT _447013	LHC	K08907//light-harvesting complex I chlorophyll a/b binding protein 1+ko01100//Metabolic pathways+ko00196//Photosynthesis - antenna proteins;K08910//light-harvesting complex I chlorophyll a/b binding protein 4+ko01100//Metabolic pathways+ko00196//Photosynthesis - antenna proteins	0.4481	7.37E-01	-	1.8424	2.18E-02	Up

19	EMIHUDRAFT_457448	LHC	K08907//light-harvesting complex I chlorophyll a/b binding protein 1+ko01100//Metabolic pathways+ko00196//Photosynthesis - antenna proteins	0.4322	4.16E-01	-	1.7533	3.36E-06	Up
20	EMIHUDRAFT_452954	LHC	K08907//light-harvesting complex I chlorophyll a/b binding protein 1+ko01100//Metabolic pathways+ko00196//Photosynthesis - antenna proteins	0.3042	6.33E-01	-	1.7350	8.68E-06	Up
21	EMIHUDRAFT_67603	LHC	K08907//light-harvesting complex I chlorophyll a/b binding protein 1+ko01100//Metabolic pathways+ko00196//Photosynthesis - antenna proteins	-0.6429	1.58E-01	-	1.6627	2.43E-05	Up
22	EMIHUDRAFT_74777	LHC	K08907//light-harvesting complex I chlorophyll a/b binding protein 1+ko01100//Metabolic pathways+ko00196//Photosynthesis - antenna proteins; K08910//light-harvesting complex I chlorophyll a/b binding protein 4+ko01100//Metabolic pathways+ko00196//Photosynthesis - antenna proteins	-0.6429	1.58E-01	-	1.6627	2.43E-05	Up
23	EMIHUDRAFT_353537	LHC	K08907//light-harvesting complex I chlorophyll a/b binding protein 1+ko01100//Metabolic pathways+ko00196//Photosynthesis - antenna proteins;K08910//light-harvesting complex I chlorophyll a/b binding protein 4+ko01100//Metabolic pathways+ko00196//Photosynthesis - antenna proteins	-0.2395	7.03E-01	-	1.6431	8.77E-06	Up
24	EMIHUDRAFT_438393	LHC	K08907//light-harvesting complex I chlorophyll a/b binding protein 1+ko01100//Metabolic pathways+ko00196//Photosynthesis - antenna proteins	0.3608	2.72E-01	-	1.6394	5.96E-10	Up
25	EMIHUDRAFT_445064	LHC	K08907//light-harvesting complex I chlorophyll a/b binding protein 1+ko01100//Metabolic pathways+ko00196//Photosynthesis - antenna proteins	0.3608	2.72E-01	-	1.6394	5.96E-10	Up
26	EMIHUDRAFT_442651	LHC	K08907//light-harvesting complex I chlorophyll a/b binding protein 1+ko01100//Metabolic pathways+ko00196//Photosynthesis - antenna proteins;K08911//light-harvesting complex I chlorophyll a/b binding protein 5+ko01100//Metabolic pathways+ko00196//Photosynthesis - antenna proteins;K08908//light-harvesting complex I chlorophyll a/b binding protein 2+ko01100//Metabolic pathways+ko00196//Photosynthesis - antenna proteins	-0.9239	1.17E-02	-	1.4053	1.15E-04	Up
27	EMIHUDRAFT_233788	LHC	K08907//light-harvesting complex I chlorophyll a/b binding protein 1+ko01100//Metabolic pathways+ko00196//Photosynthesis - antenna proteins	-0.5095	1.62E-01	-	1.2307	6.27E-09	Up
28	EMIHUDRAFT_236822	LHC	K08907//light-harvesting complex I chlorophyll a/b binding protein 1+ko01100//Metabolic pathways+ko00196//Photosynthesis - antenna proteins	-0.3299	4.39E-01	-	1.2040	4.07E-07	Up

29	EMIHUDRAFT _441864	LHC	K08907//light-harvesting complex I chlorophyll a/b binding protein 1+ko01100//Metabolic pathways+ko00196//Photosynthesis - antenna proteins;K08911//light-harvesting complex I chlorophyll a/b binding protein 5+ko01100//Metabolic pathways+ko00196//Photosynthesis - antenna proteins;K08908//light-harvesting complex I chlorophyll a/b binding protein 2+ko01100//Metabolic pathways+ko00196//Photosynthesis - antenna proteins	-0.5957	1.90E-01	-	1.1667	1.72E-03	Up
30	EMIHUDRAFT _443721	LHC	K08907//light-harvesting complex I chlorophyll a/b binding protein 1+ko01100//Metabolic pathways+ko00196//Photosynthesis - antenna proteins	1.0612	1.68E-03	Up	0.7793	4.17E-02	-
31	EMIHUDRAFT _435996	LHC	K08907//light-harvesting complex I chlorophyll a/b binding protein 1+ko01100//Metabolic pathways+ko00196//Photosynthesis - antenna proteins	1.2590	7.37E-07	Up	-0.3082	3.98E-01	-
32	EMIHUDRAFT _442232	LHC	K08907//light-harvesting complex I chlorophyll a/b binding protein 1+ko01100//Metabolic pathways+ko00196//Photosynthesis - antenna proteins	1.0123	1.46E-03	Up	-0.3256	4.76E-01	-
33	EMIHUDRAFT _416733	LHC	K08907//light-harvesting complex I chlorophyll a/b binding protein 1+ko01100//Metabolic pathways+ko00196//Photosynthesis - antenna proteins;K08910//light-harvesting complex I chlorophyll a/b binding protein 4+ko01100//Metabolic pathways+ko00196//Photosynthesis - antenna proteins	-1.1052	1.48E-04	Down	-0.9127	4.96E-03	-
34	EMIHUDRAFT _76288	LHC	K08907//light-harvesting complex I chlorophyll a/b binding protein 1+ko01100//Metabolic pathways+ko00196//Photosynthesis - antenna proteins	-2.5124	7.51E-07	Down	-1.2128	2.83E-02	Down
35	EMIHUDRAFT _224646	LHC	K08907//light-harvesting complex I chlorophyll a/b binding protein 1+ko01100//Metabolic pathways+ko00196//Photosynthesis - antenna proteins;K08915//light-harvesting complex II chlorophyll a/b binding protein 4+ko01100//Metabolic pathways+ko00196//Photosynthesis - antenna proteins	-2.6084	4.20E-13	Down	-1.2384	2.90E-03	Down
36	EMIHUDRAFT _467343	LHC	K08907//light-harvesting complex I chlorophyll a/b binding protein 1+ko01100//Metabolic pathways+ko00196//Photosynthesis - antenna proteins	-1.8196	1.88E-06	Down	-1.3948	3.01E-04	Down
37	EMIHUDRAFT _45662	LHC	K08907//light-harvesting complex I chlorophyll a/b binding protein 1+ko01100//Metabolic pathways+ko00196//Photosynthesis - antenna proteins	-2.2983	8.64E-11	Down	-1.6107	2.68E-05	Down
38	EMIHUDRAFT _451739	LHC	K08907//light-harvesting complex I chlorophyll a/b binding protein 1+ko01100//Metabolic pathways+ko00196//Photosynthesis - antenna proteins;K08908//light-harvesting complex I chlorophyll a/b	-1.1114	5.22E-03	Down	0.0825	9.02E-01	-

			binding protein 2+ko01100//Metabolic pathways+ko00196//Photosynthesis - antenna proteins;K08911//light-harvesting complex I chlorophyll a/b binding protein 5+ko01100//Metabolic pathways+ko00196//Photosynthesis - antenna proteins						
39	EMIHUDRAFT_364696	LHC	K08907//light-harvesting complex I chlorophyll a/b binding protein 1+ko01100//Metabolic pathways+ko00196//Photosynthesis - antenna proteins;K08915//light-harvesting complex II chlorophyll a/b binding protein 4+ko01100//Metabolic pathways+ko00196//Photosynthesis - antenna proteins	-1.2318	9.90E-04	Down	0.1027	8.63E-01	-
40	EMIHUDRAFT_66718	PetF	K02639//ferredoxin+ko01100//Metabolic pathways+ko00195//Photosynthesis	1.5670	1.74E-01	-	3.0607	4.84E-04	Up
41	EMIHUDRAFT_428354	PsbU	K02719//photosystem II PsbU protein+ko01100//Metabolic pathways+ko00195//Photosynthesis	0.6927	1.41E-01	-	2.2195	3.44E-08	Up
42	EMIHUDRAFT_419966	PsbU	K02719//photosystem II PsbU protein+ko01100//Metabolic pathways+ko00195//Photosynthesis	0.6927	1.41E-01	-	2.2195	3.44E-08	Up
43	EMIHUDRAFT_59827	PetF	K02639//ferredoxin+ko01100//Metabolic pathways+ko00195//Photosynthesis	1.4451	1.40E-01	-	2.0020	1.14E-02	Up
44	EMIHUDRAFT_432385	PetH	K02641//ferredoxin--NADP+ reductase [EC:1.18.1.2]+ko01100//Metabolic pathways+ko00195//Photosynthesis;K02641//ferredoxin--NADP+ reductase [EC:1.18.1.2]+ko01100//Metabolic pathways+ko00195//Photosynthesis	0.8333	3.64E-01	-	1.9931	7.11E-03	Up
45	EMIHUDRAFT_361737	PetH	K02641//ferredoxin--NADP+ reductase [EC:1.18.1.2]+ko01100//Metabolic pathways+ko00195//Photosynthesis	0.5307	2.43E-01	-	1.9900	1.55E-08	Up
46	EMIHUDRAFT_463191	PsbS	K03542//photosystem II 22kDa protein+ko01100//Metabolic pathways+ko00195//Photosynthesis	0.4041	3.68E-01	-	1.9016	9.22E-11	Up
47	EMIHUDRAFT_49896	PsaE	K02693//photosystem I subunit IV+ko01100//Metabolic pathways+ko00195//Photosynthesis;K02693//photosystem I subunit IV+ko01100//Metabolic pathways+ko00195//Photosynthesis	0.2988	6.72E-01	-	1.8245	1.84E-06	Up
48	EMIHUDRAFT_75661	PetF	K02639//ferredoxin+ko01100//Metabolic pathways+ko00195//Photosynthesis	0.4040	4.37E-01	-	1.7498	1.31E-05	Up
49	EMIHUDRAFT_106967	PsbS	K03542//photosystem II 22kDa protein+ko01100//Metabolic pathways+ko00195//Photosynthesis	0.6628	5.60E-02	-	1.7136	3.63E-09	Up
50	EMIHUDRAFT_449561	PsaE	K02693//photosystem I subunit IV+ko01100//Metabolic pathways+ko00195//Photosynthesis	0.3225	6.14E-01	-	1.4728	2.76E-04	Up
51	EMIHUDRAFT_438165	PsbO	K02716//photosystem II oxygen-evolving enhancer protein 1+ko01100//Metabolic pathways+ko00195//Photosynthesis	0.5533	1.76E-01	-	1.3856	9.32E-05	Up

52	EMIHUDRAFT _45312	PetC	K02636//cytochrome b6-f complex iron-sulfur subunit [EC:7.1.1.6]+ko01100//Metabolic pathways+ko00195//Photosynthesis	0.5024	1.48E-01	-	1.3243	9.26E-07	Up
53	EMIHUDRAFT _461699	F-type ATPase gamma	K02115//F-type H+-transporting ATPase subunit gamma+ko01100//Metabolic pathways+ko00190//Oxidative phosphorylation+ko00195//Photosynthesis	0.9164	2.18E-03	-	1.3061	5.30E-06	Up
54	EMIHUDRAFT _417113	PetE	K02638//plastocyanin+ko01100//Metabolic pathways+ko00195//Photosynthesis	0.2941	5.30E-01	-	1.2875	5.82E-05	Up
55	EMIHUDRAFT _440922	PsbO	K02716//photosystem II oxygen-evolving enhancer protein 1+ko01100//Metabolic pathways+ko00195//Photosynthesis	0.3522	5.45E-01	-	1.1712	4.00E-03	Up

[a]Log_2 fold change based on RNA-seq data. Each value is the mean from three biological replicates.

[b]Equal to adjusted p-value, change is set at q-value < 0.05 in this study.

Table S6. The information of DEGs related to carbon fixation in Calvin cycle in PA/P+ and (P+PA)/P+ comparisons.

No.	Gene ID	Gene name	KEGG orthology	PA/P+			(P+PA)/P+		
				Log_2 FC[a]	q-value[b]	Regulation	Log_2 FC	q-value	Regulation
1	EMIHUDRAFT_365175	PGK	K00927//phosphoglycerate kinase [EC:2.7.2.3]+ko00010//Glycolysis / Gluconeogenesis+ko01230//Biosynthesis of amino acids+ko01100//Metabolic pathways+ko01110//Biosynthesis of secondary metabolites+ko00710//Carbon fixation in photosynthetic organisms+ko01200//Carbon metabolism	1.5686	1.73E-05	Up	4.4336	5.26E-32	Up
2	EMIHUDRAFT_63832	PGK	K00927//phosphoglycerate kinase [EC:2.7.2.3]+ko00010//Glycolysis / Gluconeogenesis+ko01230//Biosynthesis of amino acids+ko01100//Metabolic pathways+ko01110//Biosynthesis of secondary metabolites+ko00710//Carbon fixation in photosynthetic organisms+ko01200//Carbon metabolism	1.0855	1.30E-02	Up	2.2345	7.86E-12	Up
3	EMIHUDRAFT_417537	PGK	K00927//phosphoglycerate kinase [EC:2.7.2.3]+ko00010//Glycolysis / Gluconeogenesis+ko01230//Biosynthesis of amino acids+ko01100//Metabolic pathways+ko01110//Biosynthesis of secondary metabolites+ko00710//Carbon fixation in photosynthetic organisms+ko01200//Carbon metabolism	0.9794	9.39E-03	-	3.3104	8.58E-18	Up
4	EMIHUDRAFT_99818	PGK	K00927//phosphoglycerate kinase [EC:2.7.2.3]+ko00010//Glycolysis / Gluconeogenesis+ko01230//Biosynthesis of amino acids+ko01100//Metabolic pathways+ko01110//Biosynthesis of secondary metabolites+ko00710//Carbon fixation in photosynthetic organisms+ko01200//Carbon metabolism	1.3566	7.46E-04	Up	2.2278	7.21E-11	Up
5	EMIHUDRAFT_72672	PGK	K00927//phosphoglycerate kinase [EC:2.7.2.3]+ko00010//Glycolysis / Gluconeogenesis+ko01230//Biosynthesis of amino acids+ko01100//Metabolic pathways+ko01110//Biosynthesis of secondary metabolites+ko00710//Carbon fixation in photosynthetic organisms+ko01200//Carbon metabolism	1.9975	6.05E-03	Up	0.9936	3.86E-01	-
6	EMIHUDRAFT_107385	PGK	K00927//phosphoglycerate kinase [EC:2.7.2.3]+ko00010//Glycolysis / Gluconeogenesis+ko01230//Biosynthesis of amino acids+ko01100//Metabolic pathways+ko01110//Biosynthesis of secondary metabolites+ko00710//Carbon fixation in photosynthetic organisms+ko01200//Carbon metabolism	1.9893	5.57E-03	Up	0.4539	7.18E-01	-
7	EMIHUDRAFT_44530	GAPDH	K00134//glyceraldehyde 3-phosphate dehydrogenase (phosphorylating) [EC:1.2.1.12]+ko00010//Glycolysis / Gluconeogenesis+ko01230//Biosynthesis of amino acids+ko01100//Metabolic pathways+ko01110//Biosynthesis of secondary metabolites+ko00710//Carbon fixation in photosynthetic organisms+ko01200//Carbon metabolism	1.3987	4.67E-02	Up	3.8167	1.23E-16	Up

8	EMIHUDRAFT_216734	GAPDH	K00134//glyceraldehyde 3-phosphate dehydrogenase (phosphorylating) [EC:1.2.1.12]+ko00010//Glycolysis / Gluconeogenesis+ko01230//Biosynthesis of amino acids+ko01100//Metabolic pathways+ko01110//Biosynthesis of secondary metabolites+ko00710//Carbon fixation in photosynthetic organisms+ko01200//Carbon metabolism	1.4152	1.79E-09	Up	-0.3552	3.31E-01	-
9	EMIHUDRAFT_437834	GAPDH	K00134//glyceraldehyde 3-phosphate dehydrogenase (phosphorylating) [EC:1.2.1.12]+ko00010//Glycolysis / Gluconeogenesis+ko01230//Biosynthesis of amino acids+ko01100//Metabolic pathways+ko01110//Biosynthesis of secondary metabolites+ko00710//Carbon fixation in photosynthetic organisms+ko01200//Carbon metabolism	1.3667	7.60E-02	-	3.6428	3.91E-07	Up
10	EMIHUDRAFT_421124	GAPDH	K00134//glyceraldehyde 3-phosphate dehydrogenase (phosphorylating) [EC:1.2.1.12]+ko00010//Glycolysis / Gluconeogenesis+ko01230//Biosynthesis of amino acids+ko01100//Metabolic pathways+ko01110//Biosynthesis of secondary metabolites+ko00710//Carbon fixation in photosynthetic organisms+ko01200//Carbon metabolism	1.1677	1.16E-01	-	3.0873	4.21E-06	Up
11	BGI_novel_G004845	GAPDH	K00134//glyceraldehyde 3-phosphate dehydrogenase (phosphorylating) [EC:1.2.1.12]+ko00010//Glycolysis / Gluconeogenesis+ko01230//Biosynthesis of amino acids+ko01100//Metabolic pathways+ko01110//Biosynthesis of secondary metabolites+ko00710//Carbon fixation in photosynthetic organisms+ko01200//Carbon metabolism	0.9553	4.10E-01	-	2.0717	1.03E-02	Up
12	EMIHUDRAFT_365287	GAPDH	K05298//glyceraldehyde-3-phosphate dehydrogenase (NADP+) (phosphorylating) [EC:1.2.1.13]+ko01100//Metabolic pathways+ko00710//Carbon fixation in photosynthetic organisms+ko01200//Carbon metabolism;K00134//glyceraldehyde 3-phosphate dehydrogenase (phosphorylating) [EC:1.2.1.12]+ko00010//Glycolysis / Gluconeogenesis+ko01230//Biosynthesis of amino acids+ko01100//Metabolic pathways+ko01110//Biosynthesis of secondary metabolites+ko00710//Carbon fixation in photosynthetic organisms+ko01200//Carbon metabolism	1.4398	1.37E-10	Up	1.5944	4.62E-09	Up
13	BGI_novel_G000759	GAPDH	K00134//glyceraldehyde 3-phosphate dehydrogenase (phosphorylating) [EC:1.2.1.12]+ko00010//Glycolysis / Gluconeogenesis+ko01230//Biosynthesis of amino acids+ko01100//Metabolic pathways+ko01110//Biosynthesis of secondary metabolites+ko00710//Carbon fixation in photosynthetic organisms+ko01200//Carbon metabolism	-0.3217	7.18E-01	-	1.2146	1.40E-02	Up
14	EMIHUDRA	FBA	K01623//fructose-bisphosphate aldolase, class I [EC:4.1.2.13]+ko00010//Glycolysis / Gluconeogenesis+ko00030//Pentose phosphate	1.2062	2.58E-01	-	4.7148	1.55E-18	Up

	FT_308857		pathway+ko01230//Biosynthesis of amino acids+ko01100//Metabolic pathways+ko00051//Fructose and mannose metabolism+ko01110//Biosynthesis of secondary metabolites+ko00710//Carbon fixation in photosynthetic organisms+ko01200//Carbon metabolism						
15	EMIHUDRAFT_436550	FBA	K01624//fructose-bisphosphate aldolase, class II [EC:4.1.2.13]+ko00010//Glycolysis / Gluconeogenesis+ko00030//Pentose phosphate pathway+ko01230//Biosynthesis of amino acids+ko01100//Metabolic pathways+ko00051//Fructose and mannose metabolism+ko01110//Biosynthesis of secondary metabolites+ko00710//Carbon fixation in photosynthetic organisms+ko01200//Carbon metabolism	1.1558	5.41E-03	Up	2.0204	2.42E-08	Up
16	EMIHUDRAFT_418341	FBA	K01624//fructose-bisphosphate aldolase, class II [EC:4.1.2.13]+ko00010//Glycolysis / Gluconeogenesis+ko00030//Pentose phosphate pathway+ko01230//Biosynthesis of amino acids+ko01100//Metabolic pathways+ko00051//Fructose and mannose metabolism+ko01110//Biosynthesis of secondary metabolites+ko00710//Carbon fixation in photosynthetic organisms+ko01200//Carbon metabolism	0.2923	4.65E-01	-	1.3778	4.71E-08	Up
17	EMIHUDRAFT_434018	FBP	K03841//fructose-1,6-bisphosphatase I [EC:3.1.3.11]+ko00010//Glycolysis / Gluconeogenesis+ko00030//Pentose phosphate pathway+ko01100//Metabolic pathways+ko00051//Fructose and mannose metabolism+ko01110//Biosynthesis of secondary metabolites+ko00710//Carbon fixation in photosynthetic organisms+ko01200//Carbon metabolism	0.5514	1.26E-01	-	2.3607	1.57E-15	Up
18	EMIHUDRAFT_101315	FBP	K03841//fructose-1,6-bisphosphatase I [EC:3.1.3.11]+ko00010//Glycolysis / Gluconeogenesis+ko00030//Pentose phosphate pathway+ko01100//Metabolic pathways+ko00051//Fructose and mannose metabolism+ko01110//Biosynthesis of secondary metabolites+ko00710//Carbon fixation in photosynthetic organisms+ko01200//Carbon metabolism	0.3912	5.58E-01	-	-1.3877	2.11E-03	Down
19	EMIHUDRAFT_437959	TKT	K00615//transketolase [EC:2.2.1.1]+ko00030//Pentose phosphate pathway+ko01230//Biosynthesis of amino acids+ko01100//Metabolic pathways+ko01110//Biosynthesis of secondary metabolites+ko00710//Carbon fixation in photosynthetic organisms+ko01200//Carbon metabolism	1.3147	8.15E-02		2.4221	3.68E-10	Up
20	EMIHUDRA	TKT	K00615//transketolase [EC:2.2.1.1]+ko00030//Pentose phosphate pathway+ko01230//Biosynthesis of amino acids+ko01100//Metabolic pathways+ko01110//Biosynthesis of secondary	1.8505	1.25E-02	Up	-1.1565	4.71E-02	Down

	FT_45 8791		metabolites+ko00710//Carbon fixation in photosynthetic organisms+ko01200//Carbon metabolism						
21	EMIH UDRA FT_44 2242	SBP	K01100//sedoheptulose-bisphosphatase [EC:3.1.3.37]+ko01100//Metabolic pathways+ko00710//Carbon fixation in photosynthetic organisms+ko01200//Carbon metabolism	1.3246	2.09E-02	Up	1.6532	2.57E-04	Up
22	EMIH UDRA FT_44 6966	SBP	K01100//sedoheptulose-bisphosphatase [EC:3.1.3.37]+ko01100//Metabolic pathways+ko00710//Carbon fixation in photosynthetic organisms+ko01200//Carbon metabolism	1.0858	3.40E-03	Up	1.3249	1.35E-05	Up
23	EMIH UDRA FT_46 5735	SBP	K01100//sedoheptulose-bisphosphatase [EC:3.1.3.37]+ko01100//Metabolic pathways+ko00710//Carbon fixation in photosynthetic organisms+ko01200//Carbon metabolism	- 0.1983	7.53E-01	-	1.3043	4.33E-07	Up
24	EMIH UDRA FT_35 1398	RPI	K01807//ribose 5-phosphate isomerase A [EC:5.3.1.6]+ko00030//Pentose phosphate pathway+ko01230//Biosynthesis of amino acids+ko01100//Metabolic pathways+ko01110//Biosynthesis of secondary metabolites+ko00710//Carbon fixation in photosynthetic organisms+ko01200//Carbon metabolism	0.4445	3.93E-01	-	1.6624	4.11E-06	Up
25	EMIH UDRA FT_73 582	RPI	K00852//ribokinase [EC:2.7.1.15]+ko00030//Pentose phosphate pathway+ko01100//Metabolic pathways;K01807//ribose 5-phosphate isomerase A [EC:5.3.1.6]+ko00030//Pentose phosphate pathway+ko01230//Biosynthesis of amino acids+ko01100//Metabolic pathways+ko01110//Biosynthesis of secondary metabolites+ko00710//Carbon fixation in photosynthetic organisms+ko01200//Carbon metabolism	0.3124	4.63E-01	-	- 1.1658	4.44E-05	Down
26	EMIH UDRA FT_77 774	RPI	K00852//ribokinase [EC:2.7.1.15]+ko00030//Pentose phosphate pathway+ko01100//Metabolic pathways;K01807//ribose 5-phosphate isomerase A [EC:5.3.1.6]+ko00030//Pentose phosphate pathway+ko01230//Biosynthesis of amino acids+ko01100//Metabolic pathways+ko01110//Biosynthesis of secondary metabolites+ko00710//Carbon fixation in photosynthetic organisms+ko01200//Carbon metabolism	0.3141	4.47E-01	-	- 1.1938	9.07E-07	Down
27	EMIH UDRA FT_43 2636	PRK	K00855//phosphoribulokinase [EC:2.7.1.19]+ko01100//Metabolic pathways+ko00710//Carbon fixation in photosynthetic organisms+ko01200//Carbon metabolism	0.8203	3.10E-01	-	2.2736	5.88E-09	Up

[a]Log_2 fold change based on RNA-seq data. Each value is the mean from three biological replicates.

[b]Equal to adjusted p-value, change is set at q-value < 0.05 in this study.

Table S7. DEGs involved in the biosynthesis of amino acid in PA/P+ and (P+PA)/P+ comparisons.

No.	Gene ID	KEGG orthology	PA/P+			(P+PA)/P+		
			Log_2 FC[a]	q-value[b]	Regulation	Log_2 FC[a]	q-value[b]	Regulation
1	BGI_novel_G003843	K01915//glutamine synthetase [EC:6.3.1.2]+ko00250//Alanine, aspartate and glutamate metabolism+ko00910//Nitrogen metabolism+ko00220//Arginine biosynthesis+ko01230//Biosynthesis of amino acids+ko01100//Metabolic pathways+ko00630//Glyoxylate and dicarboxylate metabolism	2.2982	2.81E-03	Up	4.7596	5.58E-18	Up
2	EMIHUDRAFT_452329	K01733//threonine synthase [EC:4.2.3.1]+ko00750//Vitamin B6 metabolism+ko01230//Biosynthesis of amino acids+ko01100//Metabolic pathways+ko01110//Biosynthesis of secondary metabolites+ko00260//Glycine, serine and threonine metabolism;K13171//serine/arginine repetitive matrix protein 1+ko03015//mRNA surveillance pathway+ko03013//Nucleocytoplasmic transport	2.2740	1.20E-03	Up	4.2866	4.67E-20	Up
3	EMIHUDRAFT_62327	K00013//histidinol dehydrogenase [EC:1.1.1.23]+ko01230//Biosynthesis of amino acids+ko01100//Metabolic pathways+ko00340//Histidine metabolism+ko01110//Biosynthesis of secondary metabolites;K00765//ATP phosphoribosyltransferase [EC:2.4.2.17]+ko01230//Biosynthesis of amino acids+ko01100//Metabolic pathways+ko00340//Histidine metabolism+ko01110//Biosynthesis of secondary metabolites;K00013//histidinol dehydrogenase [EC:1.1.1.23]+ko01230//Biosynthesis of amino acids+ko01100//Metabolic pathways+ko00340//Histidine metabolism+ko01110//Biosynthesis of secondary metabolites	2.7767	1.97E-08	Up	4.1694	3.38E-09	Up
4	EMIHUDRAFT_468689	K00052//3-isopropylmalate dehydrogenase [EC:1.1.1.85]+ko00290//Valine, leucine and isoleucine biosynthesis+ko01230//Biosynthesis of amino acids+ko01100//Metabolic pathways+ko01110//Biosynthesis of secondary metabolites+ko01210//2-Oxocarboxylic acid metabolism+ko00660//C5-Branched dibasic acid metabolism;K00052//3-isopropylmalate dehydrogenase [EC:1.1.1.85]+ko00290//Valine, leucine and isoleucine biosynthesis+ko01230//Biosynthesis of amino acids+ko01100//Metabolic pathways+ko01110//Biosynthesis of secondary metabolites+ko01210//2-Oxocarboxylic acid metabolism+ko00660//C5-Branched dibasic acid metabolism	2.5557	2.26E-08	Up	4.0903	2.11E-25	Up
5	EMIHUDRAFT_456474	K01755//argininosuccinate lyase [EC:4.3.2.1]+ko00250//Alanine, aspartate and glutamate metabolism+ko00220//Arginine biosynthesis+ko01230//Biosynthesis of amino acids+ko01100//Metabolic pathways+ko01110//Biosynthesis of secondary metabolites;K01755//argininosuccinate lyase [EC:4.3.2.1]+ko00250//Alanine, aspartate and glutamate metabolism+ko00220//Arginine biosynthesis+ko01230//Biosynthesis of amino acids+ko01100//Metabolic pathways+ko01110//Biosynthesis of secondary metabolites	2.2593	2.91E-06	Up	4.0487	8.81E-20	Up
6	EMIHUDRA	K00053//ketol-acid reductoisomerase [EC:1.1.1.86]+ko00290//Valine, leucine and isoleucine biosynthesis+ko00770//Pantothenate and CoA	1.7003	2.76E-04	Up	3.6991	4.84E-08	Up

	FT_103401	biosynthesis+ko01230//Biosynthesis of amino acids+ko01100//Metabolic pathways+ko01110//Biosynthesis of secondary metabolites+ko01210//2-Oxocarboxylic acid metabolism						
7	EMIHUDRAFT_454560	K01754//threonine dehydratase [EC:4.3.1.19]+ko00290//Valine, leucine and isoleucine biosynthesis+ko01230//Biosynthesis of amino acids+ko01100//Metabolic pathways+ko01110//Biosynthesis of secondary metabolites+ko00260//Glycine, serine and threonine metabolism+ko01200//Carbon metabolism	1.8656	7.32E-04	Up	3.5964	6.54E-16	Up
8	EMIHUDRAFT_198673	K00789//S-adenosylmethionine synthetase [EC:2.5.1.6]+ko00999//Biosynthesis of various plant secondary metabolites+ko01240//Biosynthesis of cofactors+ko01230//Biosynthesis of amino acids+ko01100//Metabolic pathways+ko01110//Biosynthesis of secondary metabolites+ko00270//Cysteine and methionine metabolism	1.6535	2.51E-05	Up	3.4966	4.01E-19	Up
9	EMIHUDRAFT_66467	K01953//asparagine synthase (glutamine-hydrolysing) [EC:6.3.5.4]+ko00250//Alanine, aspartate and glutamate metabolism+ko01230//Biosynthesis of amino acids+ko01100//Metabolic pathways+ko01110//Biosynthesis of secondary metabolites	1.1108	1.10E-03	Up	2.0789	2.78E-13	Up
10	EMIHUDRAFT_438904	K01586//diaminopimelate decarboxylase [EC:4.1.1.20]+ko00300//Lysine biosynthesis+ko01230//Biosynthesis of amino acids+ko01100//Metabolic pathways+ko01110//Biosynthesis of secondary metabolites+ko00470//D-Amino acid metabolism	1.1641	1.11E-04	Up	1.7143	1.56E-09	Up
11	EMIHUDRAFT_415674	K00058//D-3-phosphoglycerate dehydrogenase / 2-oxoglutarate reductase [EC:1.1.1.95 1.1.1.399]+ko01230//Biosynthesis of amino acids+ko01100//Metabolic pathways+ko01110//Biosynthesis of secondary metabolites+ko00260//Glycine, serine and threonine metabolism+ko00270//Cysteine and methionine metabolism+ko01200//Carbon metabolism	1.2137	2.99E-02	Up	3.4418	4.71E-23	Up
12	EMIHUDRAFT_453895	K01652//acetolactate synthase I/II/III large subunit [EC:2.2.1.6]+ko00290//Valine, leucine and isoleucine biosynthesis+ko00770//Pantothenate and CoA biosynthesis+ko01230//Biosynthesis of amino acids+ko01100//Metabolic pathways+ko00650//Butanoate metabolism+ko01110//Biosynthesis of secondary metabolites+ko01210//2-Oxocarboxylic acid metabolism+ko00660//C5-Branched dibasic acid metabolism	1.1307	1.38E-05	Up	2.1193	2.83E-15	Up
13	EMIHUDRAFT_437187	K01915//glutamine synthetase [EC:6.3.1.2]+ko00250//Alanine, aspartate and glutamate metabolism+ko00910//Nitrogen metabolism+ko00220//Arginine biosynthesis+ko01230//Biosynthesis of amino acids+ko01100//Metabolic pathways+ko00630//Glyoxylate and dicarboxylate metabolism;K01915//glutamine synthetase [EC:6.3.1.2]+ko00250//Alanine, aspartate and glutamate metabolism+ko00910//Nitrogen metabolism+ko00220//Arginine biosynthesis+ko01230//Biosynthesis of amino acids+ko01100//Metabolic pathways+ko00630//Glyoxylate and dicarboxylate metabolism	1.7321	2.42E-02	Up	3.3299	7.08E-07	Up
14	EMIHUDRA	K00058//D-3-phosphoglycerate dehydrogenase / 2-oxoglutarate reductase [EC:1.1.1.95 1.1.1.399]+ko01230//Biosynthesis of amino	1.2670	6.60E-03	Up	3.3297	6.06E-17	Up

	FT_465676	acids+ko01100//Metabolic pathways+ko01110//Biosynthesis of secondary metabolites+ko00260//Glycine, serine and threonine metabolism+ko00270//Cysteine and methionine metabolism+ko01200//Carbon metabolism						
15	EMIHUDRAFT_448960	K01758//cystathionine gamma-lyase [EC:4.4.1.1]+ko01230//Biosynthesis of amino acids+ko00450//Selenocompound metabolism+ko01100//Metabolic pathways+ko01110//Biosynthesis of secondary metabolites+ko00260//Glycine, serine and threonine metabolism+ko00270//Cysteine and methionine metabolism;K01739//cystathionine gamma-synthase [EC:2.5.1.48]+ko00920//Sulfur metabolism+ko01230//Biosynthesis of amino acids+ko00450//Selenocompound metabolism+ko01100//Metabolic pathways+ko01110//Biosynthesis of secondary metabolites+ko00270//Cysteine and methionine metabolism;K01760//cysteine-S-conjugate beta-lyase [EC:4.4.1.13]+ko01230//Biosynthesis of amino acids+ko00450//Selenocompound metabolism+ko01100//Metabolic pathways+ko01110//Biosynthesis of secondary metabolites+ko00270//Cysteine and methionine metabolism	1.0278	4.14E-02	Up	3.3227	1.63E-16	Up
16	EMIHUDRAFT_450594	K01681//aconitate hydratase [EC:4.2.1.3]+ko00020//Citrate cycle (TCA cycle)+ko01230//Biosynthesis of amino acids+ko01100//Metabolic pathways+ko01110//Biosynthesis of secondary metabolites+ko01210//2-Oxocarboxylic acid metabolism+ko01200//Carbon metabolism+ko00630//Glyoxylate and dicarboxylate metabolism;K09566//peptidyl-prolyl isomerase G (cyclophilin G) [EC:5.2.1.8];K13171//serine/arginine repetitive matrix protein 1+ko03015//mRNA surveillance pathway+ko03013//Nucleocytoplasmic transport;K01681//aconitate hydratase [EC:4.2.1.3]+ko00020//Citrate cycle (TCA cycle)+ko01230//Biosynthesis of amino acids+ko01100//Metabolic pathways+ko01110//Biosynthesis of secondary metabolites+ko01210//2-Oxocarboxylic acid metabolism+ko01200//Carbon metabolism+ko00630//Glyoxylate and dicarboxylate metabolism	2.3459	1.15E-05	Up	3.1688	4.99E-12	Up
17	EMIHUDRAFT_451668	K14152//phosphoribosyl-ATP pyrophosphohydrolase / phosphoribosyl-AMP cyclohydrolase / histidinol dehydrogenase [EC:3.6.1.31 3.5.4.19 1.1.1.23]+ko01230//Biosynthesis of amino acids+ko01100//Metabolic pathways+ko00340//Histidine metabolism+ko01110//Biosynthesis of secondary metabolites;K17987//next to BRCA1 gene 1 protein	1.4529	2.20E-02	Up	3.1664	5.30E-15	Up
18	EMIHUDRAFT_456646	K10206//LL-diaminopimelate aminotransferase [EC:2.6.1.83]+ko00300//Lysine biosynthesis+ko01230//Biosynthesis of amino acids+ko01100//Metabolic pathways+ko01110//Biosynthesis of secondary metabolites	1.7242	1.49E-04	Up	3.1633	2.29E-14	Up
19	EMIHUDRAFT_420632	K00058//D-3-phosphoglycerate dehydrogenase / 2-oxoglutarate reductase [EC:1.1.1.95 1.1.1.399]+ko01230//Biosynthesis of amino acids+ko01100//Metabolic pathways+ko01110//Biosynthesis of secondary metabolites+ko00260//Glycine, serine and threonine	1.5507	1.67E-08	Up	3.0695	4.33E-24	Up

		metabolism+ko00270//Cysteine and methionine metabolism+ko01200//Carbon metabolism						
20	EMIHUDRAFT_421493	K14455//aspartate aminotransferase, mitochondrial [EC:2.6.1.1]+ko00250//Alanine, aspartate and glutamate metabolism+ko00220//Arginine biosynthesis+ko00330//Arginine and proline metabolism+ko01230//Biosynthesis of amino acids+ko00960//Tropane, piperidine and pyridine alkaloid biosynthesis+ko00350//Tyrosine metabolism+ko01100//Metabolic pathways+ko00950//Isoquinoline alkaloid biosynthesis+ko01110//Biosynthesis of secondary metabolites+ko00710//Carbon fixation in photosynthetic organisms+ko01210//2-Oxocarboxylic acid metabolism+ko00270//Cysteine and methionine metabolism+ko01200//Carbon metabolism+ko00400//Phenylalanine, tyrosine and tryptophan biosynthesis+ko00360//Phenylalanine metabolism	2.4593	2.31E-08	Up	3.0003	5.94E-15	Up
21	EMIHUDRAFT_455163	K01754//threonine dehydratase [EC:4.3.1.19]+ko00290//Valine, leucine and isoleucine biosynthesis+ko01230//Biosynthesis of amino acids+ko01100//Metabolic pathways+ko01110//Biosynthesis of secondary metabolites+ko00260//Glycine, serine and threonine metabolism+ko01200//Carbon metabolism	1.2620	2.57E-02	Up	2.8743	2.16E-09	Up
22	EMIHUDRAFT_427029	K01649//2-isopropylmalate synthase [EC:2.3.3.13]+ko00290//Valine, leucine and isoleucine biosynthesis+ko01230//Biosynthesis of amino acids+ko01100//Metabolic pathways+ko01110//Biosynthesis of secondary metabolites+ko01210//2-Oxocarboxylic acid metabolism+ko00620//Pyruvate metabolism	1.4566	9.44E-07	Up	2.8324	7.15E-24	Up
23	EMIHUDRAFT_55105	K00640//serine O-acetyltransferase [EC:2.3.1.30]+ko00920//Sulfur metabolism+ko01230//Biosynthesis of amino acids+ko01100//Metabolic pathways+ko01110//Biosynthesis of secondary metabolites+ko00270//Cysteine and methionine metabolism+ko01200//Carbon metabolism	1.1397	2.48E-02	Up	2.7804	7.84E-15	Up
24	EMIHUDRAFT_212438	K01778//diaminopimelate epimerase [EC:5.1.1.7]+ko00300//Lysine biosynthesis+ko01230//Biosynthesis of amino acids+ko01100//Metabolic pathways+ko01110//Biosynthesis of secondary metabolites+ko00470//D-Amino acid metabolism	1.5949	1.52E-03	Up	2.7470	5.71E-13	Up
25	EMIHUDRAFT_456927	K01958//pyruvate carboxylase [EC:6.4.1.1]+ko00020//Citrate cycle (TCA cycle)+ko01230//Biosynthesis of amino acids+ko01100//Metabolic pathways+ko00620//Pyruvate metabolism+ko01200//Carbon metabolism	1.8285	2.34E-06	Up	2.7199	6.41E-13	Up
26	EMIHUDRAFT_351041	K00765//ATP phosphoribosyltransferase [EC:2.4.2.17]+ko01230//Biosynthesis of amino acids+ko01100//Metabolic pathways+ko00340//Histidine metabolism+ko01110//Biosynthesis of secondary metabolites;K13412//calcium-dependent protein kinase [EC:2.7.11.1]+ko04626//Plant-pathogen interaction	1.3179	3.44E-02	Up	2.6441	1.17E-07	Up
27	EMIHUDRAFT_454980	K14152//phosphoribosyl-ATP pyrophosphohydrolase / phosphoribosyl-AMP cyclohydrolase / histidinol dehydrogenase [EC:3.6.1.31 3.5.4.19 1.1.1.23]+ko01230//Biosynthesis of amino acids+ko01100//Metabolic	1.0574	1.03E-01	-	2.5852	3.87E-11	Up

		pathways+ko00340//Histidine metabolism+ko01110//Biosynthesis of secondary metabolites						
28	EMIHUDRAFT_198605	K00818//acetylornithine aminotransferase [EC:2.6.1.11]+ko00220//Arginine biosynthesis+ko01230//Biosynthesis of amino acids+ko01100//Metabolic pathways+ko01110//Biosynthesis of secondary metabolites+ko01210//2-Oxocarboxylic acid metabolism	1.5587	4.56E-02	Up	2.5725	1.62E-06	Up
29	EMIHUDRAFT_198604	K00818//acetylornithine aminotransferase [EC:2.6.1.11]+ko00220//Arginine biosynthesis+ko01230//Biosynthesis of amino acids+ko01100//Metabolic pathways+ko01110//Biosynthesis of secondary metabolites+ko01210//2-Oxocarboxylic acid metabolism	2.0860	8.05E-07	Up	2.5709	1.12E-14	Up
30	BGI_novel_G001893	K01915//glutamine synthetase [EC:6.3.1.2]+ko00250//Alanine, aspartate and glutamate metabolism+ko00910//Nitrogen metabolism+ko00220//Arginine biosynthesis+ko01230//Biosynthesis of amino acids+ko01100//Metabolic pathways+ko00630//Glyoxylate and dicarboxylate metabolism	1.3509	2.65E-01	-	2.5353	1.53E-02	Up
31	EMIHUDRAFT_427526	K01696//tryptophan synthase beta chain [EC:4.2.1.20]+ko01230//Biosynthesis of amino acids+ko01100//Metabolic pathways+ko01110//Biosynthesis of secondary metabolites+ko00260//Glycine, serine and threonine metabolism+ko00400//Phenylalanine, tyrosine and tryptophan biosynthesis	1.5992	3.90E-06	Up	2.5078	1.43E-13	Up
32	EMIHUDRAFT_462938	K01733//threonine synthase [EC:4.2.3.1]+ko00750//Vitamin B6 metabolism+ko01230//Biosynthesis of amino acids+ko01100//Metabolic pathways+ko01110//Biosynthesis of secondary metabolites+ko00260//Glycine, serine and threonine metabolism;K13412//calcium-dependent protein kinase [EC:2.7.11.1]+ko04626//Plant-pathogen interaction	1.4972	3.53E-09	Up	2.4896	1.44E-18	Up
33	EMIHUDRAFT_433725	K00600//glycine hydroxymethyltransferase [EC:2.1.2.1]+ko01240//Biosynthesis of cofactors+ko00670//One carbon pool by folate+ko01230//Biosynthesis of amino acids+ko01100//Metabolic pathways+ko01110//Biosynthesis of secondary metabolites+ko00460//Cyanoamino acid metabolism+ko00260//Glycine, serine and threonine metabolism+ko01200//Carbon metabolism+ko00630//Glyoxylate and dicarboxylate metabolism	0.9269	5.61E-04	-	2.4282	1.91E-15	Up
34	EMIHUDRAFT_449277	K01626//3-deoxy-7-phosphoheptulonate synthase [EC:2.5.1.54]+ko01230//Biosynthesis of amino acids+ko01100//Metabolic pathways+ko01110//Biosynthesis of secondary metabolites+ko00400//Phenylalanine, tyrosine and tryptophan biosynthesis	0.7199	7.91E-02	-	2.3630	1.10E-12	Up
35	EMIHUDRAFT_453479	K01736//chorismate synthase [EC:4.2.3.5]+ko01230//Biosynthesis of amino acids+ko01100//Metabolic pathways+ko01110//Biosynthesis of secondary metabolites+ko00400//Phenylalanine, tyrosine and tryptophan biosynthesis	1.2389	2.70E-02	Up	2.2628	9.16E-09	Up
36	EMIHUDRAFT_457292	K03031//26S proteasome regulatory subunit N12+ko03050//Proteasome;K00611//ornithine carbamoyltransferase [EC:2.1.3.3]+ko00220//Arginine biosynthesis+ko01230//Biosynthesis of amino acids+ko01100//Metabolic pathways+ko01110//Biosynthesis of secondary metabolites	1.4086	2.81E-11	Up	0.8951	9.50E-05	-

37	EMIHUDRAFT_438446	K00831//phosphoserine aminotransferase [EC:2.6.1.52]+ko00750//Vitamin B6 metabolism+ko01240//Biosynthesis of cofactors+ko01230//Biosynthesis of amino acids+ko01100//Metabolic pathways+ko01110//Biosynthesis of secondary metabolites+ko00260//Glycine, serine and threonine metabolism+ko00270//Cysteine and methionine metabolism+ko01200//Carbon metabolism;K09566//peptidyl-prolyl isomerase G (cyclophilin G) [EC:5.2.1.8]; K14306//nuclear pore complex protein Nup62+ko03013//Nucleocytoplasmic transport	0.7389	1.74E-01	-	2.6900	1.03E-15	Up
38	EMIHUDRAFT_442980	K00766//anthranilate phosphoribosyltransferase [EC:2.4.2.18]+ko01230//Biosynthesis of amino acids+ko01100//Metabolic pathways+ko01110//Biosynthesis of secondary metabolites+ko00400//Phenylalanine, tyrosine and tryptophan biosynthesis	0.4375	7.26E-01	-	2.6650	1.11E-07	Up
39	EMIHUDRAFT_462645	K00789//S-adenosylmethionine synthetase [EC:2.5.1.6]+ko00999//Biosynthesis of various plant secondary metabolites+ko01240//Biosynthesis of cofactors+ko01230//Biosynthesis of amino acids+ko01100//Metabolic pathways+ko01110//Biosynthesis of secondary metabolites+ko00270//Cysteine and methionine metabolism	1.3110	1.07E-01	-	2.6624	1.15E-04	Up
40	EMIHUDRAFT_460075	K01758//cystathionine gamma-lyase [EC:4.4.1.1]+ko01230//Biosynthesis of amino acids+ko00450//Selenocompound metabolism+ko01100//Metabolic pathways+ko01110//Biosynthesis of secondary metabolites+ko00260//Glycine, serine and threonine metabolism+ko00270//Cysteine and methionine metabolism;K01739//cystathionine gamma-synthase [EC:2.5.1.48]+ko00920//Sulfur metabolism+ko01230//Biosynthesis of amino acids+ko00450//Selenocompound metabolism+ko01100//Metabolic pathways+ko01110//Biosynthesis of secondary metabolites+ko00270//Cysteine and methionine metabolism	1.0903	8.01E-02	-	2.9632	1.03E-10	Up
41	EMIHUDRAFT_435273	K00831//phosphoserine aminotransferase [EC:2.6.1.52]+ko00750//Vitamin B6 metabolism+ko01240//Biosynthesis of cofactors+ko01230//Biosynthesis of amino acids+ko01100//Metabolic pathways+ko01110//Biosynthesis of secondary metabolites+ko00260//Glycine, serine and threonine metabolism+ko00270//Cysteine and methionine metabolism+ko01200//Carbon metabolism;K09566//peptidyl-prolyl isomerase G (cyclophilin G) [EC:5.2.1.8]	0.5426	2.46E-01	-	2.8790	4.52E-23	Up
42	EMIHUDRAFT_632036	K01755//argininosuccinate lyase [EC:4.3.2.1]+ko00250//Alanine, aspartate and glutamate metabolism+ko00220//Arginine biosynthesis+ko01230//Biosynthesis of amino acids+ko01100//Metabolic pathways+ko01110//Biosynthesis of secondary metabolites	1.8488	9.19E-02	-	3.4073	8.68E-04	Up
43	EMIHUDRAFT_247690	K01626//3-deoxy-7-phosphoheptulonate synthase [EC:2.5.1.54]+ko01230//Biosynthesis of amino acids+ko01100//Metabolic pathways+ko01110//Biosynthesis of secondary metabolites+ko00400//Phenylalanine, tyrosine and tryptophan biosynthesis	0.5210	3.56E-01	-	2.1860	3.93E-12	Up
44	EMIHUDRA	K12524//bifunctional aspartokinase / homoserine dehydrogenase 1 [EC:2.7.2.4 1.1.1.3]+ko00300//Lysine biosynthesis+ko01230//Biosynthesis of amino acids+ko01100//Metabolic pathways+ko01110//Biosynthesis of secondary	0.5880	9.37E-02	-	2.1172	5.10E-15	Up

	FT_456186	metabolites+ko00261//Monobactam biosynthesis+ko00260//Glycine, serine and threonine metabolism+ko00270//Cysteine and methionine metabolism;K00003//homoserine dehydrogenase [EC:1.1.1.3]+ko00300//Lysine biosynthesis+ko01230//Biosynthesis of amino acids+ko01100//Metabolic pathways+ko01110//Biosynthesis of secondary metabolites+ko00260//Glycine, serine and threonine metabolism+ko00270//Cysteine and methionine metabolism							
45	EMIHUDRAFT_439707	K00766//anthranilate phosphoribosyltransferase [EC:2.4.2.18]+ko01230//Biosynthesis of amino acids+ko01100//Metabolic pathways+ko01110//Biosynthesis of secondary metabolites+ko00400//Phenylalanine, tyrosine and tryptophan biosynthesis	0.5926	3.64E-01	-	2.0509	1.49E-09	Up
46	EMIHUDRAFT_106701	K01739//cystathionine gamma-synthase [EC:2.5.1.48]+ko00920//Sulfur metabolism+ko01230//Biosynthesis of amino acids+ko00450//Selenocompound metabolism+ko01100//Metabolic pathways+ko01110//Biosynthesis of secondary metabolites+ko00270//Cysteine and methionine metabolism;K01758//cystathionine gamma-lyase [EC:4.4.1.1]+ko01230//Biosynthesis of amino acids+ko00450//Selenocompound metabolism+ko01100//Metabolic pathways+ko01110//Biosynthesis of secondary metabolites+ko00260//Glycine, serine and threonine metabolism+ko00270//Cysteine and methionine metabolism	0.9714	7.94E-02	-	1.9932	6.45E-06	Up
47	EMIHUDRAFT_423073	K00548//5-methyltetrahydrofolate--homocysteine methyltransferase [EC:2.1.1.13]+ko00670//One carbon pool by folate+ko01230//Biosynthesis of amino acids+ko00450//Selenocompound metabolism+ko01100//Metabolic pathways+ko01110//Biosynthesis of secondary metabolites+ko00270//Cysteine and methionine metabolism	0.4005	2.19E-01	-	1.9514	1.46E-12	Up
48	EMIHUDRAFT_453474	K01736//chorismate synthase [EC:4.2.3.5]+ko01230//Biosynthesis of amino acids+ko01100//Metabolic pathways+ko01110//Biosynthesis of secondary metabolites+ko00400//Phenylalanine, tyrosine and tryptophan biosynthesis	0.1755	8.16E-01	-	1.9369	1.97E-10	Up
49	BGI_novel_G000605	K01586//diaminopimelate decarboxylase [EC:4.1.1.20]+ko00300//Lysine biosynthesis+ko01230//Biosynthesis of amino acids+ko01100//Metabolic pathways+ko01110//Biosynthesis of secondary metabolites+ko00470//D-Amino acid metabolism	1.3393	6.47E-02	-	1.9096	2.72E-04	Up
50	EMIHUDRAFT_437725	K01714//4-hydroxy-tetrahydrodipicolinate synthase [EC:4.3.3.7]+ko00300//Lysine biosynthesis+ko01230//Biosynthesis of amino acids+ko01100//Metabolic pathways+ko01110//Biosynthesis of secondary metabolites+ko00261//Monobactam biosynthesis	0.8505	5.09E-02	-	1.8907	1.32E-09	Up
51	EMIHUDRAFT_423950	K01687//dihydroxy-acid dehydratase [EC:4.2.1.9]+ko00290//Valine, leucine and isoleucine biosynthesis+ko00770//Pantothenate and CoA biosynthesis+ko01230//Biosynthesis of amino acids+ko01100//Metabolic pathways+ko01110//Biosynthesis of secondary metabolites+ko01210//2-Oxocarboxylic acid metabolism	0.7574	7.93E-02	-	1.8697	2.19E-09	Up
52	EMIHUDRA	K01739//cystathionine gamma-synthase [EC:2.5.1.48]+ko00920//Sulfur metabolism+ko01230//Biosynthesis of amino acids+ko00450//Selenocompound	0.7169	2.81E-01	-	1.8508	7.79E-06	Up

53	EMIHUDRAFT_224002	metabolism+ko01100//Metabolic pathways+ko01110//Biosynthesis of secondary metabolites+ko00270//Cysteine and methionine metabolism K00831//phosphoserine aminotransferase [EC:2.6.1.52]+ko00750//Vitamin B6 metabolism+ko01240//Biosynthesis of cofactors+ko01230//Biosynthesis of amino acids+ko01100//Metabolic pathways+ko01110//Biosynthesis of secondary metabolites+ko00260//Glycine, serine and threonine metabolism+ko00270//Cysteine and methionine metabolism+ko01200//Carbon metabolism;K13412//calcium-dependent protein kinase [EC:2.7.11.1]+ko04626//Plant-pathogen interaction;K09566//peptidyl-prolyl isomerase G (cyclophilin G) [EC:5.2.1.8]	0.2424	7.56E-01	-	1.8264	1.83E-08	Up
	EMIHUDRAFT_452985							
54	EMIHUDRAFT_252925	K12524//bifunctional aspartokinase / homoserine dehydrogenase 1 [EC:2.7.2.4 1.1.1.3]+ko00300//Lysine biosynthesis+ko01230//Biosynthesis of amino acids+ko01100//Metabolic pathways+ko01110//Biosynthesis of secondary metabolites+ko00261//Monobactam biosynthesis+ko00260//Glycine, serine and threonine metabolism+ko00270//Cysteine and methionine metabolism	0.5214	4.81E-01	-	1.7631	1.72E-06	Up
55	EMIHUDRAFT_470315	K01850//chorismate mutase [EC:5.4.99.5]+ko01230//Biosynthesis of amino acids+ko01100//Metabolic pathways+ko01110//Biosynthesis of secondary metabolites+ko00400//Phenylalanine, tyrosine and tryptophan biosynthesis	0.4510	3.19E-01	-	1.7314	3.93E-12	Up
56	EMIHUDRAFT_77494	K01758//cystathionine gamma-lyase [EC:4.4.1.1]+ko01230//Biosynthesis of amino acids+ko00450//Selenocompound metabolism+ko01100//Metabolic pathways+ko01110//Biosynthesis of secondary metabolites+ko00260//Glycine, serine and threonine metabolism+ko00270//Cysteine and methionine metabolism;K17069//O-acetylhomoserine/O-acetylserine sulfhydrylase [EC:2.5.1.49 2.5.1.47]+ko00920//Sulfur metabolism+ko01230//Biosynthesis of amino acids+ko01100//Metabolic pathways+ko01110//Biosynthesis of secondary metabolites+ko00270//Cysteine and methionine metabolism+ko01200//Carbon metabolism;K01760//cysteine-S-conjugate beta-lyase [EC:4.4.1.13]+ko01230//Biosynthesis of amino acids+ko00450//Selenocompound metabolism+ko01100//Metabolic pathways+ko01110//Biosynthesis of secondary metabolites+ko00270//Cysteine and methionine metabolism	0.9386	1.09E-01	-	3.8479	2.93E-19	Up
57	EMIHUDRAFT_460784	K14325//RNA-binding protein with serine-rich domain 1+ko03015//mRNA surveillance pathway+ko03013//Nucleocytoplasmic transport;K13172//serine/arginine repetitive matrix protein 2;K13171//serine/arginine repetitive matrix protein 1+ko03015//mRNA surveillance pathway+ko03013//Nucleocytoplasmic transport;K14325//RNA-binding protein with serine-rich domain 1+ko03015//mRNA surveillance pathway+ko03013//Nucleocytoplasmic transport; K12524//bifunctional aspartokinase / homoserine dehydrogenase 1 [EC:2.7.2.4 1.1.1.3]+ko00300//Lysine biosynthesis+ko01230//Biosynthesis of amino acids+ko01100//Metabolic pathways+ko01110//Biosynthesis of secondary metabolites+ko00261//Monobactam biosynthesis+ko00260//Glycine, serine and threonine metabolism+ko00270//Cysteine and methionine metabolism	1.3571	2.23E-01	-	3.8022	4.22E-09	Up

58	EMIHUDRAFT_230458	K00053//ketol-acid reductoisomerase [EC:1.1.1.86]+ko00290//Valine, leucine and isoleucine biosynthesis+ko00770//Pantothenate and CoA biosynthesis+ko01230//Biosynthesis of amino acids+ko01100//Metabolic pathways+ko01110//Biosynthesis of secondary metabolites+ko01210//2-Oxocarboxylic acid metabolism	1.2707	2.96E-01	-	3.7022	6.23E-07	Up
59	EMIHUDRAFT_245556	K13501//anthranilate synthase / indole-3-glycerol phosphate synthase / phosphoribosylanthranilate isomerase [EC:4.1.3.27 4.1.1.48 5.3.1.24]+ko01230//Biosynthesis of amino acids+ko01100//Metabolic pathways+ko01110//Biosynthesis of secondary metabolites+ko00400//Phenylalanine, tyrosine and tryptophan biosynthesis;K01609//indole-3-glycerol phosphate synthase [EC:4.1.1.48]+ko01230//Biosynthesis of amino acids+ko01100//Metabolic pathways+ko01110//Biosynthesis of secondary metabolites+ko00400//Phenylalanine, tyrosine and tryptophan biosynthesis	0.5943	4.74E-01	-	1.7050	9.85E-05	Up
60	EMIHUDRAFT_462681	K01760//cysteine-S-conjugate beta-lyase [EC:4.4.1.13]+ko01230//Biosynthesis of amino acids+ko00450//Selenocompound metabolism+ko01100//Metabolic pathways+ko01110//Biosynthesis of secondary metabolites+ko00270//Cysteine and methionine metabolism;K13172//serine/arginine repetitive matrix protein 2;K09566//peptidyl-prolyl isomerase G (cyclophilin G) [EC:5.2.1.8]	0.0181	9.89E-01	-	1.6674	2.30E-04	Up
61	EMIHUDRAFT_467437	K00284//glutamate synthase (ferredoxin) [EC:1.4.7.1]+ko00910//Nitrogen metabolism+ko00630//Glyoxylate and dicarboxylate metabolism;K00264//glutamate synthase (NADH) [EC:1.4.1.14]+ko00250//Alanine, aspartate and glutamate metabolism+ko00910//Nitrogen metabolism+ko01230//Biosynthesis of amino acids+ko01100//Metabolic pathways+ko01110//Biosynthesis of secondary metabolites	0.2349	7.70E-01	-	1.6599	2.28E-05	Up
62	EMIHUDRAFT_454633	K15849//bifunctional aspartate aminotransferase and glutamate/aspartate-prephenate aminotransferase [EC:2.6.1.1 2.6.1.78 2.6.1.79]+ko01230//Biosynthesis of amino acids+ko00960//Tropane, piperidine and pyridine alkaloid biosynthesis+ko00350//Tyrosine metabolism+ko01100//Metabolic pathways+ko00950//Isoquinoline alkaloid biosynthesis+ko01110//Biosynthesis of secondary metabolites+ko00400//Phenylalanine, tyrosine and tryptophan biosynthesis+ko00360//Phenylalanine metabolism;K05359//arogenate/prephenate dehydratase [EC:4.2.1.91 4.2.1.51]+ko01230//Biosynthesis of amino acids+ko01100//Metabolic pathways+ko01110//Biosynthesis of secondary metabolites+ko00400//Phenylalanine, tyrosine and tryptophan biosynthesis;K15227//arogenate dehydrogenase (NADP+), plant [EC:1.3.1.78]+ko01230//Biosynthesis of amino acids+ko01100//Metabolic pathways+ko01110//Biosynthesis of secondary metabolites+ko00400//Phenylalanine, tyrosine and tryptophan biosynthesis	0.2319	7.19E-01	-	1.6512	2.17E-10	Up
63	EMIHUDRA	K01647//citrate synthase [EC:2.3.3.1]+ko00020//Citrate cycle (TCA cycle)+ko01230//Biosynthesis of amino acids+ko01100//Metabolic	0.4837	1.72E-01	-	1.6404	5.36E-07	Up

	FT_46 7883	pathways+ko01110//Biosynthesis of secondary metabolites+ko01210//2-Oxocarboxylic acid metabolism+ko01200//Carbon metabolism+ko00630//Glyoxylate and dicarboxylate metabolism						
64	EMIH UDRA FT_45 3481	K01736//chorismate synthase [EC:4.2.3.5]+ko01230//Biosynthesis of amino acids+ko01100//Metabolic pathways+ko01110//Biosynthesis of secondary metabolites+ko00400//Phenylalanine, tyrosine and tryptophan biosynthesis	0.3357	6.08E-01	-	1.6110	1.75E-06	Up
65	EMIH UDRA FT_45 5983	K01953//asparagine synthase (glutamine-hydrolysing) [EC:6.3.5.4]+ko00250//Alanine, aspartate and glutamate metabolism+ko01230//Biosynthesis of amino acids+ko01100//Metabolic pathways+ko01110//Biosynthesis of secondary metabolites;K07390//monothiol glutaredoxin	0.3440	8.30E-01	-	1.6074	1.63E-02	Up
66	EMIH UDRA FT_61 710	K00286//pyrroline-5-carboxylate reductase [EC:1.5.1.2]+ko00330//Arginine and proline metabolism+ko01230//Biosynthesis of amino acids+ko01100//Metabolic pathways+ko01110//Biosynthesis of secondary metabolites	-0.1868	8.90E-01	-	1.5863	2.53E-03	Up
67	EMIH UDRA FT_46 0677	K01738//cysteine synthase [EC:2.5.1.47]+ko00920//Sulfur metabolism+ko01230//Biosynthesis of amino acids+ko01100//Metabolic pathways+ko01110//Biosynthesis of secondary metabolites+ko00270//Cysteine and methionine metabolism+ko01200//Carbon metabolism	0.6061	7.77E-02	-	1.5719	4.21E-09	Up
68	BGI_no vel_G0 04709	K00823//4-aminobutyrate aminotransferase [EC:2.6.1.19]+ko00410//beta-Alanine metabolism+ko00250//Alanine, aspartate and glutamate metabolism+ko00640//Propanoate metabolism+ko01100//Metabolic pathways+ko00650//Butanoate metabolism;K00818//acetylornithine aminotransferase [EC:2.6.1.11]+ko00220//Arginine biosynthesis+ko01230//Biosynthesis of amino acids+ko01100//Metabolic pathways+ko01110//Biosynthesis of secondary metabolites+ko01210//2-Oxocarboxylic acid metabolism	0.1671	8.61E-01	-	1.5611	8.61E-05	Up
69	EMIH UDRA FT_23 6897	K09566//peptidyl-prolyl isomerase G (cyclophilin G) [EC:5.2.1.8];K12524//bifunctional aspartokinase / homoserine dehydrogenase 1 [EC:2.7.2.4 1.1.1.3]+ko00300//Lysine biosynthesis+ko01230//Biosynthesis of amino acids+ko01100//Metabolic pathways+ko01110//Biosynthesis of secondary metabolites+ko00261//Monobactam biosynthesis+ko00260//Glycine, serine and threonine metabolism+ko00270//Cysteine and methionine metabolism;K13172//serine/arginine repetitive matrix protein 2;K09566//peptidyl-prolyl isomerase G (cyclophilin G) [EC:5.2.1.8]	-0.0628	9.52E-01	-	1.5412	6.85E-06	Up
70	BGI_no vel_G0 01602	K01915//glutamine synthetase [EC:6.3.1.2]+ko00250//Alanine, aspartate and glutamate metabolism+ko00910//Nitrogen metabolism+ko00220//Arginine biosynthesis+ko01230//Biosynthesis of amino acids+ko01100//Metabolic pathways+ko00630//Glyoxylate and dicarboxylate metabolism	0.3507	4.64E-01	-	1.4656	1.97E-08	Up
71	EMIH UDRA	K00817//histidinol-phosphate aminotransferase [EC:2.6.1.9]+ko01230//Biosynthesis of amino acids+ko00960//Tropane, piperidine and pyridine alkaloid biosynthesis+ko00350//Tyrosine	0.5152	5.34E-01	-	1.4605	1.43E-03	Up

	FT_42 5759	metabolism+ko01100//Metabolic pathways+ko00340//Histidine metabolism+ko01110//Biosynthesis of secondary metabolites+ko00400//Phenylalanine, tyrosine and tryptophan biosynthesis+ko00360//Phenylalanine metabolism						
72	EMIH UDRA FT_42 6730	K01663//imidazole glycerol-phosphate synthase [EC:4.3.2.10]+ko01230//Biosynthesis of amino acids+ko01100//Metabolic pathways+ko00340//Histidine metabolism+ko01110//Biosynthesis of secondary metabolites	0.5905	1.10E-01	-	1.4593	1.25E-07	Up
73	EMIH UDRA FT_45 2198	K01738//cysteine synthase [EC:2.5.1.47]+ko00920//Sulfur metabolism+ko01230//Biosynthesis of amino acids+ko01100//Metabolic pathways+ko01110//Biosynthesis of secondary metabolites+ko00270//Cysteine and methionine metabolism+ko01200//Carbon metabolism	0.5083	1.40E-01	-	1.3505	2.33E-07	Up
74	EMIH UDRA FT_44 1299	K01940//argininosuccinate synthase [EC:6.3.4.5]+ko00250//Alanine, aspartate and glutamate metabolism+ko00220//Arginine biosynthesis+ko01230//Biosynthesis of amino acids+ko01100//Metabolic pathways+ko01110//Biosynthesis of secondary metabolites	-0.1260	7.96E-01	-	1.3497	6.08E-07	Up
75	EMIH UDRA FT_72 956	K01915//glutamine synthetase [EC:6.3.1.2]+ko00250//Alanine, aspartate and glutamate metabolism+ko00910//Nitrogen metabolism+ko00220//Arginine biosynthesis+ko01230//Biosynthesis of amino acids+ko01100//Metabolic pathways+ko00630//Glyoxylate and dicarboxylate metabolism	0.6242	4.76E-01	-	1.2966	9.14E-03	Up
76	EMIH UDRA FT_55 024	K00640//serine O-acetyltransferase [EC:2.3.1.30]+ko00920//Sulfur metabolism+ko01230//Biosynthesis of amino acids+ko01100//Metabolic pathways+ko01110//Biosynthesis of secondary metabolites+ko00270//Cysteine and methionine metabolism+ko01200//Carbon metabolism	0.8800	2.14E-01	-	1.2894	1.54E-02	Up
77	EMIH UDRA FT_46 3702	K01657//anthranilate synthase component I [EC:4.1.3.27]+ko01230//Biosynthesis of amino acids+ko01100//Metabolic pathways+ko01110//Biosynthesis of secondary metabolites+ko00400//Phenylalanine, tyrosine and tryptophan biosynthesis;K01658//anthranilate synthase component II [EC:4.1.3.27]+ko01230//Biosynthesis of amino acids+ko01100//Metabolic pathways+ko01110//Biosynthesis of secondary metabolites+ko00400//Phenylalanine, tyrosine and tryptophan biosynthesis	0.1271	8.37E-01	-	1.2843	4.00E-07	Up
78	BGI_no vel_G0 01601	K01915//glutamine synthetase [EC:6.3.1.2]+ko00250//Alanine, aspartate and glutamate metabolism+ko00910//Nitrogen metabolism+ko00220//Arginine biosynthesis+ko01230//Biosynthesis of amino acids+ko01100//Metabolic pathways+ko00630//Glyoxylate and dicarboxylate metabolism;K14325//RNA-binding protein with serine-rich domain 1+ko03015//mRNA surveillance pathway+ko03013//Nucleocytoplasmic transport;K21952//CASK-interacting protein	-0.1618	8.62E-01	-	1.2634	2.60E-06	Up
79	EMIH UDRA FT_46 9498	K00264//glutamate synthase (NADH) [EC:1.4.1.14]+ko00250//Alanine, aspartate and glutamate metabolism+ko00910//Nitrogen metabolism+ko01230//Biosynthesis of amino acids+ko01100//Metabolic pathways+ko01110//Biosynthesis of secondary metabolites;K00264//glutamate synthase (NADH) [EC:1.4.1.14]+ko00250//Alanine, aspartate and glutamate	-0.1387	8.42E-01	-	1.2159	7.57E-06	Up

		metabolism+ko00910//Nitrogen metabolism+ko01230//Biosynthesis of amino acids+ko01100//Metabolic pathways+ko01110//Biosynthesis of secondary metabolites						
80	EMIHUDRAFT_450554	K13172//serine/arginine repetitive matrix protein 2;K01626//3-deoxy-7-phosphoheptulonate synthase [EC:2.5.1.54]+ko01230//Biosynthesis of amino acids+ko01100//Metabolic pathways+ko01110//Biosynthesis of secondary metabolites+ko00400//Phenylalanine, tyrosine and tryptophan biosynthesis	0.0358	9.58E-01	-	1.1478	7.85E-05	Up
81	EMIHUDRAFT_72297	K00826//branched-chain amino acid aminotransferase [EC:2.6.1.42]+ko00290//Valine, leucine and isoleucine biosynthesis+ko01240//Biosynthesis of cofactors+ko00280//Valine, leucine and isoleucine degradation+ko00770//Pantothenate and CoA biosynthesis+ko01230//Biosynthesis of amino acids+ko00966//Glucosinolate biosynthesis+ko01100//Metabolic pathways+ko01110//Biosynthesis of secondary metabolites+ko01210//2-Oxocarboxylic acid metabolism+ko00270//Cysteine and methionine metabolism	0.9921	1.48E-03	-	1.1461	1.80E-04	Up
82	EMIHUDRAFT_68131	K00826//branched-chain amino acid aminotransferase [EC:2.6.1.42]+ko00290//Valine, leucine and isoleucine biosynthesis+ko01240//Biosynthesis of cofactors+ko00280//Valine, leucine and isoleucine degradation+ko00770//Pantothenate and CoA biosynthesis+ko01230//Biosynthesis of amino acids+ko00966//Glucosinolate biosynthesis+ko01100//Metabolic pathways+ko01110//Biosynthesis of secondary metabolites+ko01210//2-Oxocarboxylic acid metabolism+ko00270//Cysteine and methionine metabolism	0.9921	1.48E-03	-	1.1461	1.80E-04	Up
83	EMIHUDRAFT_447795	K00814//alanine transaminase [EC:2.6.1.2]+ko00250//Alanine, aspartate and glutamate metabolism+ko00220//Arginine biosynthesis+ko01230//Biosynthesis of amino acids+ko01100//Metabolic pathways+ko00710//Carbon fixation in photosynthetic organisms+ko01210//2-Oxocarboxylic acid metabolism+ko01200//Carbon metabolism	0.0772	8.71E-01	-	1.0857	1.72E-05	Up
84	EMIHUDRAFT_632235	K13830//pentafunctional AROM polypeptide [EC:4.2.3.4 4.2.1.10 1.1.1.25 2.7.1.71 2.5.1.19]+ko00999//Biosynthesis of various plant secondary metabolites+ko01230//Biosynthesis of amino acids+ko01100//Metabolic pathways+ko01110//Biosynthesis of secondary metabolites+ko00400//Phenylalanine, tyrosine and tryptophan biosynthesis;K00800//3-phosphoshikimate 1-carboxyvinyltransferase [EC:2.5.1.19]+ko01230//Biosynthesis of amino acids+ko01100//Metabolic pathways+ko01110//Biosynthesis of secondary metabolites+ko00400//Phenylalanine, tyrosine and tryptophan biosynthesis;K24175//MFS transporter, MFS domain-containing protein family, molybdate-anion transporter	0.2432	5.70E-01	-	1.0301	2.46E-05	Up
85	EMIHUDRAFT_466466	K00800//3-phosphoshikimate 1-carboxyvinyltransferase [EC:2.5.1.19]+ko01230//Biosynthesis of amino acids+ko01100//Metabolic pathways+ko01110//Biosynthesis of secondary metabolites+ko00400//Phenylalanine, tyrosine and tryptophan biosynthesis	-0.1856	7.63E-01	-	1.0089	7.06E-05	Up

86	EMIHUDRAFT_631875	K14454//aspartate aminotransferase, cytoplasmic [EC:2.6.1.1]+ko00250//Alanine, aspartate and glutamate metabolism+ko00220//Arginine biosynthesis+ko00330//Arginine and proline metabolism+ko01230//Biosynthesis of amino acids+ko00960//Tropane, piperidine and pyridine alkaloid biosynthesis+ko00350//Tyrosine metabolism+ko01100//Metabolic pathways+ko00950//Isoquinoline alkaloid biosynthesis+ko01110//Biosynthesis of secondary metabolites+ko00710//Carbon fixation in photosynthetic organisms+ko01210//2-Oxocarboxylic acid metabolism+ko00270//Cysteine and methionine metabolism+ko01200//Carbon metabolism+ko00400//Phenylalanine, tyrosine and tryptophan biosynthesis+ko00360//Phenylalanine metabolism;K14455//aspartate aminotransferase, mitochondrial [EC:2.6.1.1]+ko00250//Alanine, aspartate and glutamate metabolism+ko00220//Arginine biosynthesis+ko00330//Arginine and proline metabolism+ko01230//Biosynthesis of amino acids+ko00960//Tropane, piperidine and pyridine alkaloid biosynthesis+ko00350//Tyrosine metabolism+ko01100//Metabolic pathways+ko00950//Isoquinoline alkaloid biosynthesis+ko01110//Biosynthesis of secondary metabolites+ko00710//Carbon fixation in photosynthetic organisms+ko01210//2-Oxocarboxylic acid metabolism+ko00270//Cysteine and methionine metabolism+ko01200//Carbon metabolism+ko00400//Phenylalanine, tyrosine and tryptophan biosynthesis+ko00360//Phenylalanine metabolism	-1.0242	2.36E-05	Down	0.6598	1.43E-02	-
87	EMIHUDRAFT_57193	K11755//phosphoribosyl-AMP cyclohydrolase / phosphoribosyl-ATP pyrophosphohydrolase [EC:3.5.4.19 3.6.1.31]+ko01230//Biosynthesis of amino acids+ko01100//Metabolic pathways+ko00340//Histidine metabolism+ko01110//Biosynthesis of secondary metabolites	-1.1252	1.48E-05	Down	-0.8580	1.87E-03	-
88	EMIHUDRAFT_42684	K01738//cysteine synthase [EC:2.5.1.47]+ko00920//Sulfur metabolism+ko01230//Biosynthesis of amino acids+ko01100//Metabolic pathways+ko01110//Biosynthesis of secondary metabolites+ko00270//Cysteine and methionine metabolism+ko01200//Carbon metabolism	-1.0825	1.69E-02	Down	-0.8743	1.94E-02	-
89	EMIHUDRAFT_53381	K00826//branched-chain amino acid aminotransferase [EC:2.6.1.42]+ko00290//Valine, leucine and isoleucine biosynthesis+ko01240//Biosynthesis of cofactors+ko00280//Valine, leucine and isoleucine degradation+ko00770//Pantothenate and CoA biosynthesis+ko01230//Biosynthesis of amino acids+ko00966//Glucosinolate biosynthesis+ko01100//Metabolic pathways+ko01110//Biosynthesis of secondary metabolites+ko01210//2-Oxocarboxylic acid metabolism+ko00270//Cysteine and methionine metabolism	0.5837	7.85E-02	-	-1.3458	6.58E-05	Down
90	EMIHUDRAFT_242450	K13412//calcium-dependent protein kinase [EC:2.7.11.1]+ko04626//Plant-pathogen interaction;K01620//threonine aldolase [EC:4.1.2.48]+ko01230//Biosynthesis of amino acids+ko01100//Metabolic pathways+ko01110//Biosynthesis of secondary metabolites+ko00260//Glycine, serine and threonine metabolism;K13172//serine/arginine repetitive matrix protein 2	-2.2024	1.31E-15	Up	-1.4390	3.20E-06	Down

91	EMIHUDRAFT_455283	K01703//3-isopropylmalate/(R)-2-methylmalate dehydratase large subunit [EC:4.2.1.33 4.2.1.35]+ko00290//Valine, leucine and isoleucine biosynthesis+ko01230//Biosynthesis of amino acids+ko00966//Glucosinolate biosynthesis+ko01100//Metabolic pathways+ko01110//Biosynthesis of secondary metabolites+ko01210//2-Oxocarboxylic acid metabolism+ko00660//C5-Branched dibasic acid metabolism;K11416//NAD+-dependent protein deacetylase sirtuin 6 [EC:2.3.1.286]+ko01100//Metabolic pathways+ko00760//Nicotinate and nicotinamide metabolism	-2.0719	8.38E-16	Up	-2.0626	5.23E-13	Down
92	EMIHUDRAFT_414778	K01687//dihydroxy-acid dehydratase [EC:4.2.1.9]+ko00290//Valine, leucine and isoleucine biosynthesis+ko00770//Pantothenate and CoA biosynthesis+ko01230//Biosynthesis of amino acids+ko01100//Metabolic pathways+ko01110//Biosynthesis of secondary metabolites+ko01210//2-Oxocarboxylic acid metabolism	2.3208	5.44E-03	Up	1.3451	2.23E-01	-
93	EMIHUDRAFT_72929	K01758//cystathionine gamma-lyase [EC:4.4.1.1]+ko01230//Biosynthesis of amino acids+ko00450//Selenocompound metabolism+ko01100//Metabolic pathways+ko01110//Biosynthesis of secondary metabolites+ko00260//Glycine, serine and threonine metabolism+ko00270//Cysteine and methionine metabolism;K01760//cysteine-S-conjugate beta-lyase [EC:4.4.1.13]+ko01230//Biosynthesis of amino acids+ko00450//Selenocompound metabolism+ko01100//Metabolic pathways+ko01110//Biosynthesis of secondary metabolites+ko00270//Cysteine and methionine metabolism	1.7627	1.11E-06	Up	0.8056	6.51E-02	-
94	EMIHUDRAFT_64817	K01758//cystathionine gamma-lyase [EC:4.4.1.1]+ko01230//Biosynthesis of amino acids+ko00450//Selenocompound metabolism+ko01100//Metabolic pathways+ko01110//Biosynthesis of secondary metabolites+ko00260//Glycine, serine and threonine metabolism+ko00270//Cysteine and methionine metabolism;K01760//cysteine-S-conjugate beta-lyase [EC:4.4.1.13]+ko01230//Biosynthesis of amino acids+ko00450//Selenocompound metabolism+ko01100//Metabolic pathways+ko01110//Biosynthesis of secondary metabolites+ko00270//Cysteine and methionine metabolism	1.7281	4.60E-07	Up	0.5694	2.24E-01	-
95	EMIHUDRAFT_216734	K00134//glyceraldehyde 3-phosphate dehydrogenase (phosphorylating) [EC:1.2.1.12]+ko00010//Glycolysis / Gluconeogenesis+ko01230//Biosynthesis of amino acids+ko01100//Metabolic pathways+ko01110//Biosynthesis of secondary metabolites+ko00710//Carbon fixation in photosynthetic organisms+ko01200//Carbon metabolism;K09566//peptidyl-prolyl isomerase G (cyclophilin G) [EC:5.2.1.8];K03128//transcription initiation factor TFIID subunit 2+ko03022//Basal transcription factors;K14325//RNA-binding protein with serine-rich domain 1+ko03015//mRNA surveillance pathway+ko03013//Nucleocytoplasmic transport;K10747//DNA ligase 1 [EC:6.5.1.1 6.5.1.6 6.5.1.7]+ko03420//Nucleotide excision repair+ko03030//DNA replication+ko03410//Base excision repair+ko03430//Mismatch repair	1.4152	1.79E-09	Up	-0.3552	3.31E-01	-
96	EMIHUDRA	K13171//serine/arginine repetitive matrix protein 1+ko03015//mRNA surveillance pathway+ko03013//Nucleocytoplasmic transport;K01739//cystathionine gamma-synthase [EC:2.5.1.48]+ko00920//Sulfur	1.2987	1.65E-11	Up	-0.3987	1.23E-01	-

	FT_20 4967	metabolism+ko01230//Biosynthesis of amino acids+ko00450//Selenocompound metabolism+ko01100//Metabolic pathways+ko01110//Biosynthesis of secondary metabolites+ko00270//Cysteine and methionine metabolism;K10990//RecQ-mediated genome instability protein 1						
97	EMIH UDRA FT_55 8314	K01958//pyruvate carboxylase [EC:6.4.1.1]+ko00020//Citrate cycle (TCA cycle)+ko01230//Biosynthesis of amino acids+ko01100//Metabolic pathways+ko00620//Pyruvate metabolism+ko01200//Carbon metabolism	1.1159	4.69E-02	Up	0.6621	2.97E-01	-
98	EMIH UDRA FT_34 9043	K00286//pyrroline-5-carboxylate reductase [EC:1.5.1.2]+ko00330//Arginine and proline metabolism+ko01230//Biosynthesis of amino acids+ko01100//Metabolic pathways+ko01110//Biosynthesis of secondary metabolites	-1.3804	9.81E-04	Down	0.0778	8.91E-01	-
99	EMIH UDRA FT_44 4464	K14325//RNA-binding protein with serine-rich domain 1+ko03015//mRNA surveillance pathway+ko03013//Nucleocytoplasmic transport;K01739//cystathionine gamma-synthase [EC:2.5.1.48]+ko00920//Sulfur metabolism+ko01230//Biosynthesis of amino acids+ko00450//Selenocompound metabolism+ko01100//Metabolic pathways+ko01110//Biosynthesis of secondary metabolites+ko00270//Cysteine and methionine metabolism	-1.5250	1.78E-02	Down	-0.9399	1.10E-01	-
100	EMIH UDRA FT_43 0252	K01738//cysteine synthase [EC:2.5.1.47]+ko00920//Sulfur metabolism+ko01230//Biosynthesis of amino acids+ko01100//Metabolic pathways+ko01110//Biosynthesis of secondary metabolites+ko00270//Cysteine and methionine metabolism+ko01200//Carbon metabolism	-1.8140	3.29E-02	Down	-0.8328	5.91E-01	-

[a]Log_2 fold change based on RNA-seq data. Each value is the mean from three biological replicates.

[b]Equal to adjusted p-value, change is set at q-value < 0.05 in this study.

Table S8. DEGs involved in nitrogen metabolism in PA/P+ and (P+PA)/P+ comparisons.

No.	Gene ID	Gene name	Description	PA/P+			(P+PA)/P+		
				Log2 FC[a]	q-value[b]	Regulation	Log2 FC[a]	q-value[b]	Regulation
1	EMIHUDRAFT_311732	NRT, nitrate transporter	K02575//MFS transporter, NNP family, nitrate/nitrite transporter+ko00910//Nitrogen metabolism	1.6683	9.99E-03	Up	3.2520	2.20E-10	Up
2	EMIHUDRAFT_62811	NRT, nitrate transporter	K02575//MFS transporter, NNP family, nitrate/nitrite transporter+ko00910//Nitrogen metabolism	1.4263	1.26E-01	-	1.9697	2.17E-02	Up
3	EMIHUDRAFT_361445	NRT, nitrate transporter	K02575//MFS transporter, NNP family, nitrate/nitrite transporter+ko00910//Nitrogen metabolism	1.6543	2.09E-02	Up	2.2611	8.12E-04	Up
4	EMIHUDRAFT_440685	NRT, nitrate transporter	K02575//MFS transporter, NNP family, nitrate/nitrite transporter+ko00910//Nitrogen metabolism	1.2095	1.85E-05	Up	1.2171	3.98E-05	Up
5	EMIHUDRAFT_73160	NRT, nitrate transporter	K02575//MFS transporter, NNP family, nitrate/nitrite transporter+ko00910//Nitrogen metabolism	2.1861	7.15E-07	Up	2.4234	1.31E-08	Up
6	EMIHUDRAFT_460408	NRT, nitrate transporter	K02575//MFS transporter, NNP family, nitrate/nitrite transporter+ko00910//Nitrogen metabolism	1.5107	7.91E-02	-	1.9566	1.47E-02	Up
7	EMIHUDRAFT_451136	AMT, ammonium transporter	K03320//ammonium transporter, Amt family;K14306//nuclear pore complex protein Nup62+ko03013//Nucleocytoplasmic transport;K13680//beta-mannan synthase [EC:2.4.1.32]	-0.7178	3.21E-03	-	-1.5300	6.88E-10	Down
8	EMIHUDRAFT_452779	AMT, ammonium transporter	K03320//ammonium transporter, Amt family;K17987//next to BRCA1 gene 1 protein	-0.9780	4.82E-05	-	-1.5061	1.83E-08	Down
9	EMIHUDRAFT_74037	AMT, ammonium transporter	K03320//ammonium transporter, Amt family	1.7598	3.18E-13	Up	-0.3462	3.59E-01	-
10	EMIHUDRAFT_439254	NAR, formate/nitrite transporter	K21767//tubulin-specific chaperone D;K01051//pectinesterase [EC:3.1.1.11]+ko00040//Pentose and glucuronate interconversions+ko01100//Metabolic pathways;K11323//histone arginine demethylase JMJD6 [EC:1.14.11.-]	0.9935	5.40E-03	-	1.6957	9.56E-09	Up

11	EMIHU DRAFT_ 76556	NR, nitrate reductase	K10534//nitrate reductase (NAD(P)H) [EC:1.7.1.1 1.7.1.2 1.7.1.3]+ko00910//Nitrogen metabolism+ko01100//Metabolic pathways;K00326//cytochrome-b5 reductase [EC:1.6.2.2]+ko00520//Amino sugar and nucleotide sugar metabolism	0.5704	6.29E-02	-	1.1624	2.54E-07	Up
12	EMIHU DRAFT_ 245144	NR, nitrate reductase	K00326//cytochrome-b5 reductase [EC:1.6.2.2]+ko00520//Amino sugar and nucleotide sugar metabolism;K10534//nitrate reductase (NAD(P)H) [EC:1.7.1.1 1.7.1.2 1.7.1.3]+ko00910//Nitrogen metabolism+ko01100//Metabolic pathways	-0.1764	8.35E-01	-	1.0278	9.45E-03	Up
13	EMIHU DRAFT_ 428968	Fd-Nir, ferredoxin-nitrite reductase	K00366//ferredoxin-nitrite reductase [EC:1.7.7.1]+ko00910//Nitrogen metabolism+ko01100//Metabolic pathways	2.0810	7.09E-05	Up	2.8724	1.30E-09	Up
14	EMIHU DRAFT_ 430293	Fd-Nir, ferredoxin-nitrite reductase	K00366//ferredoxin-nitrite reductase [EC:1.7.7.1]+ko00910//Nitrogen metabolism+ko01100//Metabolic pathways	2.7377	1.15E-07	Up	3.1550	3.68E-10	Up
15	BGI_nov el_G001 601	GS, glutamine synthetase	K01915//glutamine synthetase [EC:6.3.1.2]+ko00250//Alanine, aspartate and glutamate metabolism+ko00910//Nitrogen metabolism+ko00220//Arginine biosynthesis+ko01230//Biosynthesis of amino acids+ko01100//Metabolic pathways+ko00630//Glyoxylate and dicarboxylate metabolism;K14325//RNA-binding protein with serine-rich domain 1+ko03015//mRNA surveillance pathway+ko03013//Nucleocytoplasmic transport;K21952//CASK-interacting protein	-0.1618	8.62E-01	-	1.2634	2.60E-06	Up
16	EMIHU DRAFT_ 437187	GS, glutamine synthetase	K01915//glutamine synthetase [EC:6.3.1.2]+ko00250//Alanine, aspartate and glutamate metabolism+ko00910//Nitrogen metabolism+ko00220//Arginine biosynthesis+ko01230//Biosynthesis of amino acids+ko01100//Metabolic pathways+ko00630//Glyoxylate and dicarboxylate metabolism	1.7321	2.42E-02	Up	3.3299	7.08E-07	Up
17	EMIHU DRAFT_ 72956	GS, glutamine synthetase	K01915//glutamine synthetase [EC:6.3.1.2]+ko00250//Alanine, aspartate and glutamate metabolism+ko00910//Nitrogen metabolism+ko00220//Arginine	0.6242	4.76E-01	-	1.2966	9.14E-03	Up

			biosynthesis+ko01230//Biosynthesis of amino acids+ko01100//Metabolic pathways+ko00630//Glyoxylate and dicarboxylate metabolism						
18	BGI_novel_G001602	GS, glutamine synthetase	K01915//glutamine synthetase [EC:6.3.1.2]+ko00250//Alanine, aspartate and glutamate metabolism+ko00910//Nitrogen metabolism+ko00220//Arginine biosynthesis+ko01230//Biosynthesis of amino acids+ko01100//Metabolic pathways+ko00630//Glyoxylate and dicarboxylate metabolism	0.3507	4.64E-01	-	1.4656	1.97E-08	Up
19	BGI_novel_G003843	GS, glutamine synthetase	K01915//glutamine synthetase [EC:6.3.1.2]+ko00250//Alanine, aspartate and glutamate metabolism+ko00910//Nitrogen metabolism+ko00220//Arginine biosynthesis+ko01230//Biosynthesis of amino acids+ko01100//Metabolic pathways+ko00630//Glyoxylate and dicarboxylate metabolism	2.2982	2.81E-03	Up	4.7596	5.58E-18	Up
20	BGI_novel_G001893	GS, glutamine synthetase	K01915//glutamine synthetase [EC:6.3.1.2]+ko00250//Alanine, aspartate and glutamate metabolism+ko00910//Nitrogen metabolism+ko00220//Arginine biosynthesis+ko01230//Biosynthesis of amino acids+ko01100//Metabolic pathways+ko00630//Glyoxylate and dicarboxylate metabolism	1.3509	2.65E-01	-	2.5353	1.53E-02	Up
21	EMIHUDRAFT_460978	UT, urea transporter	K14306//nuclear pore complex protein Nup62+ko03013//Nucleocytoplasmic transport	2.2423	2.18E-07	Up	1.8545	1.09E-04	Up
22	EMIHUDRAFT_212450	GLT, glutamate synthase	K13171//serine/arginine repetitive matrix protein 1+ko03015//mRNA surveillance pathway+ko03013//Nucleocytoplasmic transport;K13412//calcium-dependent protein kinase [EC:2.7.11.1]+ko04626//Plant-pathogen interaction;K00284//glutamate synthase (ferredoxin) [EC:1.4.7.1]+ko00910//Nitrogen metabolism+ko00630//Glyoxylate and dicarboxylate metabolism	-0.4863	2.41E-01	-	-1.7959	2.36E-08	Down
23	EMIHUDRAFT_225692	GLT, glutamate synthase	K00284//glutamate synthase (ferredoxin) [EC:1.4.7.1]+ko00910//Nitrogen	0.5426	9.33E-02	-	1.5445	4.62E-09	Up

			metabolism+ko00630//Glyoxylate and dicarboxylate metabolism						
24	EMIHU DRAFT_ 467437	GLT, glutamate synthase	K00284//glutamate synthase (ferredoxin) [EC:1.4.7.1]+ko00910//Nitrogen metabolism+ko00630//Glyoxylate and dicarboxylate metabolism;K00264//glutamate synthase (NADH) [EC:1.4.1.14]+ko00250//Alanine, aspartate and glutamate metabolism+ko00910//Nitrogen metabolism+ko01230//Biosynthesis of amino acids+ko01100//Metabolic pathways+ko01110//Biosynthesis of secondary metabolites	0.2349	7.70E-01	-	1.6599	2.28E-05	Up
25	EMIHU DRAFT_ 469498	GLT, glutamate synthase	K00264//glutamate synthase (NADH) [EC:1.4.1.14]+ko00250//Alanine, aspartate and glutamate metabolism+ko00910//Nitrogen metabolism+ko01230//Biosynthesis of amino acids+ko01100//Metabolic pathways+ko01110//Biosynthesis of secondary metabolites	-0.1387	8.42E-01	-	1.2159	7.57E-06	Up
26	EMIHU DRAFT_ 444643	FMD, formamidase	K01455//formamidase [EC:3.5.1.49]+ko00910//Nitrogen metabolism+ko01100//Metabolic pathways+ko00460//Cyanoamino acid metabolism+ko01200//Carbon metabolism+ko00630//Glyoxylate and dicarboxylate metabolism	1.5940	1.09E-01	-	1.9429	3.99E-02	Up
27	EMIHU DRAFT_ 442715	MQO, malate quinone oxioreductas e	K13171//serine/arginine repetitive matrix protein 1+ko03015//mRNA surveillance pathway+ko03013//Nucleocytoplasmic transport	2.8255	2.90E-03	Up	2.0415	8.09E-02	-

[a]Log$_2$ fold change based on RNA-seq data. Each value is the mean from three biological replicates.

[b]Equal to adjusted p-value, change is set at q-value < 0.05 in this study.

Table S9. DEGs involved in ribosome biogenesis and ribosome in PA/P+ and (P+PA)/P+ comparisons.

No.	Gene ID	Description	PA/P+			(P+PA)/P+		
			Log_2 FC[a]	q-value[b]	Regulation	Log_2 FC[a]	q-value[b]	Regulation
1	EMIHUDRAFT_433254	K02937//large subunit ribosomal protein L7e+ko03010//Ribosome	4.2226	5.28E-07	Up	6.2173	5.1E-15	Up
2	EMIHUDRAFT_435105	K02947//small subunit ribosomal protein S10e+ko03010//Ribosome	3.4194	1.09E-16	Up	4.4827	7.08E-25	Up
3	EMIHUDRAFT_52776	K07179//RIO kinase 2 [EC:2.7.11.1]+ko03008//Ribosome biogenesis in eukaryotes	3.2483	1.12E-04	Up	4.3103	5.89E-08	Up
4	EMIHUDRAFT_453845	K14553//U3 small nucleolar RNA-associated protein 18+ko03008//Ribosome biogenesis in eukaryotes	3.1580	1.49E-07	Up	4.7180	7.27E-20	Up
5	EMIHUDRAFT_314847	K14521//N-acetyltransferase 10 [EC:2.3.1.-]+ko03008//Ribosome biogenesis in eukaryotes	3.0175	3.68E-14	Up	3.9560	1.74E-25	Up
6	EMIHUDRAFT_67063	K14568//rRNA small subunit pseudouridine methyltransferase Nep1 [EC:2.1.1.260]+ko03008//Ribosome biogenesis in eukaryotes	3.0135	3.13E-07	Up	3.3792	5E-10	Up
7	EMIHUDRAFT_451580	K03128//transcription initiation factor TFIID subunit 2+ko03022//Basal transcription factors;K14550//U3 small nucleolar RNA-associated protein 10+ko03008//Ribosome biogenesis in eukaryotes	2.9874	2.05E-17	Up	3.2087	2.64E-17	Up
8	EMIHUDRAFT_225493	K18532//adenylate kinase [EC:2.7.4.3]+ko01240//Biosynthesis of cofactors+ko00230//Purine metabolism+ko01100//Metabolic pathways+ko03008//Ribosome biogenesis in eukaryotes+ko01110//Biosynthesis of secondary metabolites	2.9479	8.09E-04	Up	4.5685	1.71E-09	Up
9	EMIHUDRAFT_69773	K02955//small subunit ribosomal protein S14e+ko03010//Ribosome	2.8384	9.23E-13	Up	3.8812	4.71E-23	Up
10	EMIHUDRAFT_228081	K14569//ribosome biogenesis protein BMS1+ko03008//Ribosome biogenesis in eukaryotes	2.8244	5.25E-19	Up	1.8720	2.64E-07	Up
11	EMIHUDRAFT_121580	K14557//U3 small nucleolar RNA-associated protein 6+ko03008//Ribosome biogenesis in eukaryotes;K13171//serine/arginine repetitive matrix protein 1+ko03015//mRNA surveillance pathway+ko03013//Nucleocytoplasmic transport;K13172//serine/arginine repetitive matrix protein 2;K13172//serine/arginine repetitive matrix protein 2	2.8051	4.94E-07	Up	4.1818	1.05E-18	Up

12	EMIHU DRAFT_ 111848	K14569//ribosome biogenesis protein BMS1+ko03008//Ribosome biogenesis in eukaryotes	2.7954	2.29E-24	Up	1.5272	1.66E-06	Up
13	EMIHU DRAFT_ 369951	K07562//60S ribosomal export protein NMD3+ko03013//Nucleocytoplasmic transport+ko03008//Ribosome biogenesis in eukaryotes;K14306//nuclear pore complex protein Nup62+ko03013//Nucleocytoplasmic transport	2.7674	2.87E-06	Up	3.6244	8.73E-11	Up
14	EMIHU DRAFT_ 61494	K14539//large subunit GTPase 1 [EC:3.6.1.-]+ko03008//Ribosome biogenesis in eukaryotes;K02184//formin 2	2.7660	2.95E-08	Up	4.1514	6.56E-20	Up
15	EMIHU DRAFT_ 64122	K14546//U3 small nucleolar RNA-associated protein 5+ko03008//Ribosome biogenesis in eukaryotes;K13172//serine/arginine repetitive matrix protein 2	2.7296	2.85E-12	Up	2.8712	1.54E-13	Up
16	EMIHU DRAFT_ 310412	K14554//U3 small nucleolar RNA-associated protein 21+ko03008//Ribosome biogenesis in eukaryotes	2.7216	9.64E-08	Up	3.8668	5.39E-19	Up
17	EMIHU DRAFT_ 449424	K02947//small subunit ribosomal protein S10e+ko03010//Ribosome	2.7168	5.13E-04	Up	4.2282	4.42E-12	Up
18	EMIHU DRAFT_ 351236	K07562//60S ribosomal export protein NMD3+ko03013//Nucleocytoplasmic transport+ko03008//Ribosome biogenesis in eukaryotes	2.7134	3.94E-09	Up	3.6566	6.65E-18	Up
19	EMIHU DRAFT_ 460715	K02997//small subunit ribosomal protein S9e+ko03010//Ribosome	2.6940	1.80E-13	Up	4.8529	1.58E-38	Up
20	EMIHU DRAFT_ 75659	K14565//nucleolar protein 58+ko03008//Ribosome biogenesis in eukaryotes	2.6910	8.95E-15	Up	4.1175	7.09E-27	Up
21	EMIHU DRAFT_ 350745	K14563//rRNA 2'-O-methyltransferase fibrillarin [EC:2.1.1.-]+ko03008//Ribosome biogenesis in eukaryotes	2.6248	4.01E-14	Up	3.8629	5.99E-23	Up
22	EMIHU DRAFT_ 61745	K02997//small subunit ribosomal protein S9e+ko03010//Ribosome	2.5818	2.05E-09	Up	4.4418	8.96E-23	Up
23	EMIHU DRAFT_ 206438	K14565//nucleolar protein 58+ko03008//Ribosome biogenesis in eukaryotes	2.5733	2.26E-08	Up	3.9067	2.07E-23	Up
24	EMIHU DRAFT_ 105263	K14552//NET1-associated nuclear protein 1 (U3 small nucleolar RNA-associated protein 17)+ko03008//Ribosome biogenesis in eukaryotes;K09566//peptidyl-prolyl isomerase G (cyclophilin G) [EC:5.2.1.8]	2.5685	2.32E-10	Up	3.2199	9.63E-19	Up

25	EMIHU DRAFT_ 317878	K02922//large subunit ribosomal protein L37e+ko03010//Ribosome	2.5574	4.52E-03	Up	4.4978	7.58E-10	Up
26	EMIHU DRAFT_ 309232	K02922//large subunit ribosomal protein L37e+ko03010//Ribosome	2.5574	4.52E-03	Up	4.4978	7.58E-10	Up
27	EMIHU DRAFT_ 457186	K14548//U3 small nucleolar RNA-associated protein 4+ko03008//Ribosome biogenesis in eukaryotes; K13172//serine/arginine repetitive matrix protein 2;K01309//ubiquitin carboxyl-terminal hydrolase MINDY-1/2 [EC:3.4.19.12];K11671//nuclear factor related to kappa-B-binding protein	2.5456	1.08E-06	Up	4.1369	8.13E-21	Up
28	EMIHU DRAFT_ 209137	K14556//U3 small nucleolar RNA-associated protein 12+ko03008//Ribosome biogenesis in eukaryotes	2.4814	1.37E-09	Up	3.5464	1.34E-20	Up
29	EMIHU DRAFT_ 354741	K02991//small subunit ribosomal protein S6e+ko03010//Ribosome	2.4757	1.04E-13	Up	4.4612	5.26E-32	Up
30	EMIHU DRAFT_ 356503	K02998//small subunit ribosomal protein SAe+ko03010//Ribosome	2.4714	1.38E-17	Up	3.7252	1.56E-27	Up
31	EMIHU DRAFT_ 433073	K02973//small subunit ribosomal protein S23e+ko03010//Ribosome	2.4477	1.64E-11	Up	4.3092	1.4E-33	Up
32	BGI_nov el_G000 245	K02997//small subunit ribosomal protein S9e+ko03010//Ribosome	2.3970	4.04E-06	Up	4.1929	4.67E-20	Up
33	EMIHU DRAFT_ 313864	K11131//H/ACA ribonucleoprotein complex subunit 4 [EC:5.4.99.-]+ko03008//Ribosome biogenesis in eukaryotes	2.3901	4.46E-18	Up	3.2627	8.81E-27	Up
34	EMIHU DRAFT_ 433093	K02947//small subunit ribosomal protein S10e+ko03010//Ribosome	2.3874	8.14E-15	Up	4.2106	3.78E-30	Up
35	EMIHU DRAFT_ 216671	K14572//midasin+ko03008//Ribosome biogenesis in eukaryotes;K21952//CASK-interacting protein	2.3642	1.19E-03	Up	3.5726	5.47E-09	Up
36	EMIHU DRAFT_ 453373	K02930//large subunit ribosomal protein L4e+ko03010//Ribosome	2.3640	6.23E-09	Up	4.0636	1.73E-22	Up
37	EMIHU DRAFT_ 103612	K14567//U3 small nucleolar RNA-associated protein 14+ko03008//Ribosome biogenesis in eukaryotes	2.3223	1.57E-02	Up	3.2023	0.000815	Up

38	EMIHU DRAFT_ 216670	K14572//midasin+ko03008//Ribosome biogenesis in eukaryotes	2.2819	1.36E-04	Up	3.6357	9.28E-15	Up
39	EMIHU DRAFT_ 415280	K02985//small subunit ribosomal protein S3e+ko03010//Ribosome	2.2813	2.73E-08	Up	3.7748	1.74E-17	Up
40	EMIHU DRAFT_ 446099	K02937//large subunit ribosomal protein L7e+ko03010//Ribosome	2.2737	1.02E-08	Up	3.9278	6.77E-09	Up
41	EMIHU DRAFT_ 76157	K02930//large subunit ribosomal protein L4e+ko03010//Ribosome	2.2555	5.84E-09	Up	4.5451	1.83E-31	Up
42	EMIHU DRAFT_ 64080	K07178//RIO kinase 1 [EC:2.7.11.1]+ko03008//Ribosome biogenesis in eukaryotes	2.2514	1.51E-05	Up	3.1783	4.34E-13	Up
43	EMIHU DRAFT_ 466218	K06943//nucleolar GTP-binding protein+ko03008//Ribosome biogenesis in eukaryotes	2.2351	2.51E-07	Up	3.3830	3.33E-17	Up
44	EMIHU DRAFT_ 111205	K14557//U3 small nucleolar RNA-associated protein 6+ko03008//Ribosome biogenesis in eukaryotes	2.2317	8.97E-03	Up	3.9457	2.82E-09	Up
45	EMIHU DRAFT_ 445566	K02889//large subunit ribosomal protein L21e+ko03010//Ribosome	2.2058	6.19E-09	Up	4.0136	1.67E-22	Up
46	EMIHU DRAFT_ 310487	K12845//U4/U6 small nuclear ribonucleoprotein SNU13+ko03040//Spliceosome+ko03008//Ribosome biogenesis in eukaryotes	2.1941	3.69E-06	Up	3.9839	3.19E-18	Up
47	EMIHU DRAFT_ 50541	K02873//large subunit ribosomal protein L13e+ko03010//Ribosome	2.1846	4.84E-06	Up	4.5465	1.43E-21	Up
48	EMIHU DRAFT_ 215831	K14552//NET1-associated nuclear protein 1 (U3 small nucleolar RNA-associated protein 17)+ko03008//Ribosome biogenesis in eukaryotes;K09566//peptidyl-prolyl isomerase G (cyclophilin G) [EC:5.2.1.8];K13172//serine/arginine repetitive matrix protein 2	2.1743	6.46E-03	Up	2.8006	2.9E-05	Up
49	EMIHU DRAFT_ 255728	K02949//small subunit ribosomal protein S11e+ko03010//Ribosome	2.1733	8.31E-03	Up	3.1948	6.06E-07	Up
50	EMIHU DRAFT_ 200700	K02938//large subunit ribosomal protein L8e+ko03010//Ribosome	2.1663	9.39E-11	Up	4.0315	5.41E-26	Up
51	EMIHU DRAFT_ 70587	K02925//large subunit ribosomal protein L3e+ko03010//Ribosome	2.1604	8.16E-05	Up	4.0863	3.18E-08	Up

52	EMIHU DRAFT_ 223278	K02957//small subunit ribosomal protein S15Ae+ko03010//Ribosome	2.1434	8.29E-06	Up	4.3016	3.25E-21	Up
53	EMIHU DRAFT_ 253403	K02941//large subunit ribosomal protein LP0+ko03010//Ribosome	2.1403	3.64E-04	Up	3.9358	6.13E-07	Up
54	EMIHU DRAFT_ 197835	K14538//nuclear GTP-binding protein+ko03008//Ribosome biogenesis in eukaryotes	2.1230	6.23E-07	Up	3.7264	1.86E-29	Up
55	EMIHU DRAFT_ 467193	K14558//periodic tryptophan protein 2+ko03008//Ribosome biogenesis in eukaryotes	2.1177	1.86E-24	Up	1.2080	5.46E-08	Up
56	EMIHU DRAFT_ 51731	K14537//nuclear GTP-binding protein+ko03008//Ribosome biogenesis in eukaryotes	2.1139	1.65E-05	Up	3.1435	1.05E-12	Up
57	EMIHU DRAFT_ 425447	K02978//small subunit ribosomal protein S27e+ko03010//Ribosome	2.0453	2.36E-06	Up	3.6397	4.49E-18	Up
58	EMIHU DRAFT_ 63451	K11108//RNA 3'-terminal phosphate cyclase-like protein+ko03008//Ribosome biogenesis in eukaryotes	2.0448	1.40E-03	Up	2.9667	1.07E-09	Up
59	EMIHU DRAFT_ 443828	K02964//small subunit ribosomal protein S18e+ko03010//Ribosome	2.0405	9.52E-09	Up	4.0564	4E-23	Up
60	EMIHU DRAFT_ 433854	K02883//large subunit ribosomal protein L18e+ko03010//Ribosome	2.0322	2.77E-03	Up	4.0043	1.57E-09	Up
61	EMIHU DRAFT_ 415433	K02873//large subunit ribosomal protein L13e+ko03010//Ribosome	2.0031	3.14E-07	Up	4.3504	4.34E-27	Up
62	EMIHU DRAFT_ 433492	K02906//large subunit ribosomal protein L3+ko03010//Ribosome	2.0012	7.76E-07	Up	0.9422	0.036672	-
63	EMIHU DRAFT_ 358111	K02951//small subunit ribosomal protein S12e+ko03010//Ribosome	1.9945	4.15E-05	Up	3.6256	4.08E-07	Up
64	EMIHU DRAFT_ 123268	K02962//small subunit ribosomal protein S17e+ko03010//Ribosome	1.9943	6.95E-03	Up	4.8125	7.67E-18	Up
65	EMIHU DRAFT_ 455347	K20553//5'-3' exoribonuclease 4 [EC:3.1.13.-]+ko04016//MAPK signaling pathway - plant;K12619//5'-3' exoribonuclease 2 [EC:3.1.13.-]+ko03018//RNA degradation+ko03008//Ribosome biogenesis in eukaryotes;K12618//5'-3' exoribonuclease 1	1.9821	2.38E-02	Up	4.0993	5.07E-12	Up

		[EC:3.1.13.-]+ko03018//RNA degradation+ko03008//Ribosome biogenesis in eukaryotes						
66	EMIHU DRAFT_ 358841	K02942//large subunit ribosomal protein LP1+ko03010//Ribosome	1.9559	1.39E-06	Up	4.0662	1.82E-22	Up
67	EMIHU DRAFT_ 444406	K02962//small subunit ribosomal protein S17e+ko03010//Ribosome	1.9552	1.36E-06	Up	3.8219	1.54E-08	Up
68	EMIHU DRAFT_ 456254	K02941//large subunit ribosomal protein LP0+ko03010//Ribosome	1.9388	2.82E-06	Up	3.7708	1.17E-08	Up
69	EMIHU DRAFT_ 414178	K02872//large subunit ribosomal protein L13Ae+ko03010//Ribosome	1.9366	2.04E-05	Up	3.6804	5.78E-08	Up
70	EMIHU DRAFT_ 76734	K02971//small subunit ribosomal protein S21e+ko03010//Ribosome	1.9297	6.46E-03	Up	3.8447	1.22E-11	Up
71	EMIHU DRAFT_ 434886	K14564//nucleolar protein 56+ko03008//Ribosome biogenesis in eukaryotes	1.8977	1.19E-10	Up	2.4501	7.09E-16	Up
72	EMIHU DRAFT_ 205063	K14559//U3 small nucleolar RNA-associated protein MPP10+ko03008//Ribosome biogenesis in eukaryotes;K11323//histone arginine demethylase JMJD6 [EC:1.14.11.-]	1.8732	2.42E-03	Up	2.4355	7.68E-08	Up
73	EMIHU DRAFT_ 437374	K02989//small subunit ribosomal protein S5e+ko03010//Ribosome	1.8498	1.96E-06	Up	3.5985	7.59E-17	Up
74	EMIHU DRAFT_ 241354	K14548//U3 small nucleolar RNA-associated protein 4+ko03008//Ribosome biogenesis in eukaryotes	1.8012	1.00E-01	Up	4.0936	6.18E-07	Up
75	EMIHU DRAFT_ 316252	K02984//small subunit ribosomal protein S3Ae+ko03010//Ribosome	1.7196	2.16E-04	Up	3.9788	1.48E-17	Up
76	EMIHU DRAFT_ 74335	K02979//small subunit ribosomal protein S28e+ko03010//Ribosome	1.6897	3.11E-03	Up	3.1425	4.57E-05	Up
77	EMIHU DRAFT_ 418142	K02894//large subunit ribosomal protein L23e+ko03010//Ribosome	1.6860	2.23E-04	Up	3.5828	1.08E-14	Up
78	EMIHU DRAFT_ 433471	K02896//large subunit ribosomal protein L24e+ko03010//Ribosome	1.6841	4.39E-05	Up	3.1464	3.95E-15	Up

79	EMIHU DRAFT_ 420028	K02882//large subunit ribosomal protein L18Ae+ko03010//Ribosome	1.6828	5.22E-03	Up	3.7145	5.12E-13	Up
80	EMIHU DRAFT_ 55884	K02987//small subunit ribosomal protein S4e+ko03010//Ribosome	1.6630	1.66E-04	Up	3.4116	6.93E-07	Up
81	EMIHU DRAFT_ 432120	K02943//large subunit ribosomal protein LP2+ko03010//Ribosome	1.6194	1.27E-05	Up	3.2240	2.35E-17	Up
82	EMIHU DRAFT_ 448353	K14545//ribosomal RNA-processing protein 7+ko03008//Ribosome biogenesis in eukaryotes	1.5977	1.75E-04	Up	2.2813	4.58E-09	Up
83	EMIHU DRAFT_ 75634	K14571//ribosome biogenesis ATPase+ko03008//Ribosome biogenesis in eukaryotes	1.5975	1.95E-02	Up	2.2581	2.93E-05	Up
84	EMIHU DRAFT_ 351077	K02925//large subunit ribosomal protein L3e+ko03010//Ribosome	1.5857	7.61E-02	Up	3.2523	7.1E-05	Up
85	EMIHU DRAFT_ 434089	K02973//small subunit ribosomal protein S23e+ko03010//Ribosome	1.5822	6.84E-04	Up	3.2087	3.21E-06	Up
86	EMIHU DRAFT_ 438398	K02984//small subunit ribosomal protein S3Ae+ko03010//Ribosome	1.5821	1.99E-04	Up	3.5514	1.83E-07	Up
87	EMIHU DRAFT_ 442529	K02920//large subunit ribosomal protein L36e+ko03010//Ribosome	1.5773	2.94E-04	Up	3.3934	8.97E-07	Up
88	EMIHU DRAFT_ 439486	K02966//small subunit ribosomal protein S19e+ko03010//Ribosome	1.5749	2.28E-05	Up	3.3842	9.33E-17	Up
89	EMIHU DRAFT_ 78524	K02936//large subunit ribosomal protein L7Ae+ko03010//Ribosome	1.5445	3.83E-02	Up	3.4497	3.69E-06	Up
90	EMIHU DRAFT_ 450720	K02976//small subunit ribosomal protein S26e+ko03010//Ribosome	1.5320	2.23E-08	Up	2.8634	1.95E-15	Up
91	EMIHU DRAFT_ 243741	K02880//large subunit ribosomal protein L17e+ko03010//Ribosome	1.5257	2.31E-02	Up	4.2977	1.18E-18	Up
92	EMIHU DRAFT_ 438529	K02974//small subunit ribosomal protein S24e+ko03010//Ribosome;K15174//RNA polymerase II-associated factor 1;K02184//formin 2	1.5164	1.69E-03	Up	3.9627	5.78E-19	Up

93	EMIHU DRAFT_ 68698	K02924//large subunit ribosomal protein L39e+ko03010//Ribosome;K10747//DNA ligase 1 [EC:6.5.1.1 6.5.1.6 6.5.1.7]+ko03420//Nucleotide excision repair+ko03030//DNA replication+ko03410//Base excision repair+ko03430//Mismatch repair	1.5111	1.75E-03	Up	3.2161	1.5E-14	Up
94	EMIHU DRAFT_ 415402	K02995//small subunit ribosomal protein S8e+ko03010//Ribosome	1.5040	3.07E-04	Up	3.3797	1.81E-07	Up
95	EMIHU DRAFT_ 432486	K02910//large subunit ribosomal protein L31e+ko03010//Ribosome	1.4978	7.45E-04	Up	3.3058	9.29E-07	Up
96	EMIHU DRAFT_ 315158	K02882//large subunit ribosomal protein L18Ae+ko03010//Ribosome	1.4952	4.70E-02	Up	3.0693	2.79E-05	Up
97	EMIHU DRAFT_ 445650	K02974//small subunit ribosomal protein S24e+ko03010//Ribosome	1.4882	2.06E-03	Up	3.9687	4.48E-19	Up
98	EMIHU DRAFT_ 446643	K02974//small subunit ribosomal protein S24e+ko03010//Ribosome	1.4882	2.06E-03	Up	4.0374	3E-19	Up
99	EMIHU DRAFT_ 441392	K02875//large subunit ribosomal protein L14e+ko03010//Ribosome	1.4869	7.60E-02	Up	3.5680	2.51E-06	Up
100	EMIHU DRAFT_ 240177	K02981//small subunit ribosomal protein S2e+ko03010//Ribosome	1.4580	4.13E-02	Up	3.0597	0.000243	Up
101	EMIHU DRAFT_ 442198	K02995//small subunit ribosomal protein S8e+ko03010//Ribosome	1.4317	1.25E-03	Up	3.2103	7.2E-14	Up
102	EMIHU DRAFT_ 433742	K02901//large subunit ribosomal protein L27e+ko03010//Ribosome	1.4229	1.82E-06	Up	2.7008	2.32E-13	Up
103	EMIHU DRAFT_ 308697	K02918//large subunit ribosomal protein L35e+ko03010//Ribosome	1.4111	4.46E-03	Up	3.4693	1.78E-13	Up
104	EMIHU DRAFT_ 362722	K02921//large subunit ribosomal protein L37Ae+ko03010//Ribosome	1.4024	4.27E-03	Up	3.4080	6.5E-07	Up
105	EMIHU DRAFT_ 221723	K11130//H/ACA ribonucleoprotein complex subunit 3+ko03008//Ribosome biogenesis in eukaryotes	1.3924	1.58E-02	Up	1.9977	6.52E-05	Up
106	EMIHU DRAFT_ 68649	K14561//U3 small nucleolar ribonucleoprotein protein IMP4+ko03008//Ribosome biogenesis in eukaryotes	1.3894	2.42E-02	Up	2.9840	7.37E-13	Up

107	EMIHU DRAFT_ 427548	K02903//large subunit ribosomal protein L28e+ko03010//Ribosome	1.3784	2.03E-04	Up	3.1504	2.86E-14	Up
108	EMIHU DRAFT_ 60889	K02870//large subunit ribosomal protein L12e+ko03010//Ribosome	1.3683	6.18E-03	Up	3.0620	1.18E-10	Up
109	EMIHU DRAFT_ 426384	K02908//large subunit ribosomal protein L30e+ko03010//Ribosome	1.3485	5.15E-07	Up	2.4259	6.05E-12	Up
110	EMIHU DRAFT_ 310388	K02958//small subunit ribosomal protein S15e+ko03010//Ribosome	1.3356	2.60E-03	Up	3.2203	9.58E-14	Up
111	EMIHU DRAFT_ 356928	K02923//large subunit ribosomal protein L38e+ko03010//Ribosome	1.3224	2.78E-03	Up	3.1957	2.35E-06	Up
112	EMIHU DRAFT_ 66337	K14560//U3 small nucleolar ribonucleoprotein protein IMP3+ko03008//Ribosome biogenesis in eukaryotes	1.3142	1.72E-01	Up	2.6281	2.65E-05	Up
113	EMIHU DRAFT_ 214002	K14539//large subunit GTPase 1 [EC:3.6.1.-]+ko03008//Ribosome biogenesis in eukaryotes	1.3011	2.97E-01	Up	2.5144	0.008103	Up
114	EMIHU DRAFT_ 445032	K02868//large subunit ribosomal protein L11e+ko03010//Ribosome	1.2948	9.45E-02	Up	3.2540	6.25E-06	Up
115	EMIHU DRAFT_ 439611	K02867//large subunit ribosomal protein L11+ko03010//Ribosome	1.2834	2.68E-01	Up	3.2610	0.001953	Up
116	EMIHU DRAFT_ 96871	K11593//eukaryotic translation initiation factor 2C;K14563//rRNA 2'-O-methyltransferase fibrillarin [EC:2.1.1.-]+ko03008//Ribosome biogenesis in eukaryotes	1.2639	5.84E-06	Up	0.6145	0.049546	-
117	EMIHU DRAFT_ 121169	K14566//U3 small nucleolar RNA-associated protein 24+ko03008//Ribosome biogenesis in eukaryotes	1.2451	7.59E-02	Up	3.0038	4.14E-11	Up
118	EMIHU DRAFT_ 414120	K02953//small subunit ribosomal protein S13e+ko03010//Ribosome	1.2176	1.49E-01	Up	3.3700	2.41E-06	Up
119	EMIHU DRAFT_ 417676	K02898//large subunit ribosomal protein L26e+ko03010//Ribosome	1.2107	1.47E-01	Up	3.2935	5.01E-06	Up
120	EMIHU DRAFT_ 448590	K02949//small subunit ribosomal protein S11e+ko03010//Ribosome	1.2105	1.34E-01	Up	3.1078	4.05E-05	Up

121	EMIHU DRAFT_ 58086	K02896//large subunit ribosomal protein L24e+ko03010//Ribosome	1.2035	2.21E-05	Up	3.2946	1.73E-21	Up
122	EMIHU DRAFT_ 455093	K11129//H/ACA ribonucleoprotein complex subunit 2+ko03008//Ribosome biogenesis in eukaryotes	1.18065	0.037409	Up	3.3348	2.26E-14	Up
123	EMIHU DRAFT_ 77738	K14549//U3 small nucleolar RNA-associated protein 15+ko03008//Ribosome biogenesis in eukaryotes	1.18059 69	0.068056	Up	1.3330	0.017147	Up
124	EMIHU DRAFT_ 59431	K02893//large subunit ribosomal protein L23Ae+ko03010//Ribosome	1.16662 1	0.066026	Up	3.1463	1.64E-09	Up
125	EMIHU DRAFT_ 418918	K14536//ribosome assembly protein 1 [EC:3.6.5.-]+ko03008//Ribosome biogenesis in eukaryotes	1.15981 12	0.076627	Up	2.7340	2.58E-10	Up
126	EMIHU DRAFT_ 75738	K18532//adenylate kinase [EC:2.7.4.3]+ko01240//Biosynthesis of cofactors+ko00230//Purine metabolism+ko01100//Metabolic pathways+ko03008//Ribosome biogenesis in eukaryotes+ko01110//Biosynthesis of secondary metabolites	1.13259 51	0.221511	Up	2.6687	2.87E-06	Up
127	EMIHU DRAFT_ 351999	K02912//large subunit ribosomal protein L32e+ko03010//Ribosome	1.13161 97	0.011298	Up	3.1843	4.58E-14	Up
128	EMIHU DRAFT_ 352002	K02912//large subunit ribosomal protein L32e+ko03010//Ribosome	1.13161 97	0.011298	Up	3.1843	4.58E-14	Up
129	EMIHU DRAFT_ 429999	K02917//large subunit ribosomal protein L35Ae+ko03010//Ribosome	1.12373 93	0.230185	Up	3.2217	2.43E-06	Up
130	EMIHU DRAFT_ 77965	K02913//large subunit ribosomal protein L33+ko03010//Ribosome;K10747//DNA ligase 1 [EC:6.5.1.1 6.5.1.6 6.5.1.7]+ko03420//Nucleotide excision repair+ko03030//DNA replication+ko03410//Base excision repair+ko03430//Mismatch repair	1.08844 81	3.38E-05	Up	- 0.1525	0.796573	-
131	EMIHU DRAFT_ 434648	K02932//large subunit ribosomal protein L5e+ko03010//Ribosome	1.07926 49	0.265348	Up	3.1621	6.84E-05	Up
132	EMIHU DRAFT_ 415128	K02872//large subunit ribosomal protein L13Ae+ko03010//Ribosome	1.06674 04	0.43543	Up	2.3624	0.037367	Up
133	EMIHU DRAFT_ 218353	K11128//H/ACA ribonucleoprotein complex subunit 1+ko03008//Ribosome biogenesis in eukaryotes	1.05714 78	0.170863	Up	2.8122	7.28E-10	Up

134	EMIHU DRAFT_ 207319	K14570//RNA exonuclease [EC:3.1.-.-]+ko03008//Ribosome biogenesis in eukaryotes;K16302//metal transporter CNNM;K01309//ubiquitin carboxyl-terminal hydrolase MINDY-1/2 [EC:3.4.19.12]	1.03171 02	0.020256	Up	1.7242	3.09E-07	Up
135	EMIHU DRAFT_ 368254	K02995//small subunit ribosomal protein S8e+ko03010//Ribosome;K13172//serine/arginine repetitive matrix protein 2	1.00105 49	0.00751	Up	2.8122	3.14E-16	Up
136	EMIHU DRAFT_ 436239	K02889//large subunit ribosomal protein L21e+ko03010//Ribosome	0.98615 97	0.425828	-	2.3047	0.044803	Up
137	EMIHU DRAFT_ 60285	K02975//small subunit ribosomal protein S25e+ko03010//Ribosome	0.98526 16	0.070678	-	3.6729	6.44E-17	Up
138	EMIHU DRAFT_ 60245	K02975//small subunit ribosomal protein S25e+ko03010//Ribosome	0.98526 16	0.070678	-	3.6729	6.44E-17	Up
139	EMIHU DRAFT_ 632209	K07936//GTP-binding nuclear protein Ran+ko03013//Nucleocytoplasmic transport+ko03008//Ribosome biogenesis in eukaryotes	0.97033 55	0.000584	-	1.5810	9.03E-08	Up
140	EMIHU DRAFT_ 427386	K02891//large subunit ribosomal protein L22e+ko03010//Ribosome	0.92783 56	0.015567	-	2.6237	4.07E-12	Up
141	EMIHU DRAFT_ 444698	K02155//V-type H+-transporting ATPase 16kDa proteolipid subunit+ko04145//Phagosome+ko01100//Metabolic pathways+ko00190//Oxidative phosphorylation;K14573//nucleolar protein 4+ko03008//Ribosome biogenesis in eukaryotes	0.92433 1	0.000632	-	1.5602	4.28E-10	Up
142	EMIHU DRAFT_ 414756	K02969//small subunit ribosomal protein S20e+ko03010//Ribosome	0.84014 98	0.325829	-	2.9252	2.27E-05	Up
143	EMIHU DRAFT_ 434266	K02977//ubiquitin-small subunit ribosomal protein S27Ae+ko04120//Ubiquitin mediated proteolysis+ko03010//Ribosome	0.81594 47	0.415106	-	3.0858	5.68E-05	Up
144	BGI_nov el_G003 389	K02863//large subunit ribosomal protein L1+ko03010//Ribosome	0.81157 7	0.449739	-	1.8331	0.002638	Up
145	EMIHU DRAFT_ 414037	K02865//large subunit ribosomal protein L10Ae+ko03010//Ribosome	0.78437 66	0.425162	-	2.9492	0.00011	Up
146	EMIHU DRAFT_ 413744	K02865//large subunit ribosomal protein L10Ae+ko03010//Ribosome	0.78437 66	0.425162	-	2.9492	0.00011	Up
147	EMIHU DRAFT_ 632210	K07936//GTP-binding nuclear protein Ran+ko03013//Nucleocytoplasmic transport+ko03008//Ribosome biogenesis in eukaryotes	0.75933 2	0.284889	-	1.5735	0.001421	Up

148	EMIHU DRAFT_ 456021	K02900//large subunit ribosomal protein L27Ae+ko03010//Ribosome	0.72433 4	0.509575	-	2.9929	8.63E-05	Up
149	EMIHU DRAFT_ 61989	K02983//small subunit ribosomal protein S30e+ko03010//Ribosome	0.70011 64	0.32333	-	2.1386	2.92E-06	Up
150	EMIHU DRAFT_ 455468	K02968//small subunit ribosomal protein S20+ko03010//Ribosome	0.68648 97	0.086835	-	1.1660	0.001023	Up
151	EMIHU DRAFT_ 368288	K02898//large subunit ribosomal protein L26e+ko03010//Ribosome	0.68455 52	0.670726	-	2.2907	0.018082	Up
152	EMIHU DRAFT_ 421555	K14536//ribosome assembly protein 1 [EC:3.6.5.-]+ko03008//Ribosome biogenesis in eukaryotes	0.67820 19	0.422861	-	2.0870	1.02E-06	Up
153	EMIHU DRAFT_ 50610	K02932//large subunit ribosomal protein L5e+ko03010//Ribosome	0.66149 82	0.622174	-	2.4447	0.000344	Up
154	EMIHU DRAFT_ 468608	K02863//large subunit ribosomal protein L1+ko03010//Ribosome	0.66113 83	0.12568	-	1.8868	1.87E-08	Up
155	EMIHU DRAFT_ 351730	K02940//large subunit ribosomal protein L9e+ko03010//Ribosome	0.65622 04	0.020036	-	1.2882	1.02E-05	Up
156	EMIHU DRAFT_ 195676	K13172//serine/arginine repetitive matrix protein 2;K14325//RNA-binding protein with serine-rich domain 1+ko03015//mRNA surveillance pathway+ko03013//Nucleocytoplasmic transport;K00430//peroxidase [EC:1.11.1.7]+ko01100//Metabolic pathways+ko01110//Biosynthesis of secondary metabolites+ko00940//Phenylpropanoid biosynthesis; K14575//AAA family ATPase+ko03008//Ribosome biogenesis in eukaryotes	0.65148 49	0.126886	-	1.9536	2.82E-13	Up
157	EMIHU DRAFT_ 446072	K02866//large subunit ribosomal protein L10e+ko03010//Ribosome	0.63542 58	0.043873	-	1.1591	0.000915	Up
158	EMIHU DRAFT_ 69198	K02945//small subunit ribosomal protein S1+ko03010//Ribosome	0.60860 32	0.107276	-	1.6372	7.88E-08	Up
159	EMIHU DRAFT_ 62761	K02905//large subunit ribosomal protein L29e+ko03010//Ribosome	0.55111 54	0.596569	-	2.2667	1.26E-05	Up
160	EMIHU DRAFT_ 70861	K02895//large subunit ribosomal protein L24+ko03010//Ribosome	0.50201 02	0.345325	-	1.5370	5E-05	Up

161	EMIHU DRAFT_ 416466	K02891//large subunit ribosomal protein L22e+ko03010//Ribosome	0.48906 98	0.644983	-	2.2523	3.2E-07	Up
162	EMIHU DRAFT_ 415031	K02935//large subunit ribosomal protein L7/L12+ko03010//Ribosome	0.46784 72	0.357026	-	1.7291	5.57E-06	Up
163	EMIHU DRAFT_ 424396	K02867//large subunit ribosomal protein L11+ko03010//Ribosome	0.45223 87	0.358128	-	1.2194	0.000487	Up
164	EMIHU DRAFT_ 465926	K01689//enolase [EC:4.2.1.11]+ko00010//Glycolysis / Gluconeogenesis+ko01230//Biosynthesis of amino acids+ko03018//RNA degradation+ko01100//Metabolic pathways+ko01110//Biosynthesis of secondary metabolites+ko01200//Carbon metabolism;K14564//nucleolar protein 56+ko03008//Ribosome biogenesis in eukaryotes	0.43198 35	0.181483	-	1.3617	4.41E-08	Up
165	EMIHU DRAFT_ 455557	K02935//large subunit ribosomal protein L7/L12+ko03010//Ribosome	0.42092 21	0.423756	-	1.7987	3.13E-09	Up
166	EMIHU DRAFT_ 73674	K11129//H/ACA ribonucleoprotein complex subunit 2+ko03008//Ribosome biogenesis in eukaryotes	0.40944 88	0.813403	-	2.3032	0.001585	Up
167	EMIHU DRAFT_ 197224	K11593//eukaryotic translation initiation factor 2C;K01051//pectinesterase [EC:3.1.1.11]+ko00040//Pentose and glucuronate interconversions+ko01100//Metabolic pathways;K00430//peroxidase [EC:1.11.1.7]+ko01100//Metabolic pathways+ko01110//Biosynthesis of secondary metabolites+ko00940//Phenylpropanoid biosynthesis;K14567//U3 small nucleolar RNA-associated protein 14+ko03008//Ribosome biogenesis in eukaryotes;K02184//formin 2;K01309//ubiquitin carboxyl-terminal hydrolase MINDY-1/2 [EC:3.4.19.12];K21952//CASK-interacting protein	0.37865 42	0.830371	-	1.9468	0.00326	Up
168	EMIHU DRAFT_ 443529	K02864//large subunit ribosomal protein L10+ko03010//Ribosome	0.37750 1	0.371395	-	1.1904	8.22E-05	Up
169	EMIHU DRAFT_ 457670	K02876//large subunit ribosomal protein L15+ko03010//Ribosome	0.33774 95	0.458455	-	1.3266	1.72E-06	Up
170	EMIHU DRAFT_ 250523	K14994//solute carrier family 38 (sodium-coupled neutral amino acid transporter), member 7/8;K13412//calcium-dependent protein kinase [EC:2.7.11.1]+ko04626//Plant-pathogen interaction;K14563//rRNA 2'-O-methyltransferase fibrillarin [EC:2.1.1.-]+ko03008//Ribosome biogenesis in eukaryotes;K00430//peroxidase [EC:1.11.1.7]+ko01100//Metabolic pathways+ko01110//Biosynthesis of secondary metabolites+ko00940//Phenylpropanoid biosynthesis;K01180//endo-1,3(4)-beta-glucanase [EC:3.2.1.6];K21952//CASK-interacting protein	0.25971 15	0.860774	-	2.2660	0.000161	Up

171	EMIHUDRAFT_455108	K12618//5'-3' exoribonuclease 1 [EC:3.1.13.-]+ko03018//RNA degradation+ko03008//Ribosome biogenesis in eukaryotes;K20553//5'-3' exoribonuclease 4 [EC:3.1.13.-]+ko04016//MAPK signaling pathway - plant	0.1833916	0.761453	-	1.2402	1.77E-07	Up
172	EMIHUDRAFT_462610	K13172//serine/arginine repetitive matrix protein 2;K13171//serine/arginine repetitive matrix protein 1+ko03015//mRNA surveillance pathway+ko03013//Nucleocytoplasmic transport;K14563//rRNA 2'-O-methyltransferase fibrillarin [EC:2.1.1.-]+ko03008//Ribosome biogenesis in eukaryotes	0.1615392	0.732938	-	1.6655	1.2E-08	Up
173	EMIHUDRAFT_434457	K02881//large subunit ribosomal protein L18+ko03010//Ribosome	0.1224529	0.850534	-	1.0493	0.004744	Up
174	EMIHUDRAFT_440718	K02988//small subunit ribosomal protein S5+ko03010//Ribosome;K13171//serine/arginine repetitive matrix protein 1+ko03015//mRNA surveillance pathway+ko03013//Nucleocytoplasmic transport	-0.217923	0.731818	-	1.0499	0.000214	Up
175	EMIHUDRAFT_77213	K02885//large subunit ribosomal protein L19e+ko03010//Ribosome	-0.225623	0.900888	-	1.8205	0.039766	Up
176	EMIHUDRAFT_228102	K02902//large subunit ribosomal protein L28+ko03010//Ribosome	-0.270264	0.842174	-	1.2766	0.022448	Up
177	EMIHUDRAFT_202496	K06943//nucleolar GTP-binding protein+ko03008//Ribosome biogenesis in eukaryotes	-0.288324	0.846619	-	-1.9706	0.029561	Down
178	EMIHUDRAFT_450250	K14544//U3 small nucleolar RNA-associated protein 22+ko03008//Ribosome biogenesis in eukaryotes;K13711//phosphatidylinositol 4-kinase type 2 [EC:2.7.1.67]+ko01100//Metabolic pathways+ko04070//Phosphatidylinositol signaling system+ko00562//Inositol phosphate metabolism;K09566//peptidyl-prolyl isomerase G (cyclophilin G) [EC:5.2.1.8];K13172//serine/arginine repetitive matrix protein 2;K09566//peptidyl-prolyl isomerase G (cyclophilin G) [EC:5.2.1.8];K13172//serine/arginine repetitive matrix protein 2;K13172//serine/arginine repetitive matrix protein 2;K14544//U3 small nucleolar RNA-associated protein 22+ko03008//Ribosome biogenesis in eukaryotes	-0.481059	0.064218	-	-1.1990	2.87E-07	Down
179	EMIHUDRAFT_455634	K13711//phosphatidylinositol 4-kinase type 2 [EC:2.7.1.67]+ko01100//Metabolic pathways+ko04070//Phosphatidylinositol signaling system+ko00562//Inositol phosphate metabolism;K14544//U3 small nucleolar RNA-associated protein 22+ko03008//Ribosome biogenesis in	-0.677944	0.00498	-	-1.2379	1.65E-07	Down

		eukaryotes;K09566//peptidyl-prolyl isomerase G (cyclophilin G) [EC:5.2.1.8];K13172//serine/arginine repetitive matrix protein 2						
180	EMIHU DRAFT_ 354743	K02887//large subunit ribosomal protein L20+ko03010//Ribosome	-0.705118	0.024584	-	-1.0966	7.06E-05	Down
181	EMIHU DRAFT_ 258184	K00252//glutaryl-CoA dehydrogenase [EC:1.3.8.6]+ko00310//Lysine degradation+ko00380//Tryptophan metabolism+ko01100//Metabolic pathways+ko01110//Biosynthesis of secondary metabolites+ko00071//Fatty acid degradation;K14563//rRNA 2'-O-methyltransferase fibrillarin [EC:2.1.1.-]+ko03008//Ribosome biogenesis in eukaryotes;K00253//isovaleryl-CoA dehydrogenase [EC:1.3.8.4]+ko00280//Valine, leucine and isoleucine degradation+ko01100//Metabolic pathways	-1.042474	0.008069	Down	-1.0832	0.000868	Down
182	EMIHU DRAFT_ 45174	K12619//5'-3' exoribonuclease 2 [EC:3.1.13.-]+ko03018//RNA degradation+ko03008//Ribosome biogenesis in eukaryotes;K20553//5'-3' exoribonuclease 4 [EC:3.1.13.-]+ko04016//MAPK signaling pathway - plant;K13172//serine/arginine repetitive matrix protein 2;K03128//transcription initiation factor TFIID subunit 2+ko03022//Basal transcription factors	-1.070809	0.049846	Down	-0.5390	0.217294	-
183	EMIHU DRAFT_ 470187	K03254//translation initiation factor 3 subunit A;K14567//U3 small nucleolar RNA-associated protein 14+ko03008//Ribosome biogenesis in eukaryotes	-1.071516	0.018465	Down	-0.0926	0.865131	-
184	BGI_nov el_G000 086	K12618//5'-3' exoribonuclease 1 [EC:3.1.13.-]+ko03018//RNA degradation+ko03008//Ribosome biogenesis in eukaryotes;K20553//5'-3' exoribonuclease 4 [EC:3.1.13.-]+ko04016//MAPK signaling pathway - plant	-1.10352	0.099483	Down	-1.6822	0.005068	Down
185	EMIHU DRAFT_ 252069	K02993//small subunit ribosomal protein S7e+ko03010//Ribosome;K11323//histone arginine demethylase JMJD6 [EC:1.14.11.-]	-1.312782	0.018237	Down	-0.2193	0.739947	-
186	EMIHU DRAFT_ 448662	K12618//5'-3' exoribonuclease 1 [EC:3.1.13.-]+ko03018//RNA degradation+ko03008//Ribosome biogenesis in eukaryotes;K14317//nuclear pore complex protein Nup214+ko03013//Nucleocytoplasmic transport	-1.436221	0.17083	Down	-2.2786	0.015869	Down
187	EMIHU DRAFT_ 237027	K12618//5'-3' exoribonuclease 1 [EC:3.1.13.-]+ko03018//RNA degradation+ko03008//Ribosome biogenesis in eukaryotes;K20553//5'-3' exoribonuclease 4 [EC:3.1.13.-]+ko04016//MAPK signaling pathway - plant;K14317//nuclear pore complex protein Nup214+ko03013//Nucleocytoplasmic transport;K03128//transcription initiation factor TFIID subunit 2+ko03022//Basal transcription factors	-1.639571	1.28E-09	Down	-1.6392	1.35E-09	Down

[a]Log_2 fold change based on RNA-seq data. Each value is the mean from three biological replicates.

[b]Equal to adjusted p-value, change is set at q-value < 0.05 in this study.

Table S10. DEGs involved in glycolysis in PA/P+ and (P+PA)/P+ comparisons.

No.	Gene ID	Gene name	PA/P+			(P+PA)/P+		
			Log_2 FC[a]	q-value[b]	Regulation	Log_2 FC[a]	q-value[b]	Regulation
1	EMIHUDRAFT_454319	GCK, glucokinase	0.1483	8.30E-01	-	1.3549	1.22E-06	Up
2	EMIHUDRAFT_464599	GCK, glucokinase	1.2624	3.24E-01	-	4.6626	5.29E-09	Up
3	EMIHUDRAFT_446110	GPI, glucose-6-phosphate isomerase	0.6660	6.18E-01	-	3.6594	8.73E-11	Up
4	EMIHUDRAFT_558335	GPI, glucose-6-phosphate isomerase	0.3886	6.95E-01	-	2.9663	3.75E-09	Up
5	EMIHUDRAFT_436550	FBA, fructose-bisphosphate aldolase	1.1558	5.41E-03	Up	2.0204	2.42E-08	Up
6	EMIHUDRAFT_308857	FBA, fructose-bisphosphate aldolase	1.2062	2.58E-01	-	4.7148	1.55E-18	Up
7	EMIHUDRAFT_418341	FBA, fructose-bisphosphate aldolase	0.2923	4.65E-01	-	1.3778	4.71E-08	Up
8	EMIHUDRAFT_74095	TIM, triosephosphate isomerase	1.4519	3.78E-03	Up	3.4393	4.41E-18	Up
9	EMIHUDRAFT_68485	TIM, triosephosphate isomerase	1.4519	3.78E-03	Up	3.43928	4.41E-18	Up
10	EMIHUDRAFT_446527	TIM, triosephosphate isomerase	1.0777	8.49E-03	Up	2.8792	5.00E-12	Up
11	EMIHUDRAFT_438339	TIM, triosephosphate isomerase	0.9824	2.84E-02	-	2.8348	2.00E-12	Up
12	EMIHUDRAFT_437834	GAPDH, glyceraldehyde 3-phosphate dehydrogenase (phosphorylating)	1.3667	7.60E-02	-	3.6428	3.91E-07	Up
13	EMIHUDRAFT_421124	GAPDH, glyceraldehyde 3-phosphate dehydrogenase (phosphorylating)	1.1677	1.16E-01	-	3.0873	4.21E-06	Up
14	BGI_novel_G004845	GAPDH, glyceraldehyde 3-phosphate dehydrogenase (phosphorylating)	0.9553	4.10E-01	-	2.0717	1.03E-02	Up
15	BGI_novel_G000759	GAPDH, glyceraldehyde 3-phosphate dehydrogenase (phosphorylating)	-0.3217	7.18E-01	-	1.2146	1.40E-02	Up
16	EMIHUDRAFT_365287	GAPDH, glyceraldehyde 3-phosphate dehydrogenase (phosphorylating)	1.4398	1.37E-10	Up	1.5944	4.62E-09	Up
17	EMIHUDRAFT_216734	GAPDH, glyceraldehyde 3-phosphate dehydrogenase (phosphorylating)	1.4152	1.79E-09	Up	-0.35521	0.3311324	-
18	EMIHUDRAFT_44530	GAPDH, glyceraldehyde 3-phosphate dehydrogenase (phosphorylating)	1.3987	4.67E-02	Up	3.8167	1.23E-16	Up
19	EMIHUDRAFT_365175	PGK, phosphoglycerate kinase	1.5686	1.73E-05	Up	4.4336	5.26E-32	Up
20	EMIHUDRAFT_99818	PGK, phosphoglycerate kinase	1.3566	7.46E-04	Up	2.2278	7.21E-11	Up
21	EMIHUDRAFT_63832	PGK, phosphoglycerate kinase	1.0855	1.30E-02	Up	2.2345	7.86E-12	Up
22	EMIHUDRAFT_417537	PGK, phosphoglycerate kinase	0.9794	9.39E-03	-	3.3104	8.58E-18	Up
23	EMIHUDRAFT_72672	PGK, phosphoglycerate kinase	0.9936	3.86E-01	-	1.9975	6.05E-03	Up
24	EMIHUDRAFT_107385	PGK, phosphoglycerate kinase	0.4539	7.18E-01	-	1.9893	5.57E-03	Up

25	EMIHUDRAFT_428475	GPM, phosphoglycerate mutase	3.0154	7.45E-09	Up	2.3139	4.74E-05	Up
26	EMIHUDRAFT_237246	GPM, phosphoglycerate mutase	1.7446	7.66E-04	Up	3.5516	2.06E-17	Up
27	EMIHUDRAFT_435848	GPM, phosphoglycerate mutase	1.6546	3.04E-08	Up	2.7928	5.65E-20	Up
28	EMIHUDRAFT_428965	GPM, phosphoglycerate mutase	1.2138	3.34E-01	-	2.0454	3.56E-02	Up
29	EMIHUDRAFT_69851	GPM, phosphoglycerate mutase	0.3875	4.52E-01	-	-2.1875	2.15E-05	Down
30	EMIHUDRAFT_74590	GPM, phosphoglycerate mutase	0.3755	2.87E-01	-	-1.3470	3.69E-08	Down
31	EMIHUDRAFT_428531	GPM, phosphoglycerate mutase	0.0209	9.78E-01	-	1.8044	1.33E-13	Up
32	EMIHUDRAFT_110650	GPM, phosphoglycerate mutase	-0.0360	9.77E-01	-	2.2077	1.63E-08	Up
33	EMIHUDRAFT_454452	ENO, enolase	1.6018	4.42E-02	Up	3.6278	2.34E-07	Up
34	EMIHUDRAFT_420746	ENO, enolase	1.2540	6.55E-06	Up	2.5568	1.88E-16	Up
35	EMIHUDRAFT_465926	ENO, enolase	0.4320	1.81E-01	-	1.3617	4.41E-08	Up
36	EMIHUDRAFT_63332	PK, pyruvate kinase	0.5717	5.79E-01	-	2.2613	1.16E-05	Up
37	EMIHUDRAFT_439215	PK, pyruvate kinase	0.1110	8.30E-01	-	1.0853	1.13E-05	Up
38	EMIHUDRAFT_70323	PK, pyruvate kinase	2.3510	1.54E-04	Up	1.8682	5.03E-03	Up
39	EMIHUDRAFT_433474	PK, pyruvate kinase	1.2709	2.02E-03	Up	3.4927	1.09E-17	Up

[a]Log_2 fold change based on RNA-seq data. Each value is the mean from three biological replicates.

[b]Equal to adjusted p-value, change is set at q-value < 0.05 in this study.

Table S11. DEGs involved in TCA cycle in PA/P+ and (P+PA)/P+ comparisons.

No.	Gene ID	Gene name	Description	PA/P+			(P+PA)/P+		
				Log_2 FC[a]	q-value[b]	Regulation	Log_2 FC[a]	q-value[b]	Regulation
1	EMIHUDRAFT_122349	PDHA	K00161//pyruvate dehydrogenase E1 component alpha subunit [EC:1.2.4.1]+ko00010//Glycolysis / Gluconeogenesis+ko00020//Citrate cycle (TCA cycle)+ko01100//Metabolic pathways+ko01110//Biosynthesis of secondary metabolites+ko00620//Pyruvate metabolism+ko01200//Carbon metabolism	0.2267	8.99E-01	-	4.6602	9.47E-12	Up
2	EMIHUDRAFT_359239	PDHA	K00161//pyruvate dehydrogenase E1 component alpha subunit [EC:1.2.4.1]+ko00010//Glycolysis / Gluconeogenesis+ko00020//Citrate cycle (TCA cycle)+ko01100//Metabolic pathways+ko01110//Biosynthesis of secondary metabolites+ko00620//Pyruvate metabolism+ko01200//Carbon metabolism	0.8786	4.13E-04	-	2.3504	1.65E-17	Up
3	EMIHUDRAFT_428425	PDHA	K00161//pyruvate dehydrogenase E1 component alpha subunit [EC:1.2.4.1]+ko00010//Glycolysis / Gluconeogenesis+ko00020//Citrate cycle (TCA cycle)+ko01100//Metabolic pathways+ko01110//Biosynthesis of secondary metabolites+ko00620//Pyruvate metabolism+ko01200//Carbon metabolism	1.1773	5.00E-04	Up	2.6695	1.64E-16	Up
4	EMIHUDRAFT_434751	PDHA	K00162//pyruvate dehydrogenase E1 component beta subunit [EC:1.2.4.1]+ko00010//Glycolysis / Gluconeogenesis+ko00020//Citrate cycle (TCA cycle)+ko01100//Metabolic pathways+ko01110//Biosynthesis of secondary metabolites+ko00620//Pyruvate metabolism+ko01200//Carbon metabolism	0.3140	6.25E-01	-	1.7680	3.96E-07	Up
5	EMIHUDRAFT_444048	PDHA	K00162//pyruvate dehydrogenase E1 component beta subunit [EC:1.2.4.1]+ko00010//Glycolysis / Gluconeogenesis+ko00020//Citrate cycle (TCA cycle)+ko01100//Metabolic pathways+ko01110//Biosynthesis of secondary metabolites+ko00620//Pyruvate metabolism+ko01200//Carbon metabolism	0.6150	4.02E-02	-	1.9191	1.73E-12	Up
6	EMIHUDRAFT_309512	PDHC	K00627//pyruvate dehydrogenase E2 component (dihydrolipoamide acetyltransferase) [EC:2.3.1.12]+ko00010//Glycolysis / Gluconeogenesis+ko00020//Citrate cycle (TCA cycle)+ko01100//Metabolic pathways+ko01110//Biosynthesis of secondary metabolites+ko00620//Pyruvate metabolism+ko01200//Carbon metabolism	0.5874	1.39E-02	-	1.7224	2.43E-11	Up

7	EMIHUDRAFT_448908	PDHC	K00627//pyruvate dehydrogenase E2 component (dihydrolipoamide acetyltransferase) [EC:2.3.1.12]+ko00010//Glycolysis / Gluconeogenesis+ko00020//Citrate cycle (TCA cycle)+ko01100//Metabolic pathways+ko01110//Biosynthesis of secondary metabolites+ko00620//Pyruvate metabolism+ko01200//Carbon metabolism	1.2814	2.55E-03	Up	2.4047	1.02E-12	Up
8	EMIHUDRAFT_62303	PDHC	K00627//pyruvate dehydrogenase E2 component (dihydrolipoamide acetyltransferase) [EC:2.3.1.12]+ko00010//Glycolysis / Gluconeogenesis+ko00020//Citrate cycle (TCA cycle)+ko01100//Metabolic pathways+ko01110//Biosynthesis of secondary metabolites+ko00620//Pyruvate metabolism+ko01200//Carbon metabolism	0.0812	8.97E-01	-	1.2371	4.96E-05	Up
9	EMIHUDRAFT_467883	CS	K01647//citrate synthase [EC:2.3.3.1]+ko00020//Citrate cycle (TCA cycle)+ko01230//Biosynthesis of amino acids+ko01100//Metabolic pathways+ko01110//Biosynthesis of secondary metabolites+ko01210//2-Oxocarboxylic acid metabolism+ko01200//Carbon metabolism+ko00630//Glyoxylate and dicarboxylate metabolism	0.4837	1.72E-01	-	1.6404	5.36E-07	Up
10	EMIHUDRAFT_450594	ACO	K01681//aconitate hydratase [EC:4.2.1.3]+ko00020//Citrate cycle (TCA cycle)+ko01230//Biosynthesis of amino acids+ko01100//Metabolic pathways+ko01110//Biosynthesis of secondary metabolites+ko01210//2-Oxocarboxylic acid metabolism+ko01200//Carbon metabolism+ko00630//Glyoxylate and dicarboxylate metabolism;K09566//peptidyl-prolyl isomerase G (cyclophilin G) [EC:5.2.1.8];K13171//serine/arginine repetitive matrix protein 1+ko03015//mRNA surveillance pathway+ko03013//Nucleocytoplasmic transport	2.3459	1.15E-05	Up	3.1688	4.99E-12	Up
11	EMIHUDRAFT_414497	MDH	K00026//malate dehydrogenase [EC:1.1.1.37]+ko00020//Citrate cycle (TCA cycle)+ko01100//Metabolic pathways+ko01110//Biosynthesis of secondary metabolites+ko00710//Carbon fixation in photosynthetic organisms+ko00620//Pyruvate metabolism+ko00270//Cysteine and methionine metabolism+ko01200//Carbon metabolism+ko00630//Glyoxylate and dicarboxylate metabolism	1.4182	5.65E-03	Up	3.1087	2.18E-15	Up
12	EMIHUDRAFT_448597	MDH	K00026//malate dehydrogenase [EC:1.1.1.37]+ko00020//Citrate cycle (TCA cycle)+ko01100//Metabolic pathways+ko01110//Biosynthesis of secondary metabolites+ko00710//Carbon fixation in photosynthetic organisms+ko00620//Pyruvate metabolism+ko00270//Cysteine and methionine metabolism+ko01200//Carbon metabolism+ko00630//Glyoxylate and dicarboxylate metabolism	1.4660	7.75E-03	Up	2.8074	3.50E-11	Up
13	EMIHUDRAFT_451022	MDH	K00026//malate dehydrogenase [EC:1.1.1.37]+ko00020//Citrate cycle (TCA cycle)+ko01100//Metabolic pathways+ko01110//Biosynthesis of secondary metabolites+ko00710//Carbon fixation in photosynthetic organisms+ko00620//Pyruvate metabolism+ko00270//Cysteine and	1.2094	1.21E-02	Up	1.2740	3.60E-03	Up

	methionine metabolism+ko01200//Carbon metabolism+ko00630//Glyoxylate and dicarboxylate metabolism	

[a]Log_2 fold change based on RNA-seq data. Each value is the mean from three biological replicates.

[b]Equal to adjusted p-value, change is set at q-value < 0.05 in this study.

Table S12. DEGs involved in oxidative phosphorylation in PA/P+ and (P+PA)/P+ comparisons.

No.	Gene ID	Gene name	Description	PA/P+			(P+PA)/P+		
				Log_2 FC[a]	q-value[b]	Regulation	Log_2 FC[a]	q-value[b]	Regulation
1	EMIHUDRAFT_435829	Ndufab1	K03955//NADH dehydrogenase (ubiquinone) 1 alpha/beta subcomplex 1, acyl-carrier protein+ko01100//Metabolic pathways+ko00190//Oxidative phosphorylation	1.2145	1.20E-02	Up	3.0597	4.85E-14	Up
2	EMIHUDRAFT_444042	Ndufab1	K03955//NADH dehydrogenase (ubiquinone) 1 alpha/beta subcomplex 1, acyl-carrier protein+ko01100//Metabolic pathways+ko00190//Oxidative phosphorylation	1.2145	1.20E-02	Up	3.0597	4.85E-14	Up
3	EMIHUDRAFT_443372	Ndufab9	K03965//NADH dehydrogenase (ubiquinone) 1 beta subcomplex subunit 9+ko01100//Metabolic pathways+ko00190//Oxidative phosphorylation	-1.0157	1.57E-04	Down	-0.1233	7.99E-01	-
4	EMIHUDRAFT_441229	Ndufab1	K03955//NADH dehydrogenase (ubiquinone) 1 alpha/beta subcomplex 1, acyl-carrier protein+ko01100//Metabolic pathways+ko00190//Oxidative phosphorylation	0.6022	4.80E-02	-	1.1377	1.15E-04	Up
5	EMIHUDRAFT_459987	Ndufab1	K21776//protein lin-54;K03955//NADH dehydrogenase (ubiquinone) 1 alpha/beta subcomplex 1, acyl-carrier protein+ko01100//Metabolic pathways+ko00190//Oxidative phosphorylation	0.8720	3.30E-02	-	2.2698	6.15E-09	Up
6	EMIHUDRAFT_205539	COX17	K02260//cytochrome c oxidase assembly protein subunit 17+ko01100//Metabolic pathways+ko00190//Oxidative phosphorylation	0.3871	5.75E-01	-	1.3668	3.02E-03	Up
7	EMIHUDRAFT_46771	COX11	K02258//cytochrome c oxidase assembly protein subunit 11+ko01100//Metabolic pathways+ko00190//Oxidative phosphorylation	-1.1676	4.34E-03	Down	-0.2773	5.52E-01	-
8	EMIHUDRAFT_436606	CYC	K08738//cytochrome c+ko01100//Metabolic pathways+ko00190//Oxidative phosphorylation	1.0273	3.34E-06	Up	1.2318	3.88E-05	Up
9	EMIHUDRAFT_431150	CYC	K08738//cytochrome c+ko01100//Metabolic pathways+ko00190//Oxidative phosphorylation	0.9046	2.48E-04	-	1.2349	1.80E-05	Up
10	EMIHUDRAFT_352051	CYC	K08738//cytochrome c+ko01100//Metabolic pathways+ko00190//Oxidative phosphorylation	0.6504	4.73E-01	-	2.0941	4.06E-04	Up
11	EMIHUDRAFT_352094	CYC	K08738//cytochrome c+ko01100//Metabolic pathways+ko00190//Oxidative phosphorylation	0.1455	8.93E-01	-	1.8106	2.37E-05	Up
12	EMIHUDRAFT_352094	CYC	K08738//cytochrome c+ko01100//Metabolic pathways+ko00190//Oxidative phosphorylation	0.1455	8.93E-01	-	1.8106	2.37E-05	Up
13	BGI_novel_G001822	F-type ATPase beta	K02133//F-type H+-transporting ATPase subunit beta [EC:7.1.2.2]+ko01100//Metabolic pathways+ko00190//Oxidative phosphorylation	1.1145	2.12E-05	Up	2.0243	1.20E-12	Up

14	EMIHUDRAFT_433142	F-type ATPase alpha	K02132//F-type H+-transporting ATPase subunit alpha+ko01100//Metabolic pathways+ko00190//Oxidative phosphorylation	0.9838	2.53E-06	-	2.1159	8.46E-16	Up
15	BGI_novel_G004112	F-type ATPase beta	K02133//F-type H+-transporting ATPase subunit beta [EC:7.1.2.2]+ko01100//Metabolic pathways+ko00190//Oxidative phosphorylation	0.8656	2.46E-04	-	1.6820	8.46E-13	Up
16	BGI_novel_G001821	F-type ATPase beta	K02133//F-type H+-transporting ATPase subunit beta [EC:7.1.2.2]+ko01100//Metabolic pathways+ko00190//Oxidative phosphorylation	0.8335	3.40E-04	-	1.8278	2.66E-09	Up
17	EMIHUDRAFT_442968	F-type ATPase gamma	K02136//F-type H+-transporting ATPase subunit gamma+ko01100//Metabolic pathways+ko00190//Oxidative phosphorylation	0.0004	1.00E+00	-	1.4204	2.79E-05	Up
18	EMIHUDRAFT_70361	F-type ATPase delta	K02134//F-type H+-transporting ATPase subunit delta+ko01100//Metabolic pathways+ko00190//Oxidative phosphorylation	-0.1162	9.21E-01	-	1.6078	4.12E-04	Up
19	EMIHUDRAFT_456269	F-type ATPase OSCP	K02137//F-type H+-transporting ATPase subunit O+ko01100//Metabolic pathways+ko00190//Oxidative phosphorylation	0.4604	1.47E-01	-	1.5554	5.74E-09	Up
20	EMIHUDRAFT_442547	V-type ATPase B	K02147//V-type H+-transporting ATPase subunit B+ko04145//Phagosome+ko01100//Metabolic pathways+ko00190//Oxidative phosphorylation	1.0936	1.68E-06	Up	2.5252	5.04E-20	Up
21	EMIHUDRAFT_439538	V-type ATPase A	K02145//V-type H+-transporting ATPase subunit A [EC:7.1.2.2]+ko04145//Phagosome+ko01100//Metabolic pathways+ko00190//Oxidative phosphorylation	1.0883	3.87E-03	Up	2.4950	1.41E-11	Up
22	EMIHUDRAFT_413949	V-type ATPase d	K02146//V-type H+-transporting ATPase subunit d+ko04145//Phagosome+ko01100//Metabolic pathways+ko00190//Oxidative phosphorylation	0.9431	2.31E-04	-	1.8558	4.28E-10	Up
23	EMIHUDRAFT_444698	V-type ATPase c	K02155//V-type H+-transporting ATPase 16kDa proteolipid subunit+ko04145//Phagosome+ko01100//Metabolic pathways+ko00190//Oxidative phosphorylation;K14573//nucleolar protein 4+ko03008//Ribosome biogenesis in eukaryotes	0.9243	6.32E-04	-	1.5602	4.28E-10	Up
24	EMIHUDRAFT_95543	V-type ATPase H	K02144//V-type H+-transporting ATPase subunit H+ko04145//Phagosome+ko01100//Metabolic pathways+ko00190//Oxidative phosphorylation;K11323//histone arginine demethylase JMJD6 [EC:1.14.11.-]	0.9099	4.90E-02	-	2.8782	5.66E-17	Up
25	BGI_novel_G004718	V-type ATPase E	K02150//V-type H+-transporting ATPase subunit E+ko04145//Phagosome+ko01100//Metabolic pathways+ko00190//Oxidative phosphorylation	0.7475	4.02E-01	-	3.3080	8.47E-12	Up
26	EMIHUDRAFT_433060	V-type ATPase E	K02150//V-type H+-transporting ATPase subunit E+ko04145//Phagosome+ko01100//Metabolic pathways+ko00190//Oxidative	0.6889	7.91E-02	-	2.4816	7.29E-14	Up

			phosphorylation;K14325//RNA-binding protein with serine-rich domain 1+ko03015//mRNA surveillance pathway+ko03013//Nucleocytoplasmic transport;K12891//serine/arginine-rich splicing factor 2/8+ko03040//Spliceosome						
27	EMIHUDRAFT_362459	V-type ATPase c	K02155//V-type H+-transporting ATPase 16kDa proteolipid subunit+ko04145//Phagosome+ko01100//Metabolic pathways+ko00190//Oxidative phosphorylation	0.6799	1.96E-02	-	1.8210	4.06E-15	Up
28	EMIHUDRAFT_61253	V-type ATPase a	K02154//V-type H+-transporting ATPase subunit a+ko04145//Phagosome+ko01100//Metabolic pathways+ko00190//Oxidative phosphorylation	0.5821	2.83E-02	-	1.5879	2.69E-13	Up
29	EMIHUDRAFT_366512	V-type ATPase c	K02155//V-type H+-transporting ATPase 16kDa proteolipid subunit+ko04145//Phagosome+ko01100//Metabolic pathways+ko00190//Oxidative phosphorylation	0.3737	4.04E-01	-	1.2948	7.40E-07	Up
30	EMIHUDRAFT_369392	V-type ATPase G	K02152//V-type H+-transporting ATPase subunit G+ko04145//Phagosome+ko01100//Metabolic pathways+ko00190//Oxidative phosphorylation	0.2605	5.80E-01	-	1.8104	2.30E-08	Up
31	EMIHUDRAFT_313800	V-type ATPase c	K03661//V-type H+-transporting ATPase 21kDa proteolipid subunit+ko04145//Phagosome+ko01100//Metabolic pathways+ko00190//Oxidative phosphorylation	0.1586	8.13E-01	-	1.1221	5.78E-05	Up
32	EMIHUDRAFT_313422	V-type ATPase c	K02155//V-type H+-transporting ATPase 16kDa proteolipid subunit+ko04145//Phagosome+ko01100//Metabolic pathways+ko00190//Oxidative phosphorylation	0.1293	8.30E-01	-	1.4071	3.11E-06	Up
33	EMIHUDRAFT_451883	V-type ATPase c	K03661//V-type H+-transporting ATPase 21kDa proteolipid subunit+ko04145//Phagosome+ko01100//Metabolic pathways+ko00190//Oxidative phosphorylation	0.1236	8.20E-01	-	1.2301	8.93E-06	Up
34	EMIHUDRAFT_359783	V-type ATPase c	K02155//V-type H+-transporting ATPase 16kDa proteolipid subunit+ko04145//Phagosome+ko01100//Metabolic pathways+ko00190//Oxidative phosphorylation	0.0524	9.31E-01	-	1.1052	1.51E-03	Up
35	EMIHUDRAFT_352209	V-type ATPase F	K02151//V-type H+-transporting ATPase subunit F+ko04145//Phagosome+ko01100//Metabolic pathways+ko00190//Oxidative phosphorylation	-0.0582	9.36E-01	-	1.5226	3.64E-06	Up
36	EMIHUDRAFT_216523	V-type ATPase e	K02153//V-type H+-transporting ATPase subunit e+ko04145//Phagosome+ko01100//Metabolic pathways+ko00190//Oxidative phosphorylation	-0.0856	9.42E-01	-	1.2078	1.47E-02	Up
37	EMIHUDRAFT_220717	V-type ATPase e	K02153//V-type H+-transporting ATPase subunit e+ko04145//Phagosome+ko01100//Metabolic pathways+ko00190//Oxidative phosphorylation	-0.0856	9.42E-01	-	1.2078	1.47E-02	Up
38	EMIHUDRAFT_370527	V-type ATPase c	K02155//V-type H+-transporting ATPase 16kDa proteolipid subunit+ko04145//Phagosome+ko01100//Metabolic pathways+ko00190//Oxidative phosphorylation	-0.3310	8.40E-01	-	1.5530	1.84E-02	Up
39	BGI_novel_G001233	H+-transpo	K01535//H+-transporting ATPase [EC:7.1.2.1]+ko01100//Metabolic	0.0620	9.77E-01	-	1.8898	1.25E-02	Up

		rting ATPase	pathways+ko00190//Oxidative phosphorylation;K15865//threonylcarbamoyladenosine tRNA methylthiotransferase CDKAL1 [EC:2.8.4.5];K01309//ubiquitin carboxyl-terminal hydrolase MINDY-1/2 [EC:3.4.19.12]						
40	EMIHUDRAFT_70025	H+-transporting ATPase	K01535//H+-transporting ATPase [EC:7.1.2.1]+ko01100//Metabolic pathways+ko00190//Oxidative phosphorylation	1.1236	6.30E-04	Up	1.7295	1.56E-09	Up
41	BGI_novel_G003434	H+-transporting ATPase	K01535//H+-transporting ATPase [EC:7.1.2.1]+ko01100//Metabolic pathways+ko00190//Oxidative phosphorylation;K01309//ubiquitin carboxyl-terminal hydrolase MINDY-1/2 [EC:3.4.19.12]	-0.4683	2.56E-01	-	1.0191	1.48E-03	Up
42	EMIHUDRAFT_67081	H+-transporting ATPase	K01535//H+-transporting ATPase [EC:7.1.2.1]+ko01100//Metabolic pathways+ko00190//Oxidative phosphorylation	0.6648	5.40E-01	-	1.5498	1.02E-02	Up
43	EMIHUDRAFT_253209	H+-transporting ATPase	K09566//peptidyl-prolyl isomerase G (cyclophilin G) [EC:5.2.1.8];K09667//protein O-GlcNAc transferase [EC:2.4.1.255]+ko00514//Other types of O-glycan biosynthesis;K14676//lysophospholipid hydrolase [EC:3.1.1.5]+ko00564//Glycerophospholipid metabolism;K01535//H+-transporting ATPase [EC:7.1.2.1]+ko01100//Metabolic pathways+ko00190//Oxidative phosphorylation;K21952//CASK-interacting protein	0.1573	7.72E-01	-	-1.3653	3.98E-05	Down
44	BGI_novel_G003883	H+-transporting ATPase	K01535//H+-transporting ATPase [EC:7.1.2.1]+ko01100//Metabolic pathways+ko00190//Oxidative phosphorylation;K14709//solute carrier family 39 (zinc transporter), member 1/2/3	-0.0874	9.21E-01	-	1.1774	3.33E-03	Up
45	EMIHUDRAFT_426283	H+-transporting ATPase	K01535//H+-transporting ATPase [EC:7.1.2.1]+ko01100//Metabolic pathways+ko00190//Oxidative phosphorylation	2.9837169	0.0004048	Up	0.651032	0.6984051	-
46	EMIHUDRAFT_436192	PPA	K01507//inorganic pyrophosphatase [EC:3.6.1.1]+ko00190//Oxidative phosphorylation	0.7631	1.08E-01	-	2.0748	6.00E-09	Up
47	EMIHUDRAFT_439901	PPA	K01507//inorganic pyrophosphatase [EC:3.6.1.1]+ko00190//Oxidative phosphorylation	0.4766	1.18E-01	-	1.9905	1.20E-13	Up
48	EMIHUDRAFT_415968	PPA	K01507//inorganic pyrophosphatase [EC:3.6.1.1]+ko00190//Oxidative phosphorylation	0.4147	4.46E-01	-	2.0778	5.90E-08	Up

[a]Log$_2$ fold change based on RNA-seq data. Each value is the mean from three biological replicates.

[b]Equal to adjusted p-value, change is set at q-value < 0.05 in this study.

Table S13. DEGs involved in glycerophospholipid in PA/P+ and (P+PA)/P+ comparisons.

No.	Gene ID	Description	PA/P+			(P+PA)/P+		
			Log_2 FC[a]	q-value[b]	Regulation	Log_2 FC[a]	q-value[b]	Regulation
1	EMIHU DRAFT_ 104852	K11649//SWI/SNF related-matrix-associated actin-dependent regulator of chromatin subfamily C;K20547//basic endochitinase B [EC:3.2.1.14]+ko04016//MAPK signaling pathway - plant+ko00520//Amino sugar and nucleotide sugar metabolism+ko01100//Metabolic pathways;K17506//protein phosphatase 1L [EC:3.1.3.16]; K14676//lysophospholipid hydrolase [EC:3.1.1.5]+ko00564//Glycerophospholipid metabolism	3.8226	1.10E-09	Up	3.8555	2.04E-09	Up
2	EMIHU DRAFT_ 100213	K02184//formin 2;K18757//la-related protein 1;K15865//threonylcarbamoyladenosine tRNA methylthiotransferase CDKAL1 [EC:2.8.4.5];K14676//lysophospholipid hydrolase [EC:3.1.1.5]+ko00564//Glycerophospholipid metabolism	2.1394	3.56E-02	Up	2.1783	4.24E-02	Up
3	EMIHU DRAFT_ 451667	K13171//serine/arginine repetitive matrix protein 1+ko03015//mRNA surveillance pathway+ko03013//Nucleocytoplasmic transport;K17987//next to BRCA1 gene 1 protein;K14676//lysophospholipid hydrolase [EC:3.1.1.5]+ko00564//Glycerophospholipid metabolism	1.8338	5.38E-02	Up	3.9533	3.14E-09	Up
4	EMIHU DRAFT_ 102546	K13172//serine/arginine repetitive matrix protein 2;K14317//nuclear pore complex protein Nup214+ko03013//Nucleocytoplasmic transport; K14676//lysophospholipid hydrolase [EC:3.1.1.5]+ko00564//Glycerophospholipid metabolism	1.7866	1.14E-07	Up	2.2925	2.59E-13	Up
5	EMIHU DRAFT_ 210675	K13412//calcium-dependent protein kinase [EC:2.7.11.1]+ko04626//Plant-pathogen interaction;K14306//nuclear pore complex protein Nup62+ko03013//Nucleocytoplasmic transport;K01051//pectinesterase [EC:3.1.1.11]+ko00040//Pentose and glucuronate interconversions+ko01100//Metabolic pathways;K03927//carboxylesterase 2 [EC:3.1.1.1 3.1.1.84 3.1.1.56];K01049//acetylcholinesterase [EC:3.1.1.7]+ko00564//Glycerophospholipid metabolism	1.5817	2.47E-10	Up	1.3846	2.77E-08	Up
6	EMIHU DRAFT_ 455833	K00630//glycerol-3-phosphate O-acyltransferase [EC:2.3.1.15]+ko01100//Metabolic pathways+ko00564//Glycerophospholipid metabolism+ko00561//Glycerolipid metabolism	1.5056	6.58E-02	-	2.7466	1.75E-06	Up
7	EMIHU DRAFT_ 455603	K00111//glycerol-3-phosphate dehydrogenase [EC:1.1.5.3]+ko01110//Biosynthesis of secondary metabolites+ko00564//Glycerophospholipid metabolism	1.4800	4.83E-02	Up	2.9845	9.13E-06	Up

8	EMIHU DRAFT_ 434929	K13171//serine/arginine repetitive matrix protein 1+ko03015//mRNA surveillance pathway+ko03013//Nucleocytoplasmic transport;K14676//lysophospholipid hydrolase [EC:3.1.1.5]+ko00564//Glycerophospholipid metabolism	1.4655	2.29E-08	Up	-0.8998	6.49E-03	-
9	EMIHU DRAFT_ 427769	K00006//glycerol-3-phosphate dehydrogenase (NAD+) [EC:1.1.1.8]+ko01110//Biosynthesis of secondary metabolites+ko00564//Glycerophospholipid metabolism	1.4147	2.58E-05	Up	2.2470	4.66E-14	Up
10	EMIHU DRAFT_ 450840	K00111//glycerol-3-phosphate dehydrogenase [EC:1.1.5.3]+ko01110//Biosynthesis of secondary metabolites+ko00564//Glycerophospholipid metabolism	1.2860	7.31E-03	Up	2.6540	6.11E-10	Up
11	EMIHU DRAFT_ 441121	K00630//glycerol-3-phosphate O-acyltransferase [EC:2.3.1.15]+ko01100//Metabolic pathways+ko00564//Glycerophospholipid metabolism+ko00561//Glycerolipid metabolism	1.1739	3.39E-02	Up	2.6194	1.07E-09	Up
12	EMIHU DRAFT_ 235704	K13171//serine/arginine repetitive matrix protein 1+ko03015//mRNA surveillance pathway+ko03013//Nucleocytoplasmic transport;K10747//DNA ligase 1 [EC:6.5.1.1 6.5.1.6 6.5.1.7]+ko03420//Nucleotide excision repair+ko03030//DNA replication+ko03410//Base excision repair+ko03430//Mismatch repair;K14676//lysophospholipid hydrolase [EC:3.1.1.5]+ko00564//Glycerophospholipid metabolism	1.1426	2.70E-02	Up	2.6631	1.09E-17	Up
13	EMIHU DRAFT_ 432864	K00006//glycerol-3-phosphate dehydrogenase (NAD+) [EC:1.1.1.8]+ko01110//Biosynthesis of secondary metabolites+ko00564//Glycerophospholipid metabolism	1.1267	3.27E-03	Up	2.9553	5.60E-15	Up
14	EMIHU DRAFT_ 467948	K00550//phosphatidyl-N-methylethanolamine N-methyltransferase [EC:2.1.1.71]+ko01100//Metabolic pathways+ko01110//Biosynthesis of secondary metabolites+ko00564//Glycerophospholipid metabolism;K14325//RNA-binding protein with serine-rich domain 1+ko03015//mRNA surveillance pathway+ko03013//Nucleocytoplasmic transport;K13680//beta-mannan synthase [EC:2.4.1.32]	1.0696	1.12E-02	Up	1.0615	5.08E-03	Up
15	EMIHU DRAFT_ 228272	K09122//uncharacterized protein;K02184//formin 2;K14676//lysophospholipid hydrolase [EC:3.1.1.5]+ko00564//Glycerophospholipid metabolism	0.8841	1.48E-01	-	1.5362	1.23E-03	Up
16	EMIHU DRAFT_ 433779	K01126//glycerophosphoryl diester phosphodiesterase [EC:3.1.4.46]+ko00564//Glycerophospholipid metabolism;K14306//nuclear pore complex protein Nup62+ko03013//Nucleocytoplasmic transport	0.8239	1.58E-04	-	-1.6373	1.95E-08	Down
17	EMIHU DRAFT_ 450556	K13172//serine/arginine repetitive matrix protein 2;K14676//lysophospholipid hydrolase [EC:3.1.1.5]+ko00564//Glycerophospholipid metabolism;K13171//serine/arginine repetitive matrix protein 1+ko03015//mRNA surveillance pathway+ko03013//Nucleocytoplasmic transport;K02184//formin 2	0.7808	4.39E-02	-	1.6856	1.08E-08	Up

18	EMIHU DRAFT_ 104459	K13508//glycerol-3-phosphate acyltransferase [EC:2.3.1.15 2.3.1.198]+ko01100//Metabolic pathways+ko01110//Biosynthesis of secondary metabolites+ko00564//Glycerophospholipid metabolism+ko00561//Glycerolipid metabolism	0.6688	2.90E-01	-	1.0057	3.08E-02	Up
19	EMIHU DRAFT_ 236039	K13172//serine/arginine repetitive matrix protein 2;K14325//RNA-binding protein with serine-rich domain 1+ko03015//mRNA surveillance pathway+ko03013//Nucleocytoplasmic transport;K06236//collagen type I alpha;K03128//transcription initiation factor TFIID subunit 2+ko03022//Basal transcription factors;K14676//lysophospholipid hydrolase [EC:3.1.1.5]+ko00564//Glycerophospholipid metabolism	0.6458	3.03E-01	-	1.9765	5.57E-10	Up
20	EMIHU DRAFT_ 460094	K00006//glycerol-3-phosphate dehydrogenase (NAD+) [EC:1.1.1.8]+ko01110//Biosynthesis of secondary metabolites+ko00564//Glycerophospholipid metabolism	0.6343	2.90E-01	-	2.4244	1.27E-07	Up
21	EMIHU DRAFT_ 98168	K00967//ethanolamine-phosphate cytidylyltransferase [EC:2.7.7.14]+ko00440//Phosphonate and phosphinate metabolism+ko01100//Metabolic pathways+ko00564//Glycerophospholipid metabolism	0.6004	5.91E-01	-	1.5082	1.67E-02	Up
22	EMIHU DRAFT_ 430429	K21594//translation factor GUF1, mitochondrial [EC:3.6.5.-];K13171//serine/arginine repetitive matrix protein 1+ko03015//mRNA surveillance pathway+ko03013//Nucleocytoplasmic transport; K01517//manganese-dependent ADP-ribose/CDP-alcohol diphosphatase [EC:3.6.1.13 3.6.1.16 3.6.1.53]+ko00230//Purine metabolism+ko01100//Metabolic pathways+ko00564//Glycerophospholipid metabolism	0.5853	1.43E-01	-	1.2111	1.60E-04	Up
23	EMIHU DRAFT_ 415940	K00006//glycerol-3-phosphate dehydrogenase (NAD+) [EC:1.1.1.8]+ko01110//Biosynthesis of secondary metabolites+ko00564//Glycerophospholipid metabolism	0.5302	1.27E-01	-	2.1811	3.35E-11	Up
24	EMIHU DRAFT_ 217317	K00006//glycerol-3-phosphate dehydrogenase (NAD+) [EC:1.1.1.8]+ko01110//Biosynthesis of secondary metabolites+ko00564//Glycerophospholipid metabolism	0.5046	2.62E-01	-	1.6168	1.67E-09	Up
25	EMIHU DRAFT_ 444387	K03453//bile acid:Na+ symporter, BASS family;K14676//lysophospholipid hydrolase [EC:3.1.1.5]+ko00564//Glycerophospholipid metabolism	0.2715	7.49E-01	-	1.2273	2.83E-03	Up
26	BGI_nov el_G001 763	K17987//next to BRCA1 gene 1 protein;K14676//lysophospholipid hydrolase [EC:3.1.1.5]+ko00564//Glycerophospholipid metabolism;K06941//23S rRNA (adenine2503-C2)-methyltransferase [EC:2.1.1.192]	0.2397	7.97E-01	-	1.2366	1.81E-03	Up
27	EMIHU DRAFT_ 463495	K13171//serine/arginine repetitive matrix protein 1+ko03015//mRNA surveillance pathway+ko03013//Nucleocytoplasmic transport;K01180//endo-1,3(4)-beta-glucanase [EC:3.2.1.6];K10747//DNA ligase 1 [EC:6.5.1.1 6.5.1.6 6.5.1.7]+ko03420//Nucleotide excision repair+ko03030//DNA replication+ko03410//Base excision repair+ko03430//Mismatch	0.1847	7.98E-01	-	1.0877	6.90E-04	Up

		repair;K14676//lysophospholipid hydrolase [EC:3.1.1.5]+ko00564//Glycerophospholipid metabolism						
28	EMIHU DRAFT_360495	K06276//3-phosphoinositide dependent protein kinase-1 [EC:2.7.11.1];K14676//lysophospholipid hydrolase [EC:3.1.1.5]+ko00564//Glycerophospholipid metabolism;K21952//CASK-interacting protein	0.1777	5.85E-01	-	-1.0316	1.72E-07	Down
29	EMIHU DRAFT_253209	K09566//peptidyl-prolyl isomerase G (cyclophilin G) [EC:5.2.1.8];K09667//protein O-GlcNAc transferase [EC:2.4.1.255]+ko00514//Other types of O-glycan biosynthesis;K14676//lysophospholipid hydrolase [EC:3.1.1.5]+ko00564//Glycerophospholipid metabolism;K01535//H+-transporting ATPase [EC:7.1.2.1]+ko01100//Metabolic pathways+ko00190//Oxidative phosphorylation;K21952//CASK-interacting protein	0.1573	7.72E-01	-	-1.3653	3.98E-05	Down
30	EMIHU DRAFT_212319	K09566//peptidyl-prolyl isomerase G (cyclophilin G) [EC:5.2.1.8];K13172//serine/arginine repetitive matrix protein 2;K08776//puromycin-sensitive aminopeptidase [EC:3.4.11.14];K14676//lysophospholipid hydrolase [EC:3.1.1.5]+ko00564//Glycerophospholipid metabolism;K11367//chromodomain-helicase-DNA-binding protein 1 [EC:3.6.4.12]	0.0821	8.74E-01	-	-1.6862	2.09E-10	Down
31	EMIHU DRAFT_237240	K13171//serine/arginine repetitive matrix protein 1+ko03015//mRNA surveillance pathway+ko03013//Nucleocytoplasmic transport;K13519//lysophospholipid acyltransferase [EC:2.3.1.51 2.3.1.23 2.3.1.-]+ko01100//Metabolic pathways+ko01110//Biosynthesis of secondary metabolites+ko00564//Glycerophospholipid metabolism+ko00565//Ether lipid metabolism+ko00561//Glycerolipid metabolism;K03128//transcription initiation factor TFIID subunit 2+ko03022//Basal transcription factors;K14972//PAX-interacting protein 1;K13172//serine/arginine repetitive matrix protein 2;K15223//upstream activation factor subunit UAF30	0.0079	9.91E-01	-	-1.0845	2.67E-03	Down
32	BGI_novel_G004320	K14306//nuclear pore complex protein Nup62+ko03013//Nucleocytoplasmic transport;K14676//lysophospholipid hydrolase [EC:3.1.1.5]+ko00564//Glycerophospholipid metabolism	-0.0727	9.60E-01	-	1.2847	7.01E-03	Up
33	EMIHU DRAFT_466638	K14618//cyanocobalamin reductase (cyanide-eliminating) / alkylcobalamin dealkylase [EC:1.16.1.6 2.5.1.151];K13172//serine/arginine repetitive matrix protein 2;K17987//next to BRCA1 gene 1 protein;K14618//cyanocobalamin reductase (cyanide-eliminating) / alkylcobalamin dealkylase [EC:1.16.1.6 2.5.1.151];K17987//next to BRCA1 gene 1 protein;K14676//lysophospholipid hydrolase [EC:3.1.1.5]+ko00564//Glycerophospholipid metabolism	-0.1155	8.90E-01	-	1.1275	9.85E-04	Up

34	BGI_novel_G000733	K01051//pectinesterase [EC:3.1.1.11]+ko00040//Pentose and glucuronate interconversions+ko01100//Metabolic pathways;K14676//lysophospholipid hydrolase [EC:3.1.1.5]+ko00564//Glycerophospholipid metabolism	-0.3302	6.99E-01	-	-1.7403	1.63E-03	Down
35	BGI_novel_G002304	K14676//lysophospholipid hydrolase [EC:3.1.1.5]+ko00564//Glycerophospholipid metabolism	-0.3903	2.89E-01	-	-1.5035	1.40E-08	Down
36	EMIHUDRAFT_462425	K21952//CASK-interacting protein;K01126//glycerophosphoryl diester phosphodiesterase [EC:3.1.4.46]+ko00564//Glycerophospholipid metabolism	-0.3932	2.12E-01	-	-1.1047	7.56E-05	Down
37	EMIHUDRAFT_442191	K13509//lysophosphatidate acyltransferase [EC:2.3.1.51]+ko01100//Metabolic pathways+ko01110//Biosynthesis of secondary metabolites+ko00564//Glycerophospholipid metabolism+ko00561//Glycerolipid metabolism;K06236//collagen type I alpha;K00655//1-acyl-sn-glycerol-3-phosphate acyltransferase [EC:2.3.1.51]+ko01100//Metabolic pathways+ko01110//Biosynthesis of secondary metabolites+ko00564//Glycerophospholipid metabolism+ko00561//Glycerolipid metabolism	-0.5470	2.23E-01	-	-2.4791	1.25E-11	Down
38	EMIHUDRAFT_208336	K15728//phosphatidate phosphatase LPIN [EC:3.1.3.4]+ko01100//Metabolic pathways+ko01110//Biosynthesis of secondary metabolites+ko00564//Glycerophospholipid metabolism+ko00561//Glycerolipid metabolism;K03125//transcription initiation factor TFIID subunit 1 [EC:2.3.1.48 2.7.11.1]+ko03022//Basal transcription factors	-0.5820	2.36E-01	-	-1.3611	4.38E-04	Down
39	EMIHUDRAFT_219336	K14676//lysophospholipid hydrolase [EC:3.1.1.5]+ko00564//Glycerophospholipid metabolism;K12121//phytochrome B+ko04712//Circadian rhythm - plant;K21952//CASK-interacting protein;K13420//LRR receptor-like serine/threonine-protein kinase FLS2 [EC:2.7.11.1]+ko04016//MAPK signaling pathway - plant+ko04626//Plant-pathogen interaction	-0.8180	1.51E-01	-	-2.2604	3.94E-06	Down
40	EMIHUDRAFT_445817	K00999//CDP-diacylglycerol--inositol 3-phosphatidyltransferase [EC:2.7.8.11]+ko01100//Metabolic pathways+ko04070//Phosphatidylinositol signaling system+ko00564//Glycerophospholipid metabolism+ko00562//Inositol phosphate metabolism	-0.9254	4.40E-04	-	-1.1667	3.47E-07	Down
41	EMIHUDRAFT_224889	K13513//lysocardiolipin and lysophospholipid acyltransferase [EC:2.3.1.- 2.3.1.51]+ko01100//Metabolic pathways+ko01110//Biosynthesis of secondary metabolites+ko00564//Glycerophospholipid metabolism+ko00561//Glycerolipid metabolism	-2.5175	1.34E-02	Down	-3.1634	2.91E-03	Down
42	EMIHUDRAFT_222113	K00679//phospholipid:diacylglycerol acyltransferase [EC:2.3.1.158]+ko01100//Metabolic pathways+ko00561//Glycerolipid metabolism;K00650//lecithin-cholesterol acyltransferase [EC:2.3.1.43]+ko00564//Glycerophospholipid metabolism	2.2025	4.39E-03	Up	1.6180	6.05E-02	-

43	EMIHU DRAFT_ 237284	K13510//lysophosphatidylcholine acyltransferase / lyso-PAF acetyltransferase [EC:2.3.1.23 2.3.1.67]+ko01100//Metabolic pathways+ko00564//Glycerophospholipid metabolism+ko00565//Ether lipid metabolism; K09566//peptidyl-prolyl isomerase G (cyclophilin G) [EC:5.2.1.8]	1.6168	4.00E-13	Up	0.3309	3.32E-01	-
44	EMIHU DRAFT_ 425971	K14676//lysophospholipid hydrolase [EC:3.1.1.5]+ko00564//Glycerophospholipid metabolism	1.5449	1.74E-09	Up	-0.2479	5.61E-01	-
45	EMIHU DRAFT_ 424485	K14676//lysophospholipid hydrolase [EC:3.1.1.5]+ko00564//Glycerophospholipid metabolism	-1.0196	1.39E-05	Down	-0.3070	2.42E-01	-
46	EMIHU DRAFT_ 99558	K14676//lysophospholipid hydrolase [EC:3.1.1.5]+ko00564//Glycerophospholipid metabolism;K17987//next to BRCA1 gene 1 protein;K03128//transcription initiation factor TFIID subunit 2+ko03022//Basal transcription factors	-1.0458	1.79E-02	Down	-0.0561	9.24E-01	-
47	EMIHU DRAFT_ 463200	K14676//lysophospholipid hydrolase [EC:3.1.1.5]+ko00564//Glycerophospholipid metabolism;K13172//serine/arginine repetitive matrix protein 2;K03128//transcription initiation factor TFIID subunit 2+ko03022//Basal transcription factors	-1.0618	1.25E-03	Down	0.3216	2.42E-01	-
48	EMIHU DRAFT_ 459761	K14674//TAG lipase / steryl ester hydrolase / phospholipase A2 / LPA acyltransferase [EC:3.1.1.3 3.1.1.13 3.1.1.4 2.3.1.51]+ko00591//Linoleic acid metabolism+ko00590//Arachidonic acid metabolism+ko00592//alpha-Linolenic acid metabolism+ko01100//Metabolic pathways+ko01110//Biosynthesis of secondary metabolites+ko00100//Steroid biosynthesis+ko00564//Glycerophospholipid metabolism+ko00565//Ether lipid metabolism+ko00561//Glycerolipid metabolism	-1.1643	2.18E-02	Down	0.1465	7.97E-01	-
49	EMIHU DRAFT_ 202596	K11314//transcriptional adapter 2-alpha;K14676//lysophospholipid hydrolase [EC:3.1.1.5]+ko00564//Glycerophospholipid metabolism	-1.2021	2.05E-05	Down	-0.0722	8.53E-01	-
50	EMIHU DRAFT_ 74149	K06130//lysophospholipase II [EC:3.1.1.5]+ko00564//Glycerophospholipid metabolism;K06128//lysophospholipase I [EC:3.1.1.5]+ko00564//Glycerophospholipid metabolism	-1.5466	5.74E-05	Down	-0.5880	8.07E-02	-
51	EMIHU DRAFT_ 71725	K06130//lysophospholipase II [EC:3.1.1.5]+ko00564//Glycerophospholipid metabolism;K06128//lysophospholipase I [EC:3.1.1.5]+ko00564//Glycerophospholipid metabolism	-1.5466	5.74E-05	Down	-0.5880	8.07E-02	-
52	EMIHU DRAFT_ 115922	K13172//serine/arginine repetitive matrix protein 2;K14676//lysophospholipid hydrolase [EC:3.1.1.5]+ko00564//Glycerophospholipid metabolism	-1.5927	2.93E-02	Down	-0.8324	1.94E-01	-

[a]Log_2 fold change based on RNA-seq data. Each value is the mean from three biological replicates.

[b]Equal to adjusted p-value, change is set at q-value < 0.05 in this study.
